# Triple arm, prospective, real-world study comparing the efficacy of FDC teneligliptin + dapagliflozin to FDC sitagliptin + dapagliflozin, and FDC linagliptin + empagliflozin in Indian type 2 diabetes mellitus patients using CGM device: the Amplify-TIR study

**DOI:** 10.1186/s40842-025-00244-6

**Published:** 2025-11-26

**Authors:** Suhas Erande, Mayur Agrawal, Sanjeev Gulati, Namdev Jagtap, N. S. Praveen Kumar, Vinod Kumar Kapoor, Sumit Bhushan, Rujuta Gadkari, Mayur Jadhav, Sanjay Choudhari, Saiprasad Patil, Hanmant Barkate

**Affiliations:** 1 Akshay Hospital, Pune, India; 2 Hormone India Diabetes and Endocrine Centre, Bhopal, India; 3 Sarvottam Hospital, Bhopal, India; 4 Spandan Multispecialty Clinic, Pune, India; 5 Mysore Medical College, Mysore, India; 6 New Leelamani Hospital, Kanpur, India; 7 Glenmark Pharmaceuticals, Mumbai, India

**Keywords:** Type 2 diabetes mellitus, Glycemic variability, Time in range, Teneligliptin, Dapagliflozin, Sitagliptin, Empagliflozin, Continuous glucose monitoring, HbA1c, Renal function

## Abstract

**Introduction:**

Diabetes mellitus (DM) is a chronic metabolic disorder marked by persistent hyperglycemia. While HbA1c has traditionally been used to assess glycemic control, growing evidence highlights glycemic variability (GV) and Time in Range (TIR) as more precise indicators of glucose fluctuations, which are linked to diabetic complications, especially chronic kidney disease (CKD). Emerging combination therapies targeting different pathophysiologic mechanisms of type 2 diabetes mellitus (T2DM), such as SGLT2 inhibitors and DPP-4 inhibitors, offer promise in reducing GV.

**Objective:**

To compare the efficacy of three commonly prescribed fixed-dose combination (FDC) therapies—Teneligliptin + Dapagliflozin (Arm-A), Sitagliptin + Dapagliflozin (Arm-B), and Linagliptin + Empagliflozin (Arm-C)—in improving glycemic control and renal parameters in Indian T2DM patients using continuous glucose monitoring (CGM).

**Method:**

This prospective, comparative study enrolled 90 patients (30 in each arm). CGM was used to evaluate glycemic parameters including TIR, TAR, TBR, MAGE, LAGE, MPPGE, HbA1c, FPG, PPG, and renal function indicators (eGFR, serum creatinine, BUN) at baseline and study conclusion.

**Results:**

All arms demonstrated significant improvements in TIR, MAGE, LAGE, HbA1c, FPG, and PPG (p<0.001). Arm-A showed a significantly superior reduction in TAR and MPPGE compared to Arm-B (p=0.029 and p=0.040, respectively) and also outperformed Arm-B in reducing FPG (p=0.042). Renal function improved comparably across arms, with a significant decline in serum creatinine noted in Arm-A.

**Conclusion:**

All three FDC therapies significantly improved glycemia, with the Teneligliptin + Dapagliflozin combination offering slightly superior efficacy in reducing TAR, MPPGE, and FPG. These findings support its clinical utility in reducing glycemic variability in patients with T2DM in India. Although favorable trends were observed in renal parameters, the study duration was too short to draw definitive conclusions regarding renal safety. As such, references to renal outcomes should be interpreted with caution, and further long-term studies are warranted to validate these findings.

**Supplementary Information:**

The online version contains supplementary material available at 10.1186/s40842-025-00244-6.

## Introduction

Diabetes mellitus (DM) is a chronic condition marked by high blood glucose levels due to insufficient insulin production or poor utilization. Persistent hyperglycemia poses serious health risks, and fluctuations in blood glucose can also lead to adverse effects [1]. Fluctuations in blood glucose levels over time, whether daily or over longer intervals, are referred to as glycemic variability (GV). While hemoglobin A1c (HbA1c) has been the standard for assessing glycemic control, GV is also gaining recognition as a more meaningful measure in clinical practice, because of its ability to capture fluctuations in blood glucose levels over time [2]. Increased GV is associated with diabetic vascular complications, a higher risk of hypoglycemia, and a decline in quality of life (QOL). Both hyperglycemia and hypoglycemia can worsen GV’s adverse effects through inflammation and oxidative stress [3].

Time in Range (TIR) measures the percentage of time an individual’s glucose levels stay within the target range of 70–180 mg/dL. Current guidelines recommend a TIR goal of over 70% for people with diabetes. Continuous glucose monitoring (CGM) tracks time spent above and below this range, providing insights into hyperglycemia and hypoglycemia, which are crucial for assessing glycemic control [4]. The American Diabetes Association (ADA) recognizes TIR as an important metric linked to the risk of microvascular complications [5]. A recent focus on the South Asian population indicates that a TIR greater than 70% relates to an HbA1c of under 7.5% for Indians [6].

Diabetes is a major cause of chronic kidney disease (CKD) and end-stage kidney disease (ESKD), impacting 30–40% of diabetic individuals. Increased glycemic variability is associated with a higher risk of CKD progression and a more significant decline in estimated glomerular filtration rate (eGFR), suggesting it may be a stronger predictor of kidney disease progression than average baseline HbA1c level [7].

Various interventions have focused on controlling glycemic variability (GV) to mitigate these fluctuations [8]. A rational treatment strategy typically involves combination therapy that targets specific pathophysiology in T2DM through agents that operate via complementary mechanisms [9]. For instance, the simultaneous application of SGLT2-inhibitors and DPP-4 inhibitors addresses multiple facets of the “ominous octet,” leading to safer and more effective glycemic control. Research has shown that combining DPP-4 inhibitors with SGLT2 inhibitors can synergistically reduce glycemic variability [10]. Teneligliptin is effective in lowering glucose levels, reducing the duration spent above the target range, and decreasing glycemic variability, all without an increased risk of hypoglycemia and with a favorable renal profile [11]. Additionally, dapagliflozin, an SGLT2 inhibitor, not only lowers blood glucose levels but also enhances glycemic stability, showing good tolerability in patients with renal impairment [12–16]. Recommendations for South Asian T2DM patients support the combined use of these inhibitors to limit glycemic variability; however, studies using continuous glucose monitoring (CGM) to assess FDCs and GV are still limited [17].

The fixed-dose combination (FDC) of teneligliptin 20 mg and dapagliflozin 10 mg was launched in the Indian market in 2022, marking the inaugural introduction of this combination in the country. Other widely prescribed combinations for Indian patients with type 2 diabetes mellitus (T2DM), particularly those with or at risk of renal complications, include sitagliptin in conjunction with dapagliflozin and empagliflozin combined with linagliptin. This study is the first of its kind aimed at comparing the effects of the FDC of teneligliptin 20 mg and dapagliflozin 10 mg with those of sitagliptin 100 mg plus dapagliflozin 10 mg and the FDC of linagliptin 5 mg combined with empagliflozin 25 mg on glycemic parameters.

## Methodology

### Study details

This is a Prospective, Triple Arm, Randomized, Open Label, Active Controlled Study conducted across 06 sites in India. It was in compliance with the International Council for Harmonization of Technical Requirements for Pharmaceuticals for Human Use-Good Clinical Practice (ICH-GCP). The study was approved by the institutional review board of each site. Informed consent in writing was obtained from all the study participants. (The study was registered on CTRI, CTRI/2023/05/053178).

### Study population

Patients aged 18 years and older with Type 2 Diabetes Mellitus (DM) were assessed for eligibility during their first visit (Day −3 to 0). Inclusion criteria included adults diagnosed with type 2 DM, an HbA1c level between 7.5% and 10%, and stable Metformin monotherapy at a minimum of 1000 mg for over six weeks. Exclusion criteria involved hypersensitivity to study medications, diabetic ketoacidosis, Type 1 DM, cardiac arrhythmias, elevated liver enzyme levels, eGFR below 50 mL/min/1.73 m^2^, acute infections, anemia, pregnancy or lactation, dietary irregularities, and investigator-determined unsuitability

### Study conduct

Baseline demographics including height, weight and BMI, physical examination and vital signs (Blood Pressure, Body temperature, Pulse rate, Respiratory rate) of patients were recorded at all study visits. Metformin was allowed as the background medication during study.

At Visit 2 (Day 1), a CGM device was implanted. On Visit 3 (Day 5), which was five days after the CGM device implantation, the patients were randomized in a 1:1:1 ratio using a computer-generated randomization to receive one of the studies FDCs (FDC Teneligliptin 20 mg + Dapagliflozin 10 mg once daily, FDC Sitagliptin 100 mg + Dapagliflozin 10 mg once daily, FDC Empagliflozin 25mg+ Linagliptin 5 mg once daily). The administration time for all study drugs was consistent, scheduled between 8 AM and 10 AM in the morning, regardless of food intake.

Phase 1 was the timeframe of the first CGM implant in subjects (day 1–day 14). This Phase 1 was further categorized as a pretreatment phase (day 0–day 4) wherein baseline values were recorded. Subjects were initiated on either study product from day 5 onwards, and this period from Day 5 to Day 14 was categorized as the post-treatment period of Phase 1. On day 14, the CGM implant was removed.

Phase 2 of the study: On day 35, a second CGM was implanted in subjects until day 49. This 14-day CGM period (day 35–day 49) was considered Phase 2 of the study. The second CGM was removed on day 49.

End of the study: All subjects enrolled in the study were followed for 90 days. Changes in glycemic and renal parameters (FBS, PPBS, HbA1c, eGFR, S. Creatinine, and UACR) were observed from baseline to day 90. This day, 90, was considered the end of the study.

**Fig. 1:**
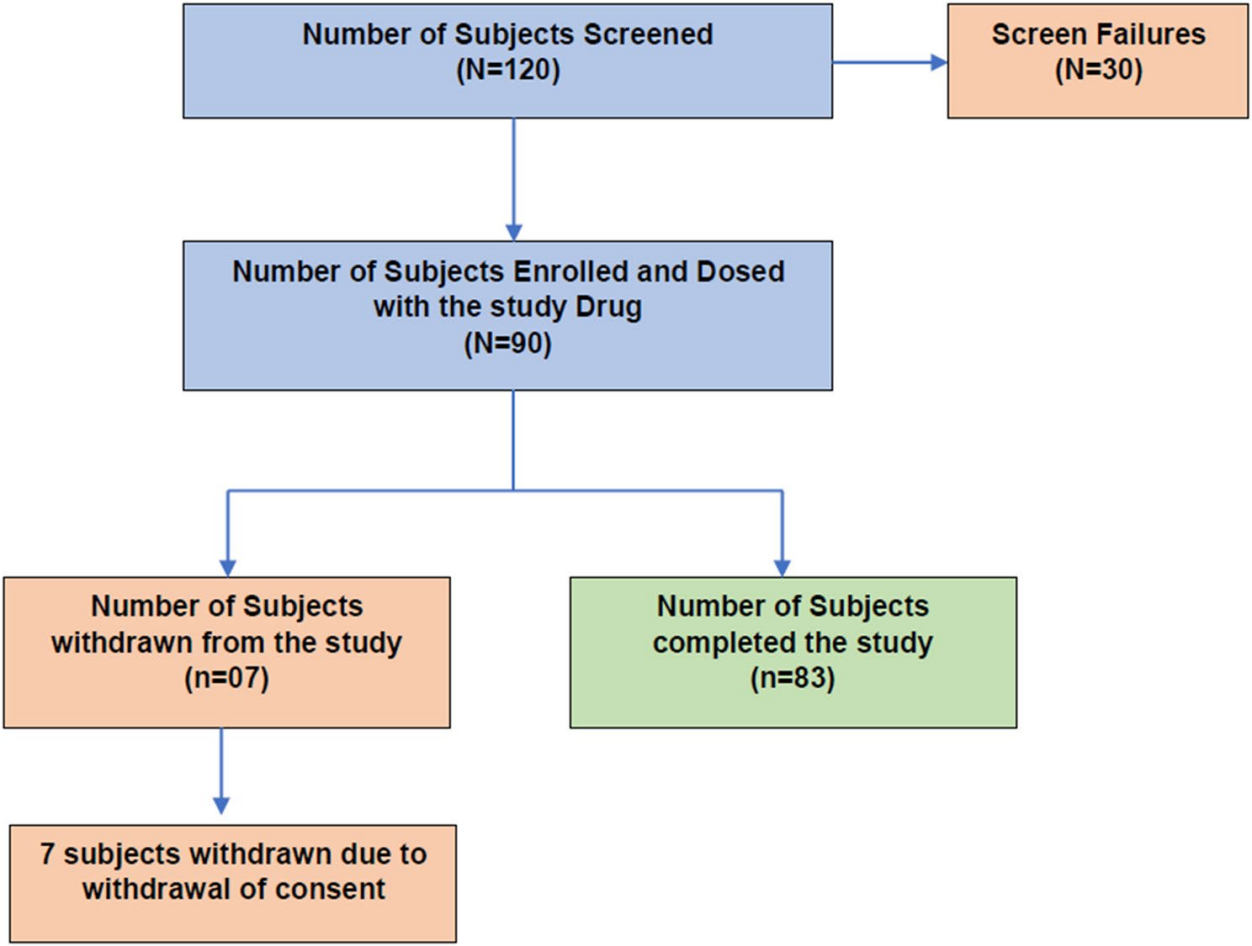
Study flow chart showing patient enrollment, randomization into three treatment arms, and follow-up through study phases

Coefficient of variation of (CV) of 24-h blood glucose, number of events and percentage time at glucose level <70, 70–140 (time-in-target), 70–180(time-in-range), ≥140 mg/dL, ≥180 mg/dL, Mean amplitude of glycemic excursion (MAGE), Largest amplitude of glycemic excursion (LAGE), Mean postprandial glucose excursion (MPPGE), HbA1c, FPG, PPG were determined at baseline, end of phase I and phase II and UACR, eGFR, Sr Creatinine and BUN were determined at baseline and phase II. The Full Analysis Set (FAS) consisted of all enrolled patients with post-baseline assessments, while the Per-Protocol Set (PPS) included those without major protocol deviations. The overall study design, including patient enrollment, randomization, treatment allocation, and follow-up through the two CGM phases, is illustrated in Fig. 1.

### Study intervention

Arm A: FDC of Teneligliptin 20 mg + Dapagliflozin 10 mg OD;

Arm B: FDC of Sitagliptin 100 mg + Dapagliflozin 10 mg OD and

Arm C: FDC of Empagliflozin 25 mg+ Linagliptin 5 mg OD.

### Study endpoints

#### Primary and secondary end-points

The primary endpoint for the study was mean change from baseline in each of parameters of glycemic variability viz, time-in-range [TIR], Time Below Range [TBR], Time Above Range [TAR]) at visit 6 (day 14) and at visit 6 (day 49). (at Immediate post randomization (Visit-3, Day 5 ± 1D), and at visit 6 (day 49). Secondary endpoints included the following: Mean change from baseline to at visit 6 (day 49) and visit 7 (day 90) in HbA1c, FPG and PPG; mean change from baseline in, eGFR, serum creatinine, BUN at visit 7 (day 90) and incidence of any treatment-emergent adverse events in terms of abnormal signs, or laboratory reports.

#### Safety analysis

The safety assessment included analyses of treatment-emergent adverse events, serious adverse events, and laboratory parameters. performance of physical examinations as detailed in the Schedule of Assessments.

#### Statistical analysis

All data will be summarized using descriptive statistics, including the number of subjects, mean, standard deviation (SD), minimum, median, and maximum for continuous variables, and frequency and percentage for categorical variables. At least 75 participants, with 25 complete patients in each arm, were originally considered. Factoring in an anticipated dropout rate of 20%, a total of 90 patients (30 in each group) were ultimately enrolled in the study. For representation purpose, p value between Arm A v/s Arm B, Arm A v/s Arm C and Arm B v/s Arm C will be represented as with the following legends: [a], [b] and [c] respectively.

## Results

### Demographic and baseline characteristics

A total of 90 study participants were enrolled with 30 participants in each of the study arm. In Arm-A, 7 (23.33%) participants were female, 23 (76.67%) participants were male and their mean age was 54.07 ± 10.10 years. In Arm-B, 12 (40.00%) participants were female, and 18 (60.00%) participants were male with a mean age of 54.07 ± 10.60 years. Similarly, in Arm-C, 11 (36.67%) participants were female, 19 (63.33%) participants were male with a mean age of 49.83 ± 11.23 years. P-values were calculated for Table 1a and Table 1b. The analysis demonstrated that there were no statistically significant differences in demographic or clinical characteristics across Arms A, B, and C at baseline, confirming that the study groups were comparable. Baseline demographic and clinical characteristics are given in Table 1a and Table 1b.

**Table 1a:** Baseline demographic characteristics

Parameters	Arm-A (Teneligliptin 20 mg + Dapagliflozin 10 mg)		Arm-B (Sitagliptin 100 mg + Dapagliflozin 10 mg)		Arm-C (Empagliflozin 25 mg + Linagliptin 5 mg)		P-Value
	n	Mean ± SD	n	Mean ± SD	n	Mean ± SD	
**Age (years)**	30	54.07 ± 10.10	30	54.07 ± 10.60	30	49.83 ± 11.23	0.223
**Gender**							
Female	7	23.33%	12	40.00%	11	36.67%	
Male	23	76.67%	18	60.00%	19	63.33%	
**Weight (kg)**	30	71.15 ± 11.14	30	66.25 ± 10.64	30	72.47 ± 12.23	0.180
**Height (cm)**	30	165.08 ± 7.80	30	159.78 ± 13.53	30	164.40 ± 9.38	0.153
**BMI (kg/m**^**2**^)	30	25.98 ± 2.70	30	25.15 ± 3.75	30	26.78 ± 4.19	0.317

**Table 1b:** Baseline clinical characteristics

Parameters	Arm-A (Teneligliptin 20 mg + Dapagliflozin 10 mg)		Arm-B (Sitagliptin 100 mg + Dapagliflozin 10 mg)		Arm-C (Empagliflozin 25 mg + Linagliptin 5 mg)		P-Value
	n	Mean ± SD	n	Mean ± SD	n	Mean ± SD	
Vitals							
Body Temperature (°F)	30	98.08 ± 0.51	30	98.13 ± 0.50	30	98.05 ± 0.62	0.797
Systolic Blood Pressure (mmHg)	30	128.47 ± 6.35	30	127.10 ± 8.21	30	128.90 ± 12.27	0.765
Diastolic Blood Pressure (mmHg)	30	83.10 ± 5.90	30	79.30 ± 8.24	30	82.70 ± 8.42	0.183
Pulse rate (beats/min)	30	80.53 ± 5.69	30	81.03 ± 8.32	30	80.43 ± 10.80	0.965
Glycemic Parameters							
HbA1c (%)	30	8.47 ± 0.72	30	8.44 ± 0.79	30	8.81 ± 0.78	0.109
FPG (mg/dl)	30	144.00 ± 30.57	30	147.28 ± 45.68	30	147.29 ± 38.65	0.967
PPG (mg/dl)	30	194.02 ± 47.17	30	212.14 ± 77.62	30	195.80 ± 44.66	0.509
Renal Parameters							
Estimated Glomerular Filtration Rate (eGFR) (ml/min/1.73m^2^)	30	94.22 ± 16.54	30	98.55 ± 28.18	30	101.05 ± 23.28	0.431
Serum Creatinine (mg/dl)	30	0.90 ± 0.19	30	0.86 ± 0.22	30	0.84 ± 0.25	0.859
Blood urea nitrogen (BUN) (mg/dl)	30	13.68 ± 7.06	30	11.45 ± 2.77	30	14.69 ± 9.26	0.197

### Efficacy parameters

#### Glycemic variability – TIR, TAR and TBR

TIR and TAR showed significant improvement from baseline to end of Phase II for all the 3 study groups (p<0.001), while TBR demonstrated non-significant improvement from baseline to end of study (p>0.05; Table 2). However, at the end of Phase II, improvement in TIR, TBR and TAR across all the three treatment arms A, B and C was comparable (p values; TIR - [a] 0.090 [b] 0.086 [c] 0.959, TBR - [a] 0.053 [b] 0.942 [c] 0.229, TAR - [a] 0.029 [b] 0.357 [c] 0.155 respectively) except for improvement in TAR with Teneligliptin + Dapagliflozin, which was significantly better as compared to Sitagliptin + Dapagliflozin (p = 0.029). TIR improved significantly from 54.5 ± 24.9% at baseline to 78.1 ± 21.5% at the end of Phase II for FDC of Teneligliptin + Dapagliflozin. Likewise, for Sitagliptin + Dapagliflozin and Linagliptin + Empagliflozin, the TIR values improved significantly from 42.7 ± 26.8% to 65.9 ± 28.7% and from 42.3 ± 28.2% to 72.1 ± 24.1% respectively. TAR improved significantly from 40.1 ± 28.1% at baseline to 15.6 ± 21.7% at the end of Phase II for FDC of Teneligliptin + Dapagliflozin. Likewise, for Sitagliptin + Dapagliflozin and Linagliptin + Empagliflozin, the TAR values improved significantly from 54.6 ± 28.9% to 32.0 ± 30.0% and from 55.0 ± 29.7% to 21.3 ± 22.1% respectively.

**Table 2:** Summary of glycemic parameters (average glucose, TIR, TBR, TAR) during different phases of the study with comparison to baseline

Parameters	Visits	Arm-A (Teneligliptin 20 mg + Dapagliflozin 10 mg)			Arm-B (Sitagliptin 100 mg + Dapagliflozin 10 mg)			Arm-C (Empagliflozin 25 mg + Linagliptin 5 mg)			*P*-value (Between arm Comparison)
		*n*	Mean ± SD	*P* Value	*n*	Mean ± SD	*P* Value	*n*	Mean ± SD	*P* Value	
TIR (%)	Baseline	30	54.5 ± 24.9		28	42.7 ± 26.8		29	42.3 ± 28.2		^[a]^ 0.090 ^[b]^ 0.086 ^[c]^ 0.959
	End of phase I	29	68.8 ± 25.6	0.023	28	53.6 ± 34.1	0.063	27	68.2 ± 28.1	<0.001	^[a]^ 0.063 ^[b]^ 0.933 ^[c]^ 0.089
	End of phase II	26	78.1 ± 21.5	<0.001	26	65.9 ± 28.7	0.001	25	72.1 ± 24.1	<0.001	^[a]^ 0.091 ^[b]^ 0.354 ^[c]^ 0.410
TBR (%)	Baseline	30	5.5 ± 8.1		28	2.7 ± 4.0		29	2.6 ± 5.3		^[a]^ 0.103 ^[b]^ 0.114 ^[c]^ 0.939
	End of phase I	29	14.6 ± 23.7	0.061	28	5.1 ± 12.5	0.256	27	5.7 ± 15.8	0.244	^[a]^ 0.063 ^[b]^ 0.103 ^[c]^ 0.870
	End of phase II	26	6.3 ± 9.4	0.427	26	2.1 ± 5.1	0.645	25	6.6 ± 17.6	0.292	^[a]^ 0.053 ^[b]^ 0.942 ^[c]^ 0.229
TAR (%)	Baseline	30	40.1 ± 28.1		28	54.6 ± 28.9		29	55.0 ± 29.7		^[a]^ 0.058 ^[b]^ 0.052 ^[c]^ 0.958
	End of phase I	29	16.6 ± 22.9	<0.001	28	41.3 ± 36.8	0.029	27	25.6 ± 27.6	<0.001	^[a]^ 0.004 ^[b]^ 0.191 ^[c]^ 0.078
	End of phase II	26	15.6 ± 21.7	<0.001	26	32.0 ± 30.0	0.001	25	21.3 ± 22.1	<0.001	^[a]^ 0.029 ^[b]^ 0.357 ^[c]^ 0.155

p-value is calculated using unpaired t-test. ([a]- Arm-A vs Arm-B, [b]- Arm-A vs Arm-C, [c]- Arm-B vs Arm-C)

#### Glycemic variability – MAGE, LAGE and CV

The Mean Amplitude of Glycemic Excursions (MAGE) and Largest Amplitude of Glycemic Excursions (LAGE) showed significant improvement from baseline to end of the study for all the 3 study groups. (p<0.001), while Coefficient of Variability (CV) demonstrated non-significant improvement from baseline to end of study (p>0.05). However, at the end of the study, improvement across all the three-treatment arms A, B and C was comparable, (p > 0.05; MAGE – [a] 0.207 [b] 0.674 [c] 0.372, LAGE - [a] 0.179 [b] 0.990 [c] 0.232, CV - [a] 0.536 [b] 0.333 [c] 0.826 respectively; Table 3). MAGE improved significantly from 80.6 ± 19.2% at baseline to 58.0 ± 13.8% at the end of study for FDC of Teneligliptin + Dapagliflozin. Likewise, for Sitagliptin + Dapagliflozin and Linagliptin + Empagliflozin, MAGE values improved significantly from 86.1 ± 21.3% to 65.5 ± 26.2% and from 87.4 ± 19.0% to 59.9 ± 17.3% respectively.

**Table 3:** Summary of glycemic parameters (MAGE, LAGE, SD, CV) during different phases of the study with comparison to baseline

Parameters	Visits	Arm-A (Teneligliptin 20 mg + Dapagliflozin 10 mg)			Arm-B (Sitagliptin 100 mg + Dapagliflozin 10 mg)			Arm-C (Empagliflozin 25 mg + Linagliptin 5 mg)			P-value (Between arm Comparison)
		n	Mean ± SD	P Value	n	Mean ± SD	P Value	n	Mean ± SD	P Value	
MAGE (mg/dL)	Baseline	30	80.6 ± 19.2		28	86.1 ± 21.3		29	87.4 ± 19.0		^[a]^ 0.386 ^[b]^ 0.179 ^[c]^ 0.672
	End of phase I	29	61.2 ± 21.3	<0.001	28	66.7 ± 20.9	0.001	27	57.4 ± 21.0	<0.001	^[a]^ 0.329 ^[b]^ 0.497 ^[c]^ 0.103
	End of phase II	26	58.0 ± 13.8	<0.001	26	65.5 ± 26.2	<0.001	25	59.9 ± 17.3	<0.001	^[a]^ 0.207 ^[b]^ 0.674 ^[c]^ 0.372
LAGE (mg/dL)	Baseline	30	122.8 ± 31.9		28	132.4 ± 31.7		29	136.3 ± 28.3		^[a]^ 0.313 ^[b]^ 0.091 ^[c]^ 0.510
	End of phase I	29	96.8 ± 30.7	0.003	28	101.2 ± 36.7	<0.001	27	84.9 ± 28.1	<0.001	^[a]^ 0.625 ^[b]^ 0.137 ^[c]^ 0.070
	End of phase II	26	89.5 ± 21.5	<0.001	26	103.3 ± 46.6	<0.001	25	89.6 ± 33.4	<0.001	^[a]^ 0.179 ^[b]^ 0.990 ^[c]^ 0.232
CV (%)	Baseline	30	35.7 ± 10.4		28	33.6 ± 8.4		29	33.1 ± 6.4		^[a]^ 0.336 ^[b]^ 0.242 ^[c]^ 0.905
	End of phase I	29	36.0 ± 10.6	0.989	28	30.0 ± 11.1	0.095	27	28.6 ± 9.3	0.028	^[a]^ 0.042 ^[b]^ 0.008 ^[c]^ 0.626
	End of phase II	26	33.6 ± 8.0	0.44	26	32.1 ± 9.6	0.375	25	31.5 ± 7.0	0.219	^[a]^ 0.536 ^[b]^ 0.333 ^[c]^ 0.826

p-value is calculated using unpaired t-test. ([a]- Arm-A vs Arm-B, [b]- Arm-A vs Arm-C, [c]- Arm-B vs Arm-C)

#### Glycemic variability – MPPGE, PP1 hr and PP2 hr

Mean post-prandial glycemic excursions (MPPGE), Post-prandial levels 1 hr, (PP1 hr) and post-prandial levels 2 hr (PP2 hr) all showed significant improvement from baseline to end of phase II for the study groups A and C (p<0.05) but not for group B (Sitagliptin + Dapagliflozin). However, at the end of the study, improvement across all the three treatment arms A, B and C was comparable (p > 0.05; MPPGE - [a] 0.040 [b] 0.261 [c] 0.245, PP1 hr - [a] 0.588 [b] 0.704 [c] 0.856, PP2 hr - [a] 0.189 [b] 0.716 [c] 0.108 respectively; Table 4) except for the improvement in MPPGE with Teneligliptin + Dapagliflozin, which was significantly superior as compared to Sitagliptin + Dapagliflozin (p = 0.040). MPPGE improved significantly from 159.6 ± 49.6% at baseline to 125.0 ± 33.5% at the end of phase II for FDC of Teneligliptin + Dapagliflozin. Likewise, for Sitagliptin + Dapagliflozin and Linagliptin + Empagliflozin, the MPPGE values improved significantly from 172.3 ± 46.5% to 149.0 ± 46.8% and from 180.3 ± 47.6% to 135.7 ± 33.2% respectively. In a similar way, PP2 hr improved significantly from 160.4 ± 51.7% at baseline to 133.3 ± 43.6% at the end of phase II for FDC of Teneligliptin + Dapagliflozin. Likewise, for Sitagliptin + Dapagliflozin and Linagliptin + Empagliflozin, the PP2 hr values improved significantly from 169.9 ± 47.0% to 155.5 ± 65.3% and from 176.2 ± 49.6% to 128.8 ± 40.4% respectively.

**Table 4:** Summary of post-prandial excursions (MPPGE, PP-1hr, PP-2hr) during different phases of the study with comparison to baseline

Parameters	Visits	Arm-A (Teneligliptin 20 mg + Dapagliflozin 10 mg)			Arm-B (Sitagliptin 100 mg + Dapagliflozin 10 mg)			Arm-C (Empagliflozin 25 mg + Linagliptin 5 mg)			p-value (Between arm Comparison)
		n	Mean ± SD	P Value	n	Mean ± SD	P Value	n	Mean ± SD	P Value	
MPPGE	Baseline	30	159.6 ± 49.6		28	172.3 ± 46.5		29	180.3 ± 47.6		^[a]^ 0.316 ^[b]^ 0.106 ^[c]^ 0.523
	End of phase I	29	135.8 ± 51.2	0.035	28	158.0 ± 53.6	0.182	27	146.2 ± 35.4	0.001	^[a]^ 0.116 ^[b]^ 0.382 ^[c]^ 0.336
	End of phase II	25	125.0 ± 33.5	<0.001	26	149.0 ± 46.8	0.01	25	135.7 ± 33.2	<0.001	^[a]^ 0.040 ^[b]^ 0.261 ^[c]^ 0.245
PP-1hr	Baseline	30	158.0 ± 51.0		28	172.1 ± 44.6		29	176.0 ± 47.7		^[a]^ 0.267 ^[b]^ 0.166 ^[c]^ 0.747
	End of phase I	29	130.8 ± 53.0	0.016	28	152.4 ± 55.2	0.088	25	142.6 ± 37.0	<0.001	^[a]^ 0.138 ^[b]^ 0.342 ^[c]^ 0.447
	End of phase II	25	132.5 ± 40.2	0.021	24	138.8 ± 41.4	0.001	25	136.7 ± 38.7	0.002	^[a]^ 0.588 ^[b]^ 0.704 ^[c]^ 0.856
PP-2hr	Baseline	30	160.4 ± 51.7		28	169.9 ± 47.0		29	176.2 ± 49.6		^[a]^ 0.468 ^[b]^ 0.236 ^[c]^ 0.623
	End of phase I	27	130.3 ± 41.6	0.008	27	161.4 ± 57.2	0.591	23	150.8 ± 44.5	0.036	^[a]^ 0.027 ^[b]^ 0.102 ^[c]^ 0.464
	End of phase II	23	133.3 ± 43.6	0.013	22	155.5 ± 65.3	0.135	24	128.8 ± 40.4	0.001	^[a]^ 0.189 ^[b]^ 0.716 ^[c]^ 0.108

p-value is calculated using unpaired t-test. ([a]- Arm-A vs Arm-B, [b]- Arm-A vs Arm-C, [c]- Arm-B vs Arm-C)

#### Glycemic parameters – HbA1c, FPG and PPG

HbA1c, FPG and PPG showed significant improvement from baseline to end of the study for all the 3 study groups. (p<0.001) However, at the end of the study, improvement across all the three-treatment arms A, B and C was comparable (p values; HbA1c – [a] 0.274 [b] 0.461 [c] 0.742, FPG - [a] 0.042 [b] 0.103 [c] 0.693, PPG - [a] 0.289 [b] 0.661 [c] 0.152 respectively) except for improvement in FPG with Teneligliptin + Dapagliflozin, which was significantly superior as compared to Sitagliptin + Dapagliflozin (p= 0.042; Table 5). HbA1c improved significantly from 8.47 ± 0.72% at baseline to 6.85 ± 0.98% at the end of study for FDC of Teneligliptin + Dapagliflozin. Likewise, for Sitagliptin + Dapagliflozin and Linagliptin + Empagliflozin, the HbA1c values improved significantly from 8.44 ± 0.79% to 7.12 ± 0.90% and from 8.81 ± 0.78% to 7.04 ± 0.92% respectively. Similarly, FPG improved significantly from 144.00 ± 30.57 mg/dl at baseline to 104.18 ± 13.89 mg/dl at the end of the study for FDC of Teneligliptin + Dapagliflozin. Likewise, for Sitagliptin + Dapagliflozin and Linagliptin + Empagliflozin, the FPG values improved significantly from 147.28 ± 45.68 mg/dl to 113.82 ± 20.25 mg/dl and from 147.29 ± 38.65 mg/dl to 111.71 ± 18.54 mg/dl respectively.

**Table 5:** Summary glycemic results (FPG, PPG, HBA1c)

Parameter	Visits	Arm-A (Teneligliptin 20 mg + Dapagliflozin 10 mg)			Arm-B (Sitagliptin 100 mg + Dapagliflozin 10 mg)			Arm-C (Empagliflozin 25 mg + Linagliptin 5 mg)			P-value (Between arm Comparison)
		n	Mean ± SD	P Value	n	Mean ± SD	P Value	n	Mean ± SD	P Value	
HbA1c (%)	Baseline	30	8.47 ± 0.72		30	8.44 ± 0.79		30	8.81 ± 0.78		^[a]^ 0.879 ^[b]^ 0.087 ^[c]^ 0.075
	End of phase II	29	7.14 ± 1.09	<0.001	28	7.85 ± 1.13	0.011	26	7.57 ± 1.21	^<0.001^	^[a]^ 0.019 ^[b]^ 0.175 ^[c] 0.384^
	End of Study	29	6.85 ± 0.98	<0.001	28	7.12 ± 0.90	<0.001	25	7.04 ± 0.92	<0.001	^[a]^ 0.274 ^[b]^ 0.461 ^[c]^ 0.742
FPG (mg/dl)	Baseline	30	144.00 ± 30.57		30	147.28 ± 45.68		30	147.29 ± 38.65		^[a]^ 0.745 ^[b]^ 0.716 ^[c]^ 0.999
	End of phase II	25	118.22 ± 19.46	<0.001	22	123.00 ± 23.96	0.064	19	115.20 ± 14.06	^0.004^	^[a]^ 0.461 ^[b]^ 0.553 ^[c]^ 0.205
	End of Study	29	104.18 ± 13.89	<0.001	28	113.82 ± 20.25	0.001	25	111.71 ± 18.54	<0.001	^[a]^ 0.042 ^[b]^ 0.103 ^[c]^ 0.693
PPG (mg/dl)	Baseline	30	194.02 ± 47.17		30	212.14 ± 77.62		30	195.80 ± 44.66		^[a]^ 0.280 ^[b]^ 0.881 ^[c]^ 0.323
	End of phase II	25	158.12 ± 31.68	0.001	28	165.19 ± 34.68	0.007	26	160.02 ± 40.88	^0.005^	^[a]^ 0.441 ^[b]^ 0.854 ^[c]^ 0.619
	End of Study	29	155.19 ± 40.76	0.003	28	145.06 ± 29.97	<0.001	25	160.30 ± 43.93	0.012	^[a]^ 0.289 ^[b]^ 0.661 ^[c]^ 0.152

p-value is calculated using unpaired t-test. ([a]- Arm-A vs Arm-B, [b]- Arm-A vs Arm-C, [c]- Arm-B vs Arm-C)

### Renal parameters – eGFR, Sr. creatinine and BUN

eGFR, sr. creatinine and BUN showed comparable improvement from baseline to end of the study for all the 3 study groups. (p>0.05) except for significant reduction in sr. creatinine and significant increase in BUN with FDC of Teneligliptin and Dapagliflozin (p<0.024) and FDC of Sitagliptin and Dapagliflozin (p<0.004) respectively. Likewise, at the end of study, improvement across all the three groups A, B and C was comparable (p value; eGFR – [a] 0.983 [b] 0.966 [c] 0.951, sr. creatinine - [a] 0.644 [b] 0.701 [c] 0.992, BUN - [a] 0.222 [b] 0.466 [c] 0.688 respectively; Table 6). eGFR improved significantly from 94.22 ± 16.54 ml/min at baseline to 101.24 ± 20.93 ml/min at the end of study for FDC of Teneligliptin + Dapagliflozin. Likewise, for Sitagliptin + Dapagliflozin and Linagliptin + Empagliflozin, the eGFR values improved from 98.55 ± 28.18 ml/min to 101.37 ± 21.21 ml/min and from 101.05 ± 23.28 ml/min to 100.98 ± 23.70 ml/min respectively. Similarly, Sr. creatinine improved significantly from 0.90 ± 0.19 mg/dl at baseline to 0.83 ± 0.17 mg/dl at the end of study for FDC of Teneligliptin + Dapagliflozin. In case of Sitagliptin + Dapagliflozin and Linagliptin + Empagliflozin, the Sr. creatinine values improved significantly from 0.86 ± 0.22 mg/dl to 0.85 ± 0.16 mg/dl and from 0.84 ± 0.25 mg/dl to 0.85 ± 0.22 mg/dl respectively.

**Table 6:** Summary renal parameters eGFR, serum creatinine, BUN

Parameter	Visits	Arm-A (Teneligliptin 20 mg + Dapagliflozin 10 mg)			Arm-B (Sitagliptin 100 mg + Dapagliflozin 10 mg)			Arm-C (Empagliflozin 25 mg + Linagliptin 5 mg)			P-value (Between arm Comparison)
		n	Mean ± SD	P Value	n	Mean ± SD	P Value	n	Mean ± SD	P Value	
eGFR (ml/min/1.73m^2^)	Baseline	30	94.22 ± 16.54		30	98.55 ± 28.18		30	101.05 ± 23.28		^[a]^ 0.471 ^[b]^ 0.196 ^[c]^ 0.71
	End of study	29	101.24 ± 20.93	0.072	28	101.37 ± 21.21	0.241	25	100.98 ± 23.70	0.943	^[a]^ 0.983 ^[b]^ 0.966 ^[c]^ 0.951
Serum Creatinine (mg/dl)	Baseline	30	0.90 ± 0.19		30	0.86 ± 0.22		30	0.84 ± 0.25		^[a]^ 0.419 ^[b]^ 0.259 ^[c]^ 0.702
	End of study	29	0.83 ± 0.17	0.024	28	0.85 ± 0.16	0.788	25	0.85 ± 0.22	0.819	^[a]^ 0.644 ^[b]^ 0.701 ^[c]^ 0.992
Blood urea nitrogen (BUN)	Baseline	30	13.68 ± 7.06		30	11.45 ± 2.77		30	14.69 ± 9.26		^[a]^ 0.116 ^[b]^ 0.637 ^[c]^ 0.075
	End of study	29	12.83 ± 2.70	0.681	28	13.78 ± 3.05	0.004	25	13.43 ± 3.20	0.916	^[a]^ 0.222 ^[b]^ 0.466 ^[c]^ 0.688

p-value is calculated using unpaired t-test. ([a]- Arm-A vs Arm-B, [b]- Arm-A vs Arm-C, [c]- Arm-B vs Arm-C)

## Discussion

Glycemic variability plays critical role in the overall prognosis of T2DM, which if left unchecked, can act as an independent risk factor for cardiovascular and microvascular events [2]. Therefore, anti-diabetic therapies in addition to good glycemic control, should offer robust benefits in terms of glycemic variability, by maintaining glucose levels within the range of 70–180 mg/dl. SGLT2i + DPP4i provide adequate glycemic control without increased risk of hypoglycemia since their actions are glucose dependent. We evaluated the efficacy of FDC of Teneligliptin + Dapagliflozin in comparison to FDC of Sitagliptin + Dapagliflozin and Linagliptin + Empagliflozin on parameters of GV in T2DM patients using CGM. It was found that parameters of GV and HbA1c, FPG and PPG demonstrated significant improvement from baseline to end of the study in all the treatment arms. TIR, TAR, TBR and glycemic parameters like HbA1c, FPG and PPG, were comparable at the end of study across all the treatment arms.

In a study conducted by Tanaka et al., efficacy of different DPP4 inhibitors (Sitagliptin, Teneligliptin, Vildagliptin and Linagliptin) on GV parameters was evaluated using CGM. DPP4 inhibitors significantly reduced GV from baseline to end of study [18]. In TEDDY study, a randomized, double-blinded, placebo-controlled clinical trial conducted in Korean T2DM patients, effects of teneligliptin on HbA1c levels and GV parameters in elderly T2DM patients were evaluated. Here, the mean change in TIR at week 12 was significant between teneligliptin and placebo group at 13.3 ± 12.0%. (p < 0.001) [11]. Bhattacharyya et al. evaluated efficacy of FDC of Dapagliflozin and Sitagliptin on GV parameters in T2DM patients using CGM. They found a statistically significant improvement in TIR (34.47% increase), and TAR (31.13% decrease) (p<0.05) with the FDC use [19]. The DIVERSITY-CVR study on DPP4i and SGLT2i like Sitagliptin and Dapagliflozin respectively, found that both the molecules individually provided significant improvement in TIR, in T2DM patients with low BMI (Sitagliptin) and high BMI (Dapagliflozin) [20]. Likewise, in our study, all the three FDC’s demonstrated significant improvement in GV parameters from baseline to end of study. In the present study, FDC of Teneligliptin + Dapagliflozin had significant improvement in FPG, MPPGE & TAR compared to Sitagliptin + Dapagliflozin at the end of 3 months. However, a point to note here is the utilization of Sitagliptin and Dapagliflozin in the DIVERSITY-CVR study in two different treatment arms, as opposed to their use as an FDC in our study. Hence, considering the study design of the DIVERISTY-CVR and our study, the generalizability of their findings is limited.

In T2DM patients with renal impairment and CKD, among DPP4 inhibitors no dose adjustment is required for Teneligliptin and linagliptin. Contrary to this, sitagliptin use in T2DM patients with renal impairment and overt CKD mandates dose reduction as per eGFR levels. Teneligliptin has been compared with Linagliptin for glycemic control in T2DM patients with severe renal impairment, with the study concluding both gliptins providing equal benefits in terms of MAGE reduction. Similar results were obtained in our study with FDC of Teneligliptin with Dapagliflozin and Linagliptin with Empagliflozin reducing MAGE from 80.6 ± 19.2% to 58.0 ± 13.8% and from 87.4 ± 19.0 to 59.9 ±17.3% respectively [21]. TOP-RENAL study of Teneligliptin in T2DM patients with CKD demonstrated its efficacy in maintenance of renal parameters (eGFR change of 0.35 ml/min/1.73m^2^ over 6 months) and demonstrated its efficacy in renal impairment [22]. Likewise, linagliptin has been evaluated in T2DM patients with CKD and has been associated with maintenance of renal function [23]. In a study conducted by McGill et al., Linagliptin had little effect on eGFR levels (0.80 ml/min/1.73m^2^ over 12 months) in T2DM patients with severe CKD [24]. These findings are consistent with those from our study, where eGFR was maintained over the duration of study period and was comparable across all the three treatment arms at the end of study.

Availability and use of efficacious anti-diabetic medications play crucial role in holistic management of T2DM. SGLT2i + DPP4i combination therapy occupies an important position in the hierarchy of anti-diabetic agents, owing to the extra-glycemic and additive benefits offered by this FDC. Hence, major diabetes guidelines, ADA, EASD, AACE and others recommend the use of SGLT2i + DPP4i combination for effective diabetes and GV control [25–27]. We compared the recently approved FDC of Teneligliptin + Dapagliflozin to other SGLT2i + DPP4i FDCs approved globally (Linagliptin + Empagliflozin) and in India (Sitagliptin + Dapagliflozin). To the best of our knowledge, this study is one of its kind in comparing FDCs of SGLT2i + DPP4i for evaluation on parameters of GV and renal parameters. Addressing CKD becomes integral when it comes to the management of T2DM. FDC of SGLT2i and DPP4i as evaluated in our study, maintain renal function while optimizing blood glucose levels and GV.

FDC of Teneligliptin and Dapagliflozin along with other FDCs, offer holistic treatment approach for management of T2DM patients with renal impairment. Evaluation of GV parameters by utilization of CGM device with assessment of both glycemic and renal parameters in patients with renal impairment from baseline to end of the study were key strengths of our study. However, smaller sample size, exclusion of T2DM patients suffering from overt CKD and utilization of different SGLT2i in different study arms were the limitations of this study. Other limitations of the study included its open-label, non-blinded design, which may have introduced observer and reporting bias, potentially affecting outcome assessment. The limited duration of the study restricts the ability to comprehensively evaluate long-term outcomes, particularly renal safety, which requires longer follow-up periods for meaningful interpretation.

A noteworthy limitation of this study is the use of different SGLT2 inhibitors across the treatment arms—dapagliflozin in combination with teneligliptin and sitagliptin, and empagliflozin in combination with linagliptin. While this design reflects real-world therapeutic practices in India and enhances the external validity of the findings, it introduces pharmacological heterogeneity that may confound direct comparisons of efficacy across arms.

Although both dapagliflozin and empagliflozin are SGLT2 inhibitors with broadly comparable glycemic efficacy and established cardiovascular and renal benefits, inter-agent variability in pharmacokinetics, SGLT2:SGLT1 selectivity, and tissue distribution may influence individual glycemic profiles, particularly when assessed via continuous glucose monitoring (CGM). These intrinsic differences, albeit subtle, may contribute to variability in glycemic metrics such as time-in-range (TIR), glycemic variability, and postprandial glucose excursions.

This heterogeneity should be considered when interpreting the comparative outcomes, and we recommend future trials aiming for internal consistency employ a uniform SGLT2 inhibitor across all study arms. Nonetheless, the current study offers valuable insights into the comparative performance of these fixed-dose combinations (FDCs) in real-world clinical settings, where therapeutic choices are often guided by local availability, patient affordability, and physician preference.

However, this triple-arm, comparative study included three different SGLT2i and DPP4i combinations and demonstrated the unique role of the FDC of Teneligliptin + Dapagliflozin in the management of T2DM patients by significantly improving TIR and maintaining renal parameters. Therefore, this study establishes the potential role of Teneligliptin + Dapagliflozin combination in terms of GV improvement and preservation of renal function.

## Conclusion

FDCs of Teneligliptin + Dapagliflozin, Sitagliptin + Dapagliflozin, and Linagliptin + Empagliflozin significantly improved glycemic variability in patients with T2DM. The results demonstrated comparable efficacy of the FDC of Teneligliptin + Dapagliflozin with other SGLT2i and DPP4i combinations in ameliorating blood glucose variability, offering clinicians greater flexibility in treatment selection. While favorable trends in renal parameters were observed, the short duration of the study precludes definitive conclusions regarding renal safety. Therefore, references to renal protection should be interpreted with caution, and longer-term studies are necessary to confirm these potential benefits. Despite this limitation, the FDC of Teneligliptin + Dapagliflozin appears to be a promising therapeutic option for Indian patients with T2DM.

## Electronic supplementary material

Below is the link to the electronic supplementary material.

Supplementary Material 1

## Data Availability

No datasets were generated or analysed during the current study.
