# Supplementary material for: Triple arm, prospective, real-world study comparing the efficacy of FDC teneligliptin + dapagliflozin to FDC sitagliptin + dapagliflozin, and FDC linagliptin + empagliflozin in Indian type 2 diabetes mellitus patients using CGM device: the Amplify-TIR study
Source: Cardiovasc Diabetol Endocrinol Rep. 2025 Nov 26;11:32. doi: 10.1186/s40842-025-00244-6 (PMC12648778; doi:10.1186/s40842-025-00244-6)
Supplement: Supplementary file 1 — Supplementary Material 1 [file 40842_2025_244_MOESM1_ESM.pdf]

CLINICAL STUDY REPORT
Protocol No.: NIS/2023/02
Glenmark Pharmaceuticals Ltd.

1. TITLE PAGE

**Study Title**	Triple Arm, Prospective Multicentre, Randomized, Open Label, Active Controlled Study to Assess Effect on FDC of Teneligliptin 20 mg + Dapagliflozin 10 mg compared to FDC of Sitagliptin 100 mg + Dapagliflozin 10 mg and FDC of Linagliptin 5 mg+ Empagliflozin 25 mg on 24 Hour Glucose Profile Assessed By Continuous Glucose Monitoring In Indian Patients of Type 2 Diabetes Mellitus In Real World Setting **(AMPLIFY-TIR STUDY).**
**Short Title**	Study to Assess Effect of FDC of Teneligliptin + Dapagliflozin vs FDC of Sitagliptin + Dapagliflozin vs FDC of Linagliptin + Empagliflozin in Indian Patients of Type 2 Diabetes Mellitus In Real World Setting.
**Study Phase**	Post Marketing Study
**Investigational Product**	Fixed dose combination of Teneligliptin 20 mg + Dapagliflozin 10 mg
**Indication**	Type 2 Diabetes Mellitus.
**Study Sponsor**	Glenmark Pharmaceuticals Ltd.<br>Glenmark Corporate Enclave, BD Sawant Marg, Chakala, Off WE Highway, Andheri E, Mumbai – 400099
**Study Initiation Date**	06-Jun-2023
**Study Completion Date**	05-Jan-2024
**Report Version and Date:**	Version 1.0 dated 02-May-2024

The study including archiving of essential documents was performed in compliance with the Clinical Study Protocol, International Council for Harmonisation (ICH) Good Clinical Practice (GCP), and other applicable regulatory requirements.

CONFIDENTIAL
This document is confidential and the property of Glenmark Pharmaceuticals Ltd.,India. No part of it may be transmitted, reproduced, published, or used by any person/s without prior written authorisation from Glenmark Pharmaceuticals Ltd.

DocUUID : b897a93b-83ba-4de2-a309-31160c3ed1c4

SPONSOR SIGNATURE PAGE

**Study Title:**	Triple Arm, Prospective Multicentre, Randomized, Open Label, Active Controlled Study to Assess Effect on FDC of Teneligliptin 20 mg + Dapagliflozin 10 mg compared to FDC of Sitagliptin 100 mg + Dapagliflozin 10 mg and FDC of Linagliptin 5 mg+ Empagliflozin 25 mg on 24 Hour Glucose Profile Assessed By Continuous Glucose Monitoring In Indian Patients of Type 2 Diabetes Mellitus In Real World Setting (AMPLIFY-TIR STUDY).
**Protocol number**	NIS/2023/02
**Investigational product**	Fixed dose combination (FDC) of Teneligliptin 20 mg + Dapagliflozin 10 mg
**Study Report Version and Date**	Version 1.0 dated 02-May-2024
I have read this report and confirm that, to the best of my knowledge, it accurately describes the conduct and results of the study.	
**<u>Prepared By:</u>**<br>Dr. Pravalika Deti<br>Medical writer<br>Sclin Soft Technologies Private Limited<br>Hyderabad, Telengana – 500017<br>Email: pravalika@sclintech.com	DETI PRAVALIKA<br>Digitally signed by DETI PRAVALIKA<br>DN: c=IN, o=PERSONAL, [illegible]<br>pseudonym=[illegible]<br>2.5.4.20=[illegible]<br>[illegible], postalCode=[illegible], st=Andhra Pradesh,<br>serialNumber=[illegible]<br>[illegible], cn=DETI PRAVALIKA<br>Date: 2024.05.02 17:41:10 +05'30'
**<u>Sponsor's Responsible person</u>**<br>Dr. Sumit Bhushan<br>DGM- Clinical studies- GMA- IF<br>Glenmark Pharmaceuticals Ltd.<br>Glenmark Corporate Enclave, BD Sawant Marg, Chakala, Off WE Highway, Andheri E, Mumbai – 400099<br>Email: Sumit.Bhushan@glenmarkpharma.com	Sumit Bhushan Sumit Bhushan<br>03 May 2024 13:12:40 IST<br>REASON: Reviewed By<br>590a80f6-605b-41c5-ba85-34005ec57b23
**Sponsor's Responsible person:**<br>Dr Sanjay Choudhari<br>General Manager – GMA IF<br>Glenmark Pharmaceuticals Ltd.<br>Glenmark Corporate Enclave, BD Sawant Marg, Chakala, Off WE Highway, Andheri E, Mumbai – 400099<br>E-mail:<br>sanjay.choudhari@glenmarkpharma.com	Sanjay Choudhari Sanjay Choudhari<br>03 May 2024 10:24:55 UTC<br>REASON: Reviewed and Approved By<br>a347e503-56d7-4be1-bc50-eb4de574f790

DocUUID : b897a93b-83ba-4de2-a309-31160c3ed1c4

CRO SIGNATURE PAGE

**Study Title:**	Triple Arm, Prospective Multicentre, Randomized, Open Label, Active Controlled Study to Assess Effect on FDC of Teneligliptin 20 mg + Dapagliflozin 10 mg compared to FDC of Sitagliptin 100 mg + Dapagliflozin 10 mg and FDC of Linagliptin 5 mg+ Empagliflozin 25 mg on 24 Hour Glucose Profile Assessed By Continuous Glucose Monitoring In Indian Patients of Type 2 Diabetes Mellitus In Real World Setting (AMPLIFY-TIR STUDY).
**Protocol number**	NIS/2023/02
**Investigational product**	Fixed dose combination (FDC) of Teneligliptin 20 mg + Dapagliflozin 10 mg
**Study Report Version and Date**	Version 1.0 dated 02-May-2024
I have read this report and confirm that, to the best of my knowledge, it accurately describes the conduct and results of the study.	
Dr. Ashwini Kumar (CEO)<br>CliniExperts Research Services Pvt Ltd.<br>Unit No. 324-325, City Centre Mall, Plot No. 5, Pocket 8, Block B, Sector 12<br>Dwarka, Dwarka, New Delhi, Delhi, 110075<br>Email: ashwini.kumar@cliniexpertsresearch.com	Ashwini Kumar<br>Digitally signed by Ashwini Kumar<br>Date: 2024.05.02 17:40:35 +05'30'
Dr. Veena RM (Head Medical Affairs)<br>CliniExperts Research Services Pvt Ltd.<br>Unit No. 324-325, City Centre Mall, Plot No. 5, Pocket 8, Block B, Sector 12<br>Dwarka, Dwarka, New Delhi, Delhi, 110075<br>Email: veena.rm@cliniexpertsresearch.com	Dr. Veena R M<br>Digitally signed by Dr. Veena R M<br>Date: 2024.05.02 17:44:56 +05'30'
Mr. Prasad Babu (Head of Clinical Operations)<br>CliniExperts Research Services Pvt Ltd.<br>Unit No. 324-325, City Centre Mall, Plot No. 5, Pocket 8, Block B, Sector 12<br>Dwarka, Dwarka, New Delhi, Delhi, 110075<br>Email: prasad.babu@cliniexpertsresearch.com	Prasad Babu M<br>Digitally signed by Prasad Babu M<br>Date: 2024.05.02 18:03:43 +05'30'
Mr. Nitesh Grover (Project Manager)<br>CliniExperts Research Services Pvt Ltd.<br>Unit No. 324-325, City Centre Mall, Plot No. 5, Pocket 8, Block B, Sector 12<br>Dwarka, Dwarka, New Delhi, Delhi, 110075<br>Email:nitesh.g@cliniexpertsresearch.com	Nitesh Grover<br>Digitally signed by Nitesh Grover<br>Date: 2024.05.02 17:41:10 +05'30'
Mr Deepak Prasad (Manager - Clinical Data Management)<br>CliniExperts Research Services Pvt Ltd.<br>Unit No. 324-325, City Centre Mall, Plot No. 5, Pocket 8, Block B, Sector 12<br>Dwarka, Dwarka, New Delhi, Delhi, 110075<br>Email: deepak.prasad@cliniexpertsresearch.com	Deepak Prasad<br>Digitally signed by Deepak Prasad<br>Date: 2024.05.02 17:44:31 +05'30'

DocUUID : b897a93b-83ba-4de2-a309-31160c3ed1c4

INVESTIGATOR SIGNATURE PAGE

**Study Title:**	Triple Arm, Prospective Multicentre, Randomized, Open Label, Active Controlled Study to Assess Effect on FDC of Teneligliptin 20 mg + Dapagliflozin 10 mg compared to FDC of Sitagliptin 100 mg + Dapagliflozin 10 mg and FDC of Linagliptin 5 mg+ Empagliflozin 25 mg on 24 Hour Glucose Profile Assessed By Continuous Glucose Monitoring In Indian Patients of Type 2 Diabetes Mellitus In Real World Setting (AMPLIFY-TIR STUDY).
**Protocol number**	NIS/2023/02
**Investigational product**	Fixed dose combination (FDC) of Teneligliptin 20 mg + Dapagliflozin 10 mg
**Study Report Version and Date**	Version 1.0 dated 02-May-2024
I have read this report and confirm that, to the best of my knowledge, it accurately describes the conduct and results of the study.	
**Principal investigator:**<br>Dr. Suhas Gopal Erande<br>Akshay Hospital, Opp. SNDT College, Karve Rd, near SBI Bank, Pune, Maharashtra 411004	Suhas Erande 8/5/2024<br>Dr. Suhas Erande<br>M.D. (Medicine)<br>Reg. No. MMC - 48919

2. SYNOPSIS

**Title of the study:**	Triple Arm, Prospective Multicentre, Randomized, Open Label, Active Controlled Study to Assess Effect on FDC of Teneligliptin 20 mg + Dapagliflozin 10 mg compared to FDC of Sitagliptin 100 mg + Dapagliflozin 10 mg and FDC of Linagliptin 5 mg+ Empagliflozin 25 mg on 24 Hour Glucose Profile Assessed by Continuous Glucose Monitoring In Indian Patients of Type 2 Diabetes Mellitus In Real World Setting (AMPLIFY-TIR STUDY).
**Name of the finished product:**	Fixed dose combination (FDC) of Teneligliptin 20 mg + Dapagliflozin 10 mg
**Name of the active ingredient:**	Teneligliptin 20 mg + Dapagliflozin 10 mg
**Name of the sponsor:**	Glenmark Pharmaceuticals Ltd.
**Principal investigator(s):**	**Sites in India:**<br>1. Dr. Suhas Gopal Erande, Akshay Hospital, Pune.<br>2. Dr. Namdev Sakharam Jagtap, Spandan Multispeciality Clinic, Pune.<br>3. Dr Mayur Agarwal, Hormone India Diabetes and Endocrine Centre, Bhopal.<br>4. Dr. Sanjeev Gulati, Sarvottam Hospital, Bhopal<br>5. Dr. Praveen Kumar N.S, KR Hospital, Mysore Medical College and Research Institute, Mysuru.<br>6. Dr. Vinod Kumar Kapoor, New Leelamani Hospital, Kanpur
**Study center(s):**	The study was conducted at 06 centers across India.<br>1. Akshay Hospital, Pune<br>2. Spandan Multispeciality Clinic, Pune<br>3. Hormone India Diabetes and Endocrine Centre, Bhopal<br>4. Sarvottam Hospital, Bhopal<br>5. KR Hospital, Mysore Medical College and Research Institute, Mysuru<br>6. New Leelamani Hospital, Kanpur
**Study period:**	The duration of study participation for each subject is maximum of 90 days.
**Phase of development:**	Post Marketing Study
**Date of first enrolment:**	17-Jun-2023
**Date of study completion:**	05-Jan-2024
**Primary objective**	To compare the effect of FDC of Teneligliptin 20 mg + Dapagliflozin 10 mg on 24-hour glucose levels with FDC of Sitagliptin 100 mg + Dapagliflozin 10 mg and FDC of Linagliptin 5 mg + Empagliflozin 25 mg assessed by parameters derived from continuous glucose monitoring in Indian patients of type 2 diabetes mellitus.
**Secondary objective**	• To evaluate Glycaemic parameters of FDC of Teneligliptin 20 mg + Dapagliflozin 10 mg in comparison to FDC of Sitagliptin 100 mg + Dapagliflozin 10 mg and FDC of Linagliptin 5 mg + Empagliflozin 25 mg in Indian patients of type 2 diabetes mellitus

DocUUID : b897a93b-83ba-4de2-a309-31160c3ed1c4

	• To evaluate the safety and tolerability of FDC of Teneligliptin 20 mg + Dapagliflozin 10 mg in comparison to FDC of Sitagliptin 100 mg + Dapagliflozin 10 mg and FDC of Linagliptin 5 mg + Empagliflozin 25 mg in Indian patients of type 2 diabetes mellitus<br>• To assess effect on renal parameters of FDC of Teneligliptin 20 mg + Dapagliflozin 10 mg in comparison to FDC of Sitagliptin 100 mg + Dapagliflozin 10 mg and FDC of Empagliflozin 25 mg + Linagliptin 5 mg in Indian patients of type 2 diabetes mellitus
**Primary endpoint**	• Mean change from baseline in each of parameters of glycaemic variability at post- randomization and at visit 6 (day 49).
**Secondary endpoints:**	• Mean change from baseline in HbA1c, FPG and PPG, at visit 6 (day 49) and visit 7 (day 90)<br>• Mean change from baseline in UACR, eGFR, serum creatinine, BUN at visit 7 (day 90).<br>• Incidence of any treatment emergent adverse events in terms of abnormal signs, or laboratory reports.

Methodology:

This was a prospective, multicenter, randomized, open-label, 3-Arm, active-controlled, comparative study. Patients with T2DM undergoing stable therapy and following a uniform meal pattern were screened and assessed for eligibility during visit 1 (Day -3 to 0) based on inclusion and exclusion criteria. Written informed consent was obtained from all eligible patients before any study-related procedures were conducted.

- Baseline demographics including height, weight and BMI, physical examination and vital signs (Blood Pressure, Body temperature, Pulse rate, Respiratory rate) of patients were recorded at all study visits.
- Blood laboratory assessments and ECG were performed as per schedule of assessments at Visit 1.
- At visit 2 (day 1), all eligible patients are implanted with CGM device and glycemic data was collected until day 5. During the study, Metformin was allowed as background therapy.
- On visit 3 (day 5), i.e. 5 days from the day of implantation of CGM device the patients were randomized in the ratio of 1:1:1 using computer generated randomization list to receive one of study FDCs (FDC Teneligliptin 20 mg + Dapagliflozin 10 mg once daily, FDC Sitagliptin 100 mg + Dapagliflozin 10 mg once daily, FDC Empagliflozin 25mg+ Linagliptin 5 mg once daily) for a period of 90 days.
- Note: The administration time for all study drugs was consistent, scheduled between 8 AM and 10 AM in the morning, regardless of food intake.
- On visit 4 (day 14), which is 14 days after the CGM device was implanted, the patient visited the site for CGM device removal. The period from day of randomization (day 5) to removal of 1st CGM device (day 14), considered as phase I of the study.
- On visit 5 (day 35) 2nd CGM device was implanted, and data was captured until day 49. This 14-day period of CGM device, marked the commencement of phase II in the study.
- At visit 6 (day 49), the patient visited the site for CGM device removal by site personnel/study doctor. Demographic data was recorded, and laboratory assessments were performed.

DocUUID : b897a93b-83ba-4de2-a309-31160c3ed1c4

- Study drugs continued till EOS visit 7 (day 90). Final demographics of patients (height, weight and BMI) was recorded and laboratory assessments were performed. ECG repeated at Visit 7.

The mean of following parameters determined from recording of the CGM devices for period of Day 1 to Day 4 (baseline), Day 5 -14 (Post-randomization) and Day 35-49.

- Mean Blood Glucose
- Standard deviation (SD) Blood glucose
- Coefficient of variation of (CV) of 24-h blood glucose
- Number of events & percentage time at glucose level <70, 70–140 (time-in-target), 70-180 (time-in-range), ≥140 mg/dL, ≥180 mg/dL
- Mean amplitude of glycemic excursion (MAGE)
- Largest amplitude of glycemic excursion (LAGE)
- Mean of daily differences (MODD)
- M value
- Mean postprandial glucose excursion (MPPGE)
- Maximum (Max) and Minimum blood glucose level (Min) before each meal
- Blood glucose at 1 and 2 h after each meal.

Number of subjects (planned and analyzed):

**Number of subjects screened**	120 subjects screened
**No. of subjects planned for inclusion:**	90 Subjects planned for inclusion
**No. of subjects enrolled:**	90 subjects enrolled
**No. of subjects dosed:**	87 subjects dosed
**No. of Subjects completed:**	83 subjects completed the study
**No. of subjects analyzed:**	90 subjects analyzed
**No. of subjects withdrawn/discontinued:**	7 subjects withdrawn due to withdrawal of consent

Diagnosis and main criteria for inclusion:

Inclusion Criteria:

Subjects who met all of the following inclusion criteria were enrolled in the study:

1. Adults (≥ 18 years) of either gender
2. Type 2 DM patients with HbA1c between ≥ 7.5 % to ≤ 10%
3. On stable therapy (>6 weeks prior to screening) of Metformin monotherapy ≥ 1000 mg.
4. Random blood glucose levels <300 mg/dL
5. On complaint dietary regimen for >6 weeks prior to screening
6. Patients who is able to understand & willing to comply with study requirements and provide written informed consent for participation

Exclusion Criteria:

Subjects who met any of the following exclusion criteria were not eligible for participation in the study

1. Hypersensitivity to study medications
2. Presence or evidence of diabetic ketosis or non-ketotic hyperosmolar coma, T1DM

DocUUID : b897a93b-83ba-4de2-a309-31160c3ed1c4

3. Presence of cardiac arrhythmias, acute coronary syndrome
4. Liver enzyme levels > 3 ULN
5. Estimated glomerular filtration rate (eGFR) <50 mL/min/1.73m2
6. Evidence of acute infection
7. Anemia and/or using erythropoiesis stimulating agents
8. Pregnant or lactating women
9. Patient with known dietary irregular patterns
10. A patient who in opinion of investigator would not be suitable for study participation.

Any condition which, in the judgment of the Investigator, may render the patient unable to complete the study or which may pose a significant risk to the patient or patient suspected or with confirmed poor protocol or medication compliance.

Test Product, Dose and Mode of administration, Batch Number
Test Product: FDC Teneligliptin 20 mg + Dapagliflozin 10 mg
Dose: Once daily
Mode of Administration: Oral

Reference Product, Dose and Mode of administration, Batch Number
Reference Product: FDC Sitagliptin 100 mg + Dapagliflozin 10 mg
Dose: Once daily
Mode of Administration: Oral

Reference Product, Dose and Mode of administration, Batch Number
Reference Product: FDC Empagliflozin 25 mg+ Linagliptin 5 mg
Dose: Once daily
Mode of Administration: Oral

Duration of treatment: 90 Days

Statistical method:
The analyses of the primary and secondary endpoints were performed in SAF, FAS & PPS sets.
The Safety Set (SAF) was included all subjects who received at least one dose of the study drug treatment. The safety set used for all safety analyses.
The Full Analysis Set (FAS) was included all enrolled subjects who have received at least one dose of study drug and have at least one post-baseline effectiveness assessment.
The Per-Protocol set (PPS) was included all patients in the FAS who did not have any major protocol deviations.
Safety data during study period were evaluated and summarized descriptively. Adverse events were summarized by system organ class and preferred term. Subjects were counted only once for each preferred term, system organ class, and by the highest severity of an event. Laboratory evaluations were summarized using descriptive statistics at each visit, and change from baseline summarized for each post- baseline visit. Vital signs (temperature, pulse rate, respiratory rate and blood pressure) were summarized descriptively.

Efficacy Results:
- The mean values of CGM parameters like Average Glucose, TIR, TBR, TAR, MAGE, LAGE, SD, CV, MPPGE, PP-1hr, PP-2hr were insignificantly different between the three arms at baseline.
- The mean values of CGM parameters were insignificantly different between the three arms in all parameters except Average Glucose, TAR, CV, post-prandial 1 hr glucose between Arm-A; Arm-B and CV between Arm-A; Arm-C at End of Phase I.
- The mean values of CGM parameters were insignificantly different between the three arms in all parameters except TAR, MPPGE between Arm-A; Arm-B at End of Phase II.
- In Arm-A, comparison of CGM parameters between baseline; End of Phase I showed

DocUUID : b897a93b-83ba-4de2-a309-31160c3ed1c4

statistically significant change in all parameters except TBR and CV.

- In Arm-B, comparison of CGM parameters between baseline; End of Phase I showed statistically significant change in all parameters except TIR, TBR, CV, MPPGE, post-prandial 1 hr glucose and post-prandial 2 hr glucose
- In Arm-C, comparison of CGM parameters between baseline; End of Phase I showed statistically significant change in all parameters except TBR
- In Arm-A and Arm-C, comparison of CGM parameters between baseline; End of Phase II showed statistically significant change in all parameters except TBR and CV.
- In Arm-B, comparison of CGM parameters between baseline; End of Phase II showed statistically significant change in all parameters except TBR, CV, and post-prandial 2 hr glucose
- The comparison of three CGM parameters MPPGE, post-prandial 1hr and 2hr glucose at baseline was insignificantly different between the three arms at breakfast, lunch and dinner.
- The comparison of three CGM parameters MPPGE, post-prandial 1hr and 2hr glucose at End of Phase I was insignificantly different between the three arms at breakfast, lunch and dinner.
- The comparison of three CGM parameters MPPGE, post-prandial 1hr and 2hr glucose at End of Phase II was insignificantly different between the three arms at dinner. However, MPPGE showed significant difference at breakfast between Arm-B; Arm-C and at lunch between Arm-A; Arm-C.
- At breakfast, the comparison of these three parameters MPPGE, post-prandial 1hr and 2hr glucose between baseline; End of Phase I showed statistically significant difference in Arm-A and Arm-C respectively, while none of the parameters showed significant difference in Arm-B.
- At lunch, the comparison of these three parameters MPPGE, post-prandial 1hr and 2hr glucose between baseline; End of Phase I showed no statistically significant difference in Arm-A, Arm-B and Arm-C respectively.
- At dinner, the comparison of these three parameters MPPGE, post-prandial 1hr and 2hr glucose between baseline; End of Phase I showed statistically significant difference only with post-prandial 1hr in Arm-A, while none of the parameters showed significant difference in Arm-B and Arm-C respectively.
- At breakfast, the comparison of these three parameters MPPGE, post-prandial 1hr and 2hr glucose between baseline; End of Phase II showed statistically significant difference in Arm-B and Arm-C respectively, while only MPPGE, post-prandial 2hr showed significant difference in Arm-A.
- At lunch, the comparison of these three parameters MPPGE, post-prandial 1hr and 2hr glucose between baseline; End of Phase II showed statistically significant difference with MPPGE, post-prandial 1hr in Arm-A, while none of the parameters showed significant difference in Arm-B and Arm-C respectively.
- At dinner, the comparison of these three parameters MPPGE, post-prandial 1hr and 2hr glucose between baseline; End of Phase II showed statistically significant difference only with post-prandial 1hr in Arm-A, while none of the parameters showed significant difference in Arm-B and Arm-C respectively.
- The mean HbA1c (%) of the subjects was 8.47 ± 0.72 at baseline which improved to 7.14 ± 1.09 at Day 49 and 6.85 ± 0.98 at Day 90 respectively in Arm-A (Teneligliptin 20 mg + Dapagliflozin 10 mg) subjects, The mean HbA1c (%) of the subjects was 8.44 ± 0.79 at baseline which improved to 7.85 ± 1.13 at Day 49 and 7.12 ± 0.90 at Day 90 respectively in Arm-B (Sitagliptin 100 mg + Dapagliflozin 10 mg) subjects and The mean HbA1c (%) of the subjects was 8.81 ± 0.78 at baseline which improved to 7.57 ± 1.21 at Day 49 and 7.04 ± 0.92 at Day 90 respectively in Arm-C (Empagliflozin 25 mg + Linagliptin 5 mg) subjects
- The mean FPG (mg/dl) was 144.00 ± 30.57 at baseline and improved to 118.22 ± 19.46 at Day 49 and 104.18 ± 13.89 at Day 90 in Arm-A (Teneligliptin 20 mg + Dapagliflozin 10 mg), 147.28

DocUUID : b897a93b-83ba-4de2-a309-31160c3ed1c4

± 45.68 at baseline which improved to 123.00 ± 23.96 at Day 49 and 113.82 ± 20.25 at Day 90 respectively in Arm-B (Sitagliptin 100 mg + Dapagliflozin 10 mg) subjects and The mean FPG (mg/dl) of the subjects was 147.29 ± 38.65 at baseline which improved to 115.20 ± 14.06 at Day 49 and 111.71 ± 18.54 at Day 90 respectively in Arm-C (Empagliflozin 25 mg + Linagliptin 5 mg) subjects

- The mean PPG (mg/dl) was 194.02 ± 47.17 at baseline and improved to 158.12 ± 31.68 at Day 49 and 155.19 ± 40.76 at day 90 in Arm-A (Teneligliptin 20 mg + Dapagliflozin 10 mg), 212.14 ± 77.62 at baseline which improved to 165.19 ± 34.68 at Day 49 and 145.06 ± 29.97 at Day 90 respectively in Arm-B (Sitagliptin 100 mg + Dapagliflozin 10 mg) subjects and The mean PPG (mg/dl) of the subjects was 195.80 ± 44.66 at baseline which improved to 160.02 ± 40.88 at Day 49 and 160.30 ± 43.93 at Day 90 respectively in Arm-C (Empagliflozin 25 mg + Linagliptin 5 mg) subjects.
- Renal parameters of the subject were well maintained throughout the study duration. The Urine Albumin Creatinine Ratio (UACR) of the subjects was 20.13 ± 37.46 at baseline and 41.94 ± 113.06 at Day 90 in Arm-A (Teneligliptin 20 mg + Dapagliflozin 10 mg), 23.18 ± 23.60 at baseline which improved to 41.94 ± 113.06 at Day 90 in Arm-B (Sitagliptin 100 mg + Dapagliflozin 10 mg) subjects and 18.12 ± 13.30 at baseline which improved to 56.53 ± 106.77 at Day 90 in Arm-C (Empagliflozin 25 mg + Linagliptin 5 mg) subjects
- The eGFR of the subjects was 94.22 ± 16.54 at baseline and 101.24 ± 20.93 at day 90 in Arm-A (Teneligliptin 20 mg + Dapagliflozin 10 mg), 98.55 ± 28.18 at baseline which improved to 101.37 ± 21.21 at Day 90 in Arm-B (Sitagliptin 100 mg + Dapagliflozin 10 mg) subjects and 101.05 ± 23.28 at baseline which improved to 100.98 ± 23.70 at Day 90 in Arm-C (Empagliflozin 25 mg + Linagliptin 5 mg) subjects.
- The serum creatinine of the subjects was 0.90 ± 0.19 at baseline and 0.83 ± 0.17 at day 90 in Arm-A (Teneligliptin 20 mg + Dapagliflozin 10 mg), 0.86 ± 0.22 at baseline which improved to 0.85 ± 0.16 at Day 90 in Arm-B (Sitagliptin 100 mg + Dapagliflozin 10 mg) subjects and the 0.84 ± 0.25 at baseline which improved to 0.85 ± 0.22 at Day 90 in Arm-C (Empagliflozin 25 mg + Linagliptin 5 mg) subjects.
- The Blood urea nitrogen (BUN) of the subjects was 13.68 ± 7.06 at baseline and 12.83 ± 2.70 at day 90 in Arm-A (Teneligliptin 20 mg + Dapagliflozin 10 mg), 11.45 ± 2.77 at baseline which improved to 13.78 ± 3.05 at Day 90 in Arm-B (Sitagliptin 100 mg + Dapagliflozin 10 mg) subjects and the mean Blood urea nitrogen (BUN) of the subjects was 14.69 ± 9.26 at baseline which improved to 13.43 ± 3.20 at Day 90 in Arm-C (Empagliflozin 25 mg + Linagliptin 5 mg) subjects.

Safety Results:

A total of 17 adverse events were reported in 10 subjects (11.11%) in the study. Among the 17 adverse events 4 events reported in 3 subjects (10.00%), 6 events reported in 4 subjects (13.33%) and 7 events reported in 3 subjects (3.33%) in Arm-A, Arm-B and Arm-C respectively.

Among the 17 adverse events, 1 event of acidity flatulence in 1 subject (1.11%), 1 event of common cold in 1 subject (1.11%), 1 event of cough in 1 subject (1.11%), 3 events of fever were reported in 3 subjects (3.33%), 1 event of genital infection was reported in 1 subject (1.11%), 1 event of headache in 1 subject (1.11%), 1 event of joint pain in 1 subject (1.11%), 1 event of shivering in 1 subject (1.11%), 1 event of urinary tract infection in 1 subject (1.11%) and 6 events of weakness were reported in 6 subjects (6.67%).

Among the 17 adverse events, the severities of 15 events in 9 (10.0%) subjects were reported as mild and 2 events in 2 (2.22%) subjects were reported as moderate. The relationship of 4 events reported in 3 (3.33%) subjects were related to study drug while for 13 events in 7 (7.78%) subjects were unrelated to Study drug. The outcome of all 17 adverse events reported in 10 subjects (11.11%) were resolved.

DocUUID : b897a93b-83ba-4de2-a309-31160c3ed1c4

No SAEs and deaths were reported in the study.
**Conclusion:**<br>FDC of Teneligliptin + Dapagliflozin was found to be non-inferior and comparable to FDC of Sitagliptin + Dapagliflozin and FDC of Linagliptin + Empagliflozin. All 3 groups showed significant improvement in CGM parameters, HbA1c, FPG, PPG from baseline to end of study and maintained the renal parameters.<br>FDC of Teneligliptin + Dapagliflozin was significantly better than FDC of Sitagliptin + Dapagliflozin in improving MPPGE, FPG and reducing TAR at end of the study.<br>The FDC Teneligliptin + Dapagliflozin offers a promising treatment option to achieve optimal control of Glycemic and CGM parameters. patients with T2DM.
**Date of the report:** 02-May-2024

3. TABLE OF CONTENTS

1. TITLE PAGE 1

2. SYNOPSIS 5

3. TABLE OF CONTENTS 12

4. LIST OF IMPORTANT ABBREVIATIONS AND DEFINITIONS OF TERMS 16

5. ETHICS 17

5.1. Independent Ethics Committee or Institutional Review Board 17

5.2. Ethical Conduct of the Study 19

5.3. Subject Information and Consent 19

5.3.1. Informed consent 19
5.3.2. Subject Data Protection 19

6. INVESTIGATORS AND STUDY ADMINISTRATIVE STRUCTURE 20

6.1. Principal Investigators 20

6.2. Administrative structure 20

7. INTRODUCTION 21

8. STUDY OBJECTIVES AND ENDPOINTS 22

9. INVESTIGATIONAL PLAN 23

9.1. Overall Study Design and Plan: Description 23

9.2. Discussion of the Study Design, Including the Choice of Control Groups 24

9.3. Selection of Study Population 24

9.3.1. Inclusion Criteria 24
9.3.2. Exclusion Criteria 24
9.3.3. Withdrawal Criteria/ Removal of Subjects from the Therapy or Assessment 24

9.4. However, all 83 subjects completed the study and 7 subjects were withdrawn from the studyStudy Interventions 25

9.4.1. Study Interventions Administered 25
9.4.2. Identity of Investigational Products (IP) 25
9.4.3. Method of Assigning Subjects to Study Intervention 25
9.4.4. Selection of Doses in the Study 25
9.4.5. Selection and Timing of Dose for Each Subject 26
9.4.6. Blinding 26
9.4.7. Prior and Concomitant Therapy 26
9.4.8. Treatment Compliance 26

9.5. Study Procedures and Assessments 26

9.5.1. Schedule of Efficacy, Safety Assessments 26
9.5.2. Appropriateness of Measurements 29
9.5.3. Study Variables 29
9.5.4. Efficacy Assessments 29
9.5.5. Safety Assessments 30
9.5.6. Drug Concentration Measurements 31

9.6. Data Quality Assurance 31

9.6.1. Data and Safety Monitoring Board (DSMB) 31
9.6.2. Source Documents 31
9.6.3. Monitoring 32

DocUUID : b897a93b-83ba-4de2-a309-31160c3ed1c4

9.6.4. Investigator and Staff Training 32
9.6.5. Laboratory Procedures 32
9.6.6. Investigator Responsibilities 32
9.6.7. Clinical Data Management 32
9.6.8. Clinical Quality Assurance Audits 33

9.7. Statistical Methods Planned in the Protocol and Determination of Sample Size 33
9.7.1. Statistical and Analytical Plans 33
9.7.2. Analysis Population 33
9.7.3. Interim and Protocol-specified Analyses 33
9.7.4. Primary Endpoint 33
9.7.5. Secondary Endpoint 33
9.7.6. Safety Analyses 33
9.7.7. Determination of Sample Size 34

9.8. Changes in the Conduct of the Study or Planned Analysis 34
9.8.1. Changes in the conduct of the study 34
9.8.2. Changes in the Planned Analyses 34

10. STUDY SUBJECTS 35

10.1. Subject Disposition 35
10.1.1. Handling of Dropouts or Missing Data 35

10.2. Protocol Deviations 36

10.3. Populations Analysed 36

10.4. Demographic and Other Baseline Characteristics 36

10.5. Prior and Concomitant Medications 39

10.6. Study Intervention Exposure and Compliance 41

11. EFFICACY ASSESSMENT 41

11.1. Efficacy Results 58

11.2. Efficacy Conclusions 60

12. SAFETY ASSESSMENT 61

12.1. Extent of Exposure 63

12.2. Adverse events 63
12.2.1. Brief Summary of Adverse Events 63
12.2.2. Display of Adverse Events 63
12.2.3. Analysis of Adverse Events 64
12.2.4. Listing of Adverse Events by Subjects 64

12.3. Deaths, Other Serious and Other Significant Adverse Events 64
12.3.1. Listing of Deaths, Other Serious and Other Significant Adverse Events 64
12.3.2. Narratives of Deaths, Serious Other Significant Adverse Events 65

12.4. Clinical Laboratory Evaluation 65

12.5. Safety Conclusions 65

13. Overall Conclusions 66

14. TABLES AND FIGURES REFERRED IN THE STUDY BUT NOT INCLUDED IN THE TEXT 67

14.1. Demographic Data 67

14.2. Efficacy Data 67

14.3. Safety Data 67

DocUUID : b897a93b-83ba-4de2-a309-31160c3ed1c4

14.3.1. Displays of Adverse Events 67
14.3.2. Listings of Deaths, Other Serious and Significant Adverse Events 67
14.3.3. Narratives of Deaths and Other Serious Adverse Events 67

15. REFERENCES 68

16. APPENDICES....69

16.1. Study Information 69
16.1.1. Protocol and Protocol Amendments 69
16.1.2. Sample Case Report Forms....69
16.1.3. List of IECs/IRBs, and Representative Sample Informed Consent Forms 69
16.1.4. Curriculum Vitae (CV) or Equivalent Summaries of Training and Experience Relevant to the Performance of the Clinical Study 69
16.1.5. Signature Page....69
16.1.6. Listing of Subjects Receiving Investigational Product(s) from Specific Batches, where More Than One Batch was Used....69
16.1.7. Randomisation Scheme and Codes (Subject Identification and Intervention Assigned)....69
16.1.8. Audit Certificates (if available) 69
16.1.9. Documentation of Statistical Method 69
16.1.10. Documentation of Inter-laboratory Standardisation Methods and Quality Assurance Procedures If Used 69
16.1.11. Publications Based on the Study....69
16.1.12. Important Publications Referenced in the Report....69

16.2. Subject Data Listings 69

DocUUID : b897a93b-83ba-4de2-a309-31160c3ed1c4

List of Tables

Table-5 1: IEC/IRB Details and Date of Approvals ... 18

Table-6 1: List of Study Investigators and Site Details ... 20
Table-6 2: Study administrative structure ... 20

Table-9 1: Study Flow Chart ... 28

Table-10 1: Summary of Protocol Deviations Details ... 36
Table-10 2: Key Baseline Demographic Characteristics ... 37
Table-10 3: Summary of baseline characteristics ... 37
Table-10 4: Summary of Vital signs in individual groups visit wise ... 38
Table-10 5: Summary of Concomitant medications ... 40

Table-11 1: Summary of Overall Adverse Events ... 61
Table-11 2: Details of Overall Adverse Events ... 61
Table-11 3: Severity of Overall Adverse Events ... 62
Table-11 4: Relationship of Overall Adverse Events ... 62
Table-11 5: Summary of Action Taken for Adverse Events ... 62
Table-11 6: Outcome of Overall Adverse Events ... 63

Table-11.2 1: Summary of Glycemic parameters (Average Glucose, TIR, TBR, TAR) during different phases of the study with baseline comparison ... 41
Table-11.2 2: Summary of Glycemic parameters (Average Glucose, TIR, TBR, TAR) during different phases of the study comparing between the group ... 42
Table-11.2 3: Summary of Glycemic parameters (MAGE, LAGE, SD, CV) during different phases of the study with baseline comparison ... 44
Table-11.2 4: Summary of Glycemic parameters (MAGE, LAGE, SD, CV) during different phases of the study comparing between the group ... 45
Table-11.2 5: Summary of Post-Prandial Excursions (MPPGE, PP-1hr, PP-2hr) during different phases of the study with baseline comparison ... 46
Table-11.2 6: Summary of Post-Prandial Excursions (MPPGE, PP-1hr, PP-2hr) during different phases of the study comparing between the group ... 47
Table-11.2 7: Summary of Post-Prandial Excursions (MPPGE, PP-1hr, PP-2hr) during different phases of the study with baseline comparison at Breakfast ... 48
Table-11.2 8: Summary of Post-Prandial Excursions (MPPGE, PP-1hr, PP-2hr) during different phases of the study comparing between the group at Breakfast ... 48
Table-11.2 9: Summary of Post-Prandial Excursions (MPPGE, PP-1hr, PP-2hr) during different phases of the study with baseline comparison at Lunch ... 50
Table-11.2 10: Summary of Post-Prandial Excursions (MPPGE, PP-1hr, PP-2hr) during different phases of the study comparing between the group at Lunch ... 51
Table-11.2 11: Summary of Post-Prandial Excursions (MPPGE, PP-1hr, PP-2hr) during different phases of the study with baseline comparison at Dinner ... 52
Table-11.2 12: Summary of Post-Prandial Excursions (MPPGE, PP-1hr, PP-2hr) during different phases of the study comparing between the group at Dinner ... 52
Table-11.2 13: Summary Glycemic results (FPG, PPG, HBA1c) ... 54
Table-11.2 14: Summary Glycemic results (FPG, PPG, HBA1c) with comparison between the group 54
Table-11.2 15: Summary Renal Parameters (UACR, eGFR, Serum Creatinine, BUN) ... 55
Table-11.2 16: Summary Renal Parameters (UACR, eGFR, Serum Creatinine, BUN) with comparison between the group ... 56

DocUUID : b897a93b-83ba-4de2-a309-31160c3ed1c4

4. LIST OF IMPORTANT ABBREVIATIONS AND DEFINITIONS OF TERMS

Abbreviation or Term	Definition/ Explanation
AE	Adverse event
BMI	Body Mass Index
BP	Blood Pressure
CDSCO	Central Drugs Standard Control Organization
CGM	Continuous Glucose Monitoring
CRF	Case Report Form
CRO	Contract Research Organization
CSR	Clinical Study Report
CTRI	Clinical Trials Registry of India
CV	Coefficient of Variation
ECG	Electrocardiogram
eGFR	Estimated Glomerular Filtration rate
FBS	Fasting Blood Sugar
FPG	Fasting Plasma Glucose
GCP	Good Clinical Practice
HbA1c	Glycosylated Hemoglobin
ICF	Informed Consent Form
ICH	International Council for Harmonization
IEC	Independent Ethics Committee
IRB	Institutional Review Board
LAR	Legally Acceptable Representative
MedDRA	Medical Dictionary for Regulatory Activities
PI	Principal Investigator
PP	Per Protocol
PR	Pulse Rate
RFT	Renal Function test
SAE	Serious adverse event
SAP	Statistical Analysis Plan
SOP	Standard Operation Procedure
T2DM	Type 2 Diabetes Mellitus
UACR	Urine Albumin-to-Creatinine Ratio.

DocUUID : b897a93b-83ba-4de2-a309-31160c3ed1c4

5. ETHICS

5.1. Independent Ethics Committee or Institutional Review Board

The study was registered on CTRI (CTRI/2023/05/053178)
A total of 90 subjects were enrolled into the study across 06 sites in India. All 90 subjects were administered with the study drug and were included in the analysis.
The final study protocol, including the final version of the ICF, was approved in writing by an IRB or IEC as appropriate. The Investigator submitted written approval to the Sponsor before enrolment of any subject into the study.
The Investigator was responsible for informing the IRB or IEC of any amendment to the protocol in accordance with local requirements.
The Investigator provided the IRB or IEC with reports of any reportable adverse drug reactions from any other study conducted with the investigational product.
Progress reports and notifications of all adverse drug reactions was provided to the IRB or IEC according to local regulations and guidelines.
The IEC/IRB information and status of study approvals for all sites in India are in Table 5.1 below.

DocUUID : b897a93b-83ba-4de2-a309-31160c3ed1c4

Table-5 1: IEC/IRB Details and Date of Approvals

Site Identifier	Principal Investigator & Qualification	Site Name	IRB/IEC Name and Details	EC Registration Details	Study Approval Details
11	Dr Mayur Agarwal, DM Endocrinologist	Hormone India Diabetes and Endocrine Centre, Bhopal	Institutional Ethics Committee of Charak Hospital and Research Centre,Jahangirabad Square, Pratap Nagar, Jahangirabad, Bhopal, Madhya Pradesh 462008.	ECR/1562/Inst/MP/2021	01-MAY-2023 for Protocol Version: 1.0 Date 03-Mar-2023
12	Dr. Sanjeev Gulati, MD Medicine	Sarvottam Hospital, Bhopal.	Institutional Ethics Committee of Charak Hospital and Research Centre,Jahangirabad Square, Pratap Nagar, Jahangirabad, Bhopal, Madhya Pradesh 462008.	ECR/1562/Inst/MP/2021	01-May-2023 Protocol Version: 1.0 Date 03-Mar-2023
13	Dr.Namdev Sakharam Jagtap, MD Medicine	Spandan Multispeciality Clinic, Pune	Royal Pune Independent Ethics Committee Office No. 13. Srv. No. 81/A, Anupam Arcade, Opposite Snake Park, Katraj, Pune, Maharashtra - 411046, India	ECR/45/Indt/MH/2013/RR-19	11-MAY-2023 for Protocol Version: 1.0 Date 03-Mar-2023
14	Dr. Suhas Gopal Erande, MD Medicine	Akshay Hospital, Pune	Royal Pune Independent Ethics Committee Office No. 13. Srv. No. 81/A, Anupam Arcade, Opposite Snake Park, Katraj, Pune, Maharashtra - 411046, India	ECR/45/Indt/MH/2013/RR-19	11-MAY-2023 for Protocol Version: 1.0 Date 03-Mar-2023
15	Dr. Vinod Kumar Kapoor, MD Medicine	New Leelamani Hospital, Kanpur	Institutional Ethics Committee-Leelamani Hospital<br>14/116, C-1, Parade Chauraha, Civil Lines, Kanpur, Uttar Pradesh, 208001	ECR/1696/Inst/UP/2022	25-APR-2023 for Protocol Version: 1.0 Date 03-Mar-2023
16	Dr. Praveen Kumar N.S, DM Endocrinologist	K R Hospital, Mysore medical College and Research Institute, Mysuru	Institutional Ethics Committee- Mysore medical College and Research Institute Irwin Road, next to Railway Station, Mysuru, Karnataka 570001	ECR/134/Inst/KA/2013/RR-19	16-AUG-2023 for Protocol Version: 1.0 Date 03-Mar-2023

DocUUID : b897a93b-83ba-4de2-a309-31160c3ed1c4

5.2. Ethical Conduct of the Study

This study was conducted in accordance with the study protocol, the New Drugs and Clinical Trials Rules 2019 issued by the Government of India, the ethical principles that have their origin in the Declaration of Helsinki (64th WMA General Assembly, Fortaleza, Brazil, October 2013), the International Council for Harmonisation (ICH) Good Clinical Practice (GCP), and all applicable local regulatory requirements. The investigators agreed to conduct the study according to the principles of the ICH GCP, and in accordance with the ethical principles that have their origin in the Declaration of Helsinki (64th WMA General Assembly, Fortaleza, Brazil, October 2013), the protocol, and all national, state, and local laws or regulations. The medical care given to, and a medical decision made on behalf of study subjects was always the responsibility of a Principal (Site) Investigator (PI). Each individual involved in conducting the study was qualified by education, training, and experience to perform his or her respective task(s).

5.3. Subject Information and Consent

5.3.1. Informed consent

Written informed consent was obtained from each subject before entering into the study and before any study-specific procedures were performed. The informed consent form (ICF) was submitted by the investigator to the IEC/IRB for review and approval before the start of the study. Sample informed consent forms are included as Appendix 16.1.3 of this report. Subjects and/or their legally acceptable representative (LAR) were informed that their participation was voluntary. Consent was documented by the subject’s dated signature (or left thumb impression) along with the dated signature of responsible study personnel. The investigator or his/her representative explained the nature of the study to the subject or his/her legally authorised representative and answered all questions regarding the study. In accordance with ICH GCP guidelines, the subject’s LAR signed for the subject and was given ample opportunity to ask about details of the study. If the subject was illiterate, an impartial witness was present during the entire informed consent process and also signed and dated the consent form. Investigative sites were instructed to obtain written informed consent before a subject was enrolled in the study and document the date the written consent was obtained. The authorised person obtaining the informed consent was also instructed to sign the ICF. A copy of the signed and dated ICF was provided to the subject prior to his/her participation in the study.

5.3.2. Subject Data Protection

Subject confidentiality was maintained throughout the study. All reports, CRF, and communications relating to subjects in the study were identified only by the subject number and the protocol number.

DocUUID : b897a93b-83ba-4de2-a309-31160c3ed1c4

6. INVESTIGATORS AND STUDY ADMINISTRATIVE STRUCTURE

6.1. Principal Investigators

The study was conducted in 06 sites across India. The details of the Principal Investigators (PI) responsible for the overall conduct of the clinical trial at their respective sites are presented in Table 6.1 below. The Curriculum Vitae (CV) of the PIs are provided as Appendix 16.1.4 to this report.

Table-6 1: List of Study Investigators and Site Details

S.No	Site Number	Principal Investigator Name & Qualifications	Site Name and Details
1	11	Dr Mayur Agarwal, DM Endocrinologist	Hormone India Diabetes and Endocrine Centre, Bhopal
2	12	Dr. Sanjeev Gulati, MD Medicine	Sarvottam Hospital, Bhopal
3	13	Dr. Namdev Sakharam Jagtap, MD Medicine	Spandan Multispeciality Clinic, Pune
4	14	Dr. Suhas Gopal Erande, MD Medicine	Akshay Hospital, Pune
5	15	Dr. Vinod Kumar Kapoor, MD Medicine	New Leelamani Hospital, Kanpur
6	16	Dr. Praveen Kumar N.S, DM Endocrinologist	KR Hospital, Mysore Medical College and Research Institute, Mysuru

6.2. Administrative structure

A list of organisations that are critical to the conduct of this study are presented in Table 6.2 below:

Table-6 2: Study administrative structure

**Sponsor's Medical Monitor**	Dr. Sumit Bhushan<br>DGM- Clinical studies- GMA- IF<br>Glenmark Pharmaceuticals Ltd.<br>Glenmark Corporate Enclave, BD Sawant Marg, Chakala, Off WE Highway, Andheri E, Mumbai – 400099<br>Email: Sumit.Bhushan@glenmarkpharma.com
**Sponsor's Clinical Project Manager**	Rujuta Gadkari<br>Senior Manager – GMA IF<br>Glenmark Pharmaceuticals Ltd.<br>Glenmark Corporate Enclave, BD Sawant Marg, Chakala, Off WE Highway, Andheri E, Mumbai – 400099<br>E-mail: rujuta.gadkari@glenmarkpharma.com

DocUUID : b897a93b-83ba-4de2-a309-31160c3ed1c4

7. INTRODUCTION

The global prevalence of diabetes mellitus is increasing rapidly due to population growth, aging, urbanization and change in life style. India is witnessing exponential growth in number of people with diabetes: there were approximately 72.9 million people with diabetes in 2017 which is expected to increase to 134.4 million by 2045[1].

Medical management of patients with type 2 diabetes mellitus (T2DM) consists of diet, exercise, and weight reduction, together with insulin or oral or injectable anti-diabetic medications that increase insulin secretion (sulphonlyureas [SUs] and meglitinides); improve insulin sensitivity and increase glucose utilization (glitazones); reduce hepatic glucose production (metformin); delay glucose absorption (α-glucosidase inhibitors); enhancing the levels of active incretin hormones by inhibiting dipeptidyl peptidyl IV (DPP-IV; sitagliptin); enhance glucose dependent insulin secretion through glucagon-like peptide (exenatide).[2]

Approximately two-thirds of patients with T2DM do not effectively control their glucose levels in developed countries and this proportion is even greater in developing countries. Given the huge burden of T2DM on public health system, there is an urgent need for prompt intervention to improve the glycemic control so as to avert its short and long-term complications. This also calls for focus on new parameters like glycaemic variability (GV) in addition to traditional end glucose levels and glycated haemoglobin (HbA1C).[3] There has been recent recognition of the limitations of HbA1c in describing both short- and long-term glycemic control. Continuous glucose monitoring (CGM) provides the ability to measure and observe inter- and intraday GV, which is a more meaningful measure of glycaemic control. CGM provides robust data about short-term glycemic control and provides metrics such as percent time-in-range (%TIR) that are now routinely reported to describe the change in glycemic control after an intervention in a clinical study or a change in therapy in a patient's care. Recent studies have shown that %TIR may have similar associations with diabetes microvascular complications as does HbA1c.[4]

Studies with SGLT2i agents as well as DPP4i agents suggest that these drugs have beneficial effects on TIR. Systematic reviews and meta-analyses of 16 randomized controlled trials (RCTs) with SGLT2i and seven RCTs of DPP4i have demonstrated that these agents reduce glycemic variability in patients with T2DM. The Time in Range recommendations for South Asia suggests frequency for repeating TIR evaluation, which may be minimal for therapies such as SGLT2i and DPP4i with minimal glycaemic variability again reducing the cost and complications.[5]

There is a lack of TIR data for DPP 4 inhibitor + SGLT 2 inhibitor. Teneligliptin + Dapagliflozin is a novel DPP 4 inhibitor + SGLT2 inhibitor FDC which has been shown to provide equivalent glycemic control in Phase III study. However, the impact on daily glucose levels is unknown.

Also, considering renal function status, mainly Teneligliptin + Dapagliflozin and Empagliflozin + Linagliptin are prescribed in Indian T2DM patients with renal diseases. However, there is no head to head comparative trial for renal evaluation of these 2 combinations.

Hence, this study is being conducted to evaluate effect of FDC Teneligliptin + Dapagliflozin on 24-hour glucose profile assessed by continuous glucose monitoring and also to assess renal parameters in Indian patients of type 2 diabetes mellitus as compared to FDC Sitagliptin + Dapagliflozin and FDC Empagliflozin + Linagliptin.

DocUUID : b897a93b-83ba-4de2-a309-31160c3ed1c4

8. STUDY OBJECTIVES AND ENDPOINTS

Primary Objective	Secondary Objective:
The primary objective of the study is to compare the effect of FDC of Teneligliptin 20 mg + Dapagliflozin 10 mg on 24-hour glucose levels with FDC of Sitagliptin 100 mg + Dapagliflozin 10 mg and FDC of Linagliptin 5 mg + Empagliflozin 25 mg assessed by parameters derived from continuous glucose monitoring in Indian patients of type 2 diabetes mellitus.	The secondary objective of the study is<br>• To evaluate Glycaemic parameters of FDC of Teneligliptin 20 mg + Dapagliflozin 10 mg in comparison to FDC of Sitagliptin 100 mg + Dapagliflozin 10 mg and FDC of Linagliptin 5 mg + Empagliflozin 25 mg in Indian patients of type 2 diabetes mellitus<br>• To evaluate the safety and tolerability of FDC of Teneligliptin 20 mg + Dapagliflozin 10 mg in comparison to FDC of Sitagliptin 100 mg + Dapagliflozin 10 mg and FDC of Linagliptin 5 mg + Empagliflozin 25 mg in Indian patients of type 2 diabetes mellitus<br>• To assess renal parameters of FDC of Teneligliptin 20 mg + Dapagliflozin 10 mg in comparison to FDC of Sitagliptin 100 mg + Dapagliflozin 10 mg and FDC of Linagliptin 5 mg + Empagliflozin 25 mg in Indian patients of type 2 diabetes mellitus.
**Primary endpoint**	**Efficacy endpoints:**
• Mean change from baseline in each of parameters of glycemic variability at post randomization and at visit 6 (day 49).	• Mean change from baseline in HbA1c, FPG and PPG, at visit 6 (day 49) and visit 7 (day 90)<br>• Mean change from baseline in UACR, eGFR, serum creatinine, BUN at visit 7 (day 90).<br>• Incidence of any treatment emergent adverse events in terms of abnormal signs, or laboratory reports.

Safety parameters

Safety assessments consisted of monitoring and recording all AEs and SAEs; regular monitoring of hematology and blood chemistry; periodic measurement of vital signs and ECG; and performance of physical examinations as detailed in the Schedule of Assessments (Table 9.1).

In case of premature discontinuation, the reason and their cause were documented. All appropriate assessments applicable for Day 90 visit, were planned to be conducted at the early withdrawal visit following the last dose of study medication. If the withdrawal was due to an AE, the AE was monitored until it was resolved or it has returned to a status that was prior to the AE. However, no dropouts were reported during the study.

The safety assessment included analyses of treatment emergent AEs, SAE, Treatment emergent adverse events, laboratory parameters.

DocUUID : b897a93b-83ba-4de2-a309-31160c3ed1c4

9. INVESTIGATIONAL PLAN

9.1. Overall Study Design and Plan: Description

This is a prospective, multicenter, randomized, open-label, 3-Arm, active-controlled, comparative study. Patients with T2DM undergoing stable therapy and following a uniform meal pattern were screened and assessed for eligibility during visit 1 (Day -3 to 0) based on inclusion and exclusion criteria. Written informed consent was obtained from all eligible patients before any study-related procedures were conducted.

- Baseline demographics including height, weight and BMI, physical examination and vital signs (BP, Body temperature, Pulse rate, Respiratory rate) of patients were recorded at all study visits.
- Blood laboratory assessments and ECG were performed as per schedule of assessments at Visit 1.
- At visit 2 (day 1), all eligible patients are implanted with CGM device and glycemic data was collected until day 5. During the study, Metformin was allowed as background therapy.
- On visit 3 (day 5), i.e. 5 days from the day of implantation of CGM device the patients were randomized in the ratio of 1:1:1 using computer generated randomization list to receive one of study FDCs (FDC Teneligliptin 20 mg + Dapagliflozin 10 mg once daily, FDC Sitagliptin 100 mg + Dapagliflozin 10 mg once daily, FDC Empagliflozin 25mg+ Linagliptin 5 mg once daily) for a period of 90 days.

  Note: the administration time for all study drugs was consistent, scheduled between 8 AM and 10 AM in the morning, regardless of food intake.
- On visit 4 (day 14), which is 14 days after the CGM device was implanted, the patient visited the site for CGM device removal. The period from day of randomization (day 5) to removal of 1st CGM device (day 14), considered as phase I of the study.
- On visit 5 (day 35) 2nd CGM device was implanted, and data was captured until day 49. This 14-day period of CGM device, marked the commencement of phase II in the study.
- At visit 6 (day 49), the patient visited the site for CGM device removal by site personnel/study doctor. Demographic data was recorded, and laboratory assessments were performed.
- Study drugs continued till EOS visit 7 (day 90). Final demographics of patients (height, weight and BMI) was recorded and laboratory assessments were performed. ECG repeated at Visit 7.

The mean of following parameters determined from recording of the CGM devices for period of Day 1 to Day 4 (baseline), Day 5 -14 (Post-randomization) and Day 35-49.

- Mean Blood Glucose
- Standard deviation (SD) Blood glucose
- Coefficient of variation of (CV) of 24-h blood glucose
- Number of events & percentage time at glucose level <70, 70–140 (time-in-target), 70-180 (time-in-range), ≥140 mg/dL, ≥180 mg/dL
- Mean amplitude of glycemic excursion (MAGE)
- Largest amplitude of glycemic excursion (LAGE)
- Mean of daily differences (MODD)
- M value
- Mean postprandial glucose excursion (MPPGE)
- Maximum (Max) and Minimum blood glucose level (Min) before each meal
- Blood glucose at 1 and 2 h after each meal.

DocUUID : b897a93b-83ba-4de2-a309-31160c3ed1c4

9.2. Discussion of the Study Design, Including the Choice of Control Groups

This is a Prospective, Triple Arm, Multicentre, Randomized, Open Label, Active Controlled Study to Assess Effect on FDC of Teneligliptin 20 mg + Dapagliflozin 10 mg compared to FDC of Sitagliptin 100 mg + Dapagliflozin 10 mg and FDC of Linagliptin 5 mg+ Empagliflozin 25 mg on 24 Hour Glucose Profile Assessed by Continuous Glucose Monitoring in Indian Patients of Type 2 Diabetes Mellitus In Real World Setting **(AMPLIFY-TIR STUDY).**

A total of 120 subjects were screened, among which 90 subjects were enrolled into the study and 30 subjects were screen failures.

A total of seven visits were scheduled for each participant in the study [i.e., Screening Visit: Baseline Visit 1 (Day -3 to 0); Enrolment Visit 2 (Day 1), Randomization Visit (Visit-3) (Day 5 ± 1D), Physical Visit-4 (Day 14 ± 1D), Physical Visit (Visit-5) (Day 35 ± 2D), Physical Visit (Visit-6) (Day 49 ± 2D) End of Study Visit: Physical Visit (Visit-7) (D ay 90 ± 2D). The total duration of the study is a maximum of 93 days (3 days for screening + 90 days of treatment)

9.3. Selection of Study Population

9.3.1. Inclusion Criteria

Subjects who met all of the following inclusion criteria were enrolled in the study:

1. Adults (≥ 18 years) of either gender
2. Type 2 DM patients with HbA1c between ≥ 7.5 % to ≤ 10%
3. On stable therapy (>6 weeks prior to screening) of Metformin monotherapy ≥ 1000 mg
4. Random blood glucose levels <300 mg/dL
5. On complaint dietary regimen for >6 weeks prior to screening.
6. Patients who is able to understand & willing to comply with study requirements and provide written informed consent for participation.

9.3.2. Exclusion Criteria

Subjects who met any of the following exclusion criteria were not eligible for participation in the study:

1. Hypersensitivity to study medications
2. Presence or evidence of diabetic ketosis or non-ketotic hyperosmolar coma, T1DM
3. Presence of cardiac arrhythmias, acute coronary syndrome
4. Liver enzyme levels > 3 ULN
5. Estimated glomerular filtration rate (eGFR) <50 mL/min/1.73m2
6. Evidence of acute infection
7. Anemia and/or using erythropoiesis stimulating agents
8. Pregnant or lactating women
9. Patient with known dietary irregular patterns
10. A patient who in opinion of investigator would not be suitable for study participation.

Any condition which, in the judgment of the Investigator, may render the patient unable to complete the study or which may pose a significant risk to the patient or patient suspected or with confirmed poor protocol or medication compliance.

9.3.3. Withdrawal Criteria/ Removal of Subjects from the Therapy or Assessment

Any subject was allowed to voluntarily discontinue from the study at any time after giving informed consent and before the completion of the last visit of the study.

Subjects may also be withdrawn from study drug treatment at the discretion of the Investigator or

DocUUID : b897a93b-83ba-4de2-a309-31160c3ed1c4

Sponsor for safety, noncompliance, or administrative reasons. The Investigator may also discontinue the subject's study participation at any time at his/her discretion and for any reason.

The reasons for subject withdrawal were recorded and included, but are not limited to:

1. Withdrawal of consent by the subject to continue in the study. Once the subject withdrew consent, it was planned that the subject will not receive any further investigational product or further study observation. The subject also needed to undergo additional tests or tapering of treatment to withdraw safely.
2. Development of a serious or intolerable adverse event (AE) that necessitates discontinuation at the discretion of the Investigator (the AE section of the CRF must be completed; AE includes serious adverse event (SAE) and death.
3. At the discretion of the Investigator, when he/she believes continued participation is not in the best interest of the subject.
4. At the discretion of the Investigator, when the subject does not adhere to the study procedures.
5. A protocol deviation that, in the opinion of the Sponsor and Investigator, warrants discontinuation from the study.
6. Positive Pregnancy test at any of the study visits.

9.4. However, all 83 subjects completed the study and 7 subjects were withdrawn from the studyStudy Interventions

9.4.1. Study Interventions Administered

Fixed dose combination (FDC) of Teneligliptin 20 mg + Dapagliflozin 10 mg.
Fixed dose combination (FDC) of Sitagliptin 100 mg + Dapagliflozin 10 mg
Fixed dose combination (FDC) of Empagliflozin 25 mg+ Linagliptin 5 mg

9.4.2. Identity of Investigational Products (IP)

Fixed dose combination (FDC) of Teneligliptin 20 mg + Dapagliflozin 10 mg Tablets, Fixed dose combination (FDC) of Sitagliptin 100 mg + Dapagliflozin 10 mg and Fixed dose combination (FDC) of Empagliflozin 25 mg+ Linagliptin 5 mg tablets orally administered once daily.

9.4.3. Method of Assigning Subjects to Study Intervention

90 male and female subjects aged ≥18 years, diagnosed with Type 2 DM patients with HbA1c between ≥ 7.5 % to ≤ 10% were enrolled in the study.

9.4.4. Selection of Doses in the Study

SGLT2i such as dapagliflozin, by specifically targeting the kidney, inhibit glucose reabsorption at the proximal tubule and thereby promote glucosuria, an effect independent of insulin. Because of the progressive deterioration of beta-cell function that characterizes T2DM, a pharmacological mechanism of action that is independent of pancreatic beta-cell function makes SGLT2i an appropriate option for patients with advanced T2DM, particularly if their glycaemic control is inadequate with other oral glucose-lowering agents. By promoting glucosuria and reducing hyperglycaemia, SGLT2i dampen glucotoxicity, which indirectly results in an improvement of beta-cell function and peripheral insulin sensitivity. However, treatment with SGLT2i resulted in an increase in plasma glucagon concentrations, which was accompanied by a substantial increase in

DocUUID : b897a93b-83ba-4de2-a309-31160c3ed1c4

endogenous (hepatic) glucose production. The latter has been estimated to offset approximately half of the glucose excreted in the urine as a result of SGLT2i. DPP-4i such as teneligliptin enhance postprandial insulin secretion and suppress glucagon secretion by preventing the degradation of endogenously released incretin hormones [glucagon like peptide (GLP)-1 and glucose-dependent insulinotropic polypeptide (GIP)], two intestinal peptides whose concentrations physiologically increase after food intake. of major interest, DPP 4 inhibitors stimulate insulin secretion and inhibit glucagon secretion in a glucose-dependent manner, thus reducing hyperglycaemia while minimizing hypoglycaemia. Thus, the addition of a teneligliptin which inhibits glucagon and stimulates insulin secretion may have the potential to block the increase in endogenous glucose production and enhance the glucose-lowering ability of dapagliflozin. Taken together these findings suggest that the combination of teneligliptin with dapagliflozin would potentially provide additional help to individuals with T2D in reaching their glycaemic goal.

9.4.5. Selection and Timing of Dose for Each Subject

Arm A: FDC of Teneligliptin 20 mg + Dapagliflozin 10 mg OD
Arm B: FDC of Sitagliptin 100 mg + Dapagliflozin 10 mg OD
Arm C: FDC of Empagliflozin 25 mg+ Linagliptin 5 mg OD

9.4.6. Blinding

This was an open label study. Hence, blinding procedure is not applicable.

9.4.7. Prior and Concomitant Therapy

All the Prior and concomitant medications were recorded in the Prior and concomitant medications form of the CRF

9.4.8. Treatment Compliance

Investigational product was prescribed by the investigator and the IP dispensed to the subjects by site team. Subjects were asked to bring used/unused strips of drug at next visit, study site personnel monitor the compliance.

9.5. Study Procedures and Assessments

9.5.1. Schedule of Efficacy, Safety Assessments

Efficacy assessment parameters are as below-

1. Assessment of glycemic variability at Immediate post randomization and at Visit 6 (Day 49) using a CGM device. Following CGM parameters will be evaluated-
    - Mean Blood Glucose
    - Standard deviation (SD) Blood glucose
    - Coefficient of variation of (CV) of 24-h blood glucose
    - Number of events & percentage time at glucose level <70, 70–140 (time-in-target), 70-180 (time-in-range), ≥140 mg/dL, ≥180 mg/dL
    - Mean amplitude of glycemic excursion (MAGE)
    - Largest amplitude of glycemic excursion (LAGE)
    - Mean of daily differences (MODD)
    - M value
    - Mean postprandial glucose excursion (MPPGE)
    - Maximum (Max) and Minimum blood glucose level (Min) before each meal

DocUUID : b897a93b-83ba-4de2-a309-31160c3ed1c4

- Blood glucose at 1 and 2 h after each meal

2. HbA1c, FPG, PPG evaluation at baseline, Day 49 and Day 90
3. UACR, eGFR, Sr Creatinine and BUN assessment at baseline and Day 90

Safety assessments consisted of monitoring and recording all AEs and SAEs; regular monitoring of hematology and blood chemistry; periodic measurement of vital signs and ECG; and performance of physical examinations as detailed in the Schedule of Assessments.
In case of premature discontinuation, the reason and their cause were documented. In instances where withdrawal is a result of an adverse event (AE), monitoring of the AE continues until it is resolved or returns to its pre-event status.

DocUUID : b897a93b-83ba-4de2-a309-31160c3ed1c4

Table-9 1: Study Flow Chart

Procedure	Visit 1	Visit 2	Visit 3	Visit 4	Visit 5	Visit 6	Visit 7
**Day / Week**	**Day -3 to 0**	**Day 1**	**Day 5 (± 1D)**	**Day 14 (± 1D)**	**Day 35 (± 2D)**	**Day 49 (± 2D)**	**Day 90 (± 2D)**
**Follow-up**	**Screening & Baseline**	**Enrolment**	**Randomizati on for**	**-**	**-**	**-**	**End of**
Informed Consent	X	-	-	-	-	-	-
Medical/Surgical History	X	X	-	-	-	-	X
Physical Examination, Vital Signs	X	X	X	X	X	X	X
Demographics including height, weight and BMI	X	X	X	X	X	X	X
Eligibility assessment	X	-	-	-	-	-	-
Randomization to treatment	-	-	X	-	-	-	-
Study drug dispensing	-		X	X	X	X	
Compliance to treatment	-			X	X	X	X
Subject diary dispensing	-	X					
Subject diary review	-	-	X	X	X	X	X
Subject diary retrieval	-	-	-	-	-	-	X
Laboratory Assessments* (Glycemic tests)	X	-	-	-	-	X	X
Laboratory Assessments$ (Renal tests)	X	-	-	-	-	-	X
Urine Pregnancy test#	X	-	-	-	-	-	
ECG	X	-	-	-	-	-	X
Prior and Concomitant Medications	X	X	X	X	X	X	X
CGM Implantation	-	X			X	-	-
CGM Removal	-	-	-	X	-	X	-
AE/SAE	X	X	X	X	X	X	X

* Glycemic parameters- HbA1c, FBS, PPBS
$ Renal parameters- RFT, UACR, e GFR, Serum Creatinine, BUNAE = adverse event; SAE = serious adverse event.# Female subjects only.

DocUUID : b897a93b-83ba-4de2-a309-31160c3ed1c4

9.5.2. Appropriateness of Measurements

All clinical measurements were performed using standard methods that were generally recognized as being reliable, accurate, and relevant. Most assessments and study procedures were as per standard protocols. The efficacy, safety endpoints used in this study were considered to be reliable, and relevant to the objectives set forth in the protocol.

9.5.3. Study Variables

Primary objective:

Primary objective was assessed to compare the effect of FDC of Teneligliptin 20 mg + Dapagliflozin 10 mg on 24-hour glucose levels with FDC of Sitagliptin 100 mg + Dapagliflozin 10 mg and FDC of Linagliptin 5 mg + Empagliflozin 25 mg assessed by parameters derived from continuous glucose monitoring in Indian patients of type 2 diabetes mellitus.

Secondary Objective:

Secondary objective was assessed

- To evaluate Glycaemic parameters of FDC of Teneligliptin 20 mg + Dapagliflozin 10 mg in comparison to FDC of Sitagliptin 100 mg + Dapagliflozin 10 mg and FDC of Linagliptin 5 mg + Empagliflozin 25 mg in Indian patients of type 2 diabetes mellitus
- To evaluate the safety and tolerability of FDC of Teneligliptin 20 mg + Dapagliflozin 10 mg in comparison to FDC of Sitagliptin 100 mg + Dapagliflozin 10 mg and FDC of Linagliptin 5 mg + Empagliflozin 25 mg in Indian patients of type 2 diabetes mellitus
- To assess effect on renal parameters of FDC of Teneligliptin 20 mg + Dapagliflozin 10 mg in comparison to FDC of Sitagliptin 100 mg + Dapagliflozin 10 mg and FDC of Empagliflozin 25 mg + Linagliptin 5 mg in Indian patients of type 2 diabetes mellitus.

9.5.4. Efficacy Assessments

Efficacy assessment parameters are as below

1. Assessment of glycemic variability at Immediate post randomization and at Visit 6 (Day 49) using a CGM device. Following CGM parameters will be evaluated-
    - Mean Blood Glucose
    - Standard deviation (SD) Blood glucose
    - Coefficient of variation of (CV) of 24-h blood glucose
    - Number of events & percentage time at glucose level <70, 70–140 (time-in-target), 70-180 (time-in-range), ≥140 mg/dL, ≥180 mg/dL
    - Mean amplitude of glycemic excursion (MAGE)
    - Largest amplitude of glycemic excursion (LAGE)
    - Mean of daily differences (MODD)
    - M value
    - Mean postprandial glucose excursion (MPPGE)
    - Maximum (Max) and Minimum blood glucose level (Min) before each meal
    - Blood glucose at 1 and 2 h after each meal

DocUUID : b897a93b-83ba-4de2-a309-31160c3ed1c4

2. HbA1c, FPG, PPG evaluation at baseline, Day 49 and Day 90
3. UACR, eGFR, Sr Creatinine and BUN assessment at baseline and Day 90

9.5.5. Safety Assessments

Safety evaluations involved tracking and documenting all adverse events (AEs) and serious adverse events (SAEs), consistent monitoring of hematology and blood chemistry, regular checks of vital signs and electrocardiograms (ECG), and conducting physical examinations in accordance with the Schedule of Assessments.

In case of premature discontinuation, the reason and their cause were documented. If the withdrawal is due to an AE, the AE was monitored until it is resolved or it has returned to a status that was prior to the AE.

The safety assessment included analyses of treatment emergent AEs, laboratory parameters.

Subject demography information was collected at the Screening visit. Demography information included date of birth (or age), sex and race/ethnicity. Other study-specific demography information included were Body weight (in kg), height (in cm), and body mass index (BMI) at all visits.

Medical and surgical history, current medical conditions were recorded at the Screening visit. All relevant medical and surgical history were noted in source documents and in the Medical & Surgical History form of the CRF.

Examination of Vital sign (ie, systolic and diastolic blood pressure [BP] [mmHg], heart rate [beats per minute], respiratory rate [per minute] and oral temperature [degrees in Farenheit]) by standard methods. Blood pressure and pulse were measured after the subject has been resting for 5 minutes. Blood pressure taken in the same manner (ie, seated or supine position) at every study visit. All BP measurements were performed on the same Arm, and preferably performed by the same person at the study site.

Documentation of the vital signs were included in the source documentation at the site. Significant findings at the Screening visit were recorded in the Medical & Surgical History form of the CRF. Changes from screening/baseline examination findings that meet the definition of an AE was recorded on the Adverse Events CRF.

Physical examinations (comprehensive or symptom directed/targeted examination) were performed as designated on the Schedule of Assessments. A comprehensive physical examination was included general appearance, skin/subcutaneous tissue, head and neck (including thyroid gland), eyes, ears, nose and throat, mouth, abdomen, lymph nodes, musculoskeletal, thorax, lungs/respiratory, heart/cardiovascular, urogenital, anal/rectal, extremities and a brief neurological/psychiatric examination. Urogenital and anal/rectal examinations are optional and sign/symptom-directed examination were performed as per the Investigator’s discretion.

Documentation of the physical examination was included in the physical examination section of the CRF and source documentation at the site. Significant findings at the Screening visit were recorded on the Medical & Surgical History CRF. Changes from screening/baseline examination findings that met the definition of an AE was recorded on the Adverse Events CRF.

An ECG abnormality meeting the criteria of an AE as described in the protocol and the CRF Completion Guidelines were recorded as an adverse event in the CRF. In these instances, the AE corresponding to the ECG abnormality was recorded on the Adverse Events CRF.

For ECG abnormalities meeting criteria of an SAE, the site was instructed to fax or email the SAE report including the ECG report (with Subject ID only, and subject’s name masked) to the Sponsor using the SAE form

The following laboratory assessments were conducted as per Schedule of assessments in local labs and whenever the Investigator feels investigations are necessary.

DocUUID : b897a93b-83ba-4de2-a309-31160c3ed1c4

Any abnormal clinical laboratory test results (hematology, blood chemistry or urinalysis) or other safety assessments (eg, ECGs, vital signs measurements) that worsen from baseline were considered to be clinically significant in the medical and scientific judgment of the Investigator are to be recorded as AEs or SAEs.

If a potential AE requires results of specific laboratory parameters faster than those analysed in the Local Laboratory, some parameters may have to be determined in a local laboratory in advance or in parallel. Those analyses may serve as guidance for the Investigator in determining particular requirements for care for a research participant. They do not constitute study data in the sense of this protocol. The only study data in the sense of this protocol are the centrally obtained laboratory results. However, should a given subject suffer from a serious adverse event (SAE), laboratory results from locally analyzed samples will become part of the relevant safety information during SAE reporting.

9.5.6. Drug Concentration Measurements

This section is not applicable to this study.

9.6. Data Quality Assurance

Quality control procedures were implemented and maintained to ensure the accuracy and reliability of the data. For any missing data, clarification was communicated to the sites for resolution. The study site agreed to provide direct access to all study-related source data/documents, and reports for the purposes of monitoring and auditing that might be conducted by the sponsor, and inspection by local and regulatory authorities. The documentation of the study was expected to be adequate for the reconstruction of the course of events. Following written SOPs, the Monitor verified that the clinical trial was conducted, and data were generated, documented (recorded), and reported in compliance with the study protocol, GCP, and the applicable regulatory requirements. On-site visits and/or remote monitoring visits were performed periodically at study sites. All records and documents pertaining to the study were maintained in the permanent files of the investigator and were available for inspection by the authorized party at any time.

9.6.1. Data and Safety Monitoring Board (DSMB)

Not applicable

9.6.2. Source Documents

Source data contains all information, records of clinical findings, observations, or other activities in a clinical study necessary for the reconstruction and evaluation of the study. Source data were contained in source documents. Examples of these original documents and data records included: hospital records, daily report, clinical source documents, investigator notes, laboratory reports, IP accountability logs or subject eligibility checklist, recorded data from automated instruments, copies or transcriptions certified after verification as being accurate and complete, subject files, and records kept at the pharmacy, at the laboratories, and at medico-technical departments involved in the clinical study.

DocUUID : b897a93b-83ba-4de2-a309-31160c3ed1c4

9.6.3. Monitoring

Study centres were monitored by the sponsor and the designated CRO. Centres were visited at regular intervals and a Visit Log was maintained for all on-site visits. Monitors were responsible for reviewing adherence to the protocol; compliance with GCP; and the completeness, accuracy, and consistency of the data. Direct access to subject medical and laboratory records was permitted to verify entries on the study-specific CRFs.

9.6.4. Investigator and Staff Training

Investigator staff training was provided by the sponsor and the designated CRO during the site initiation and routine monitoring visits. The sponsor organized investigator and clinical research associate meetings before the study start and during the study to provide information on the investigational product, the study rationale and design, responsibilities under ICH GCP and local regulatory requirements, and training on the detailed study requirements.

9.6.5. Laboratory Procedures

Local laboratories were used to measure laboratory parameters that are to be assessed as part of the safety analyses for the clinical study report (CSR). All the procedures regarding the collection, processing and shipping of samples were outlined in a separate lab manual.
The following laboratory assessments were conducted as per Schedule of assessments and whenever the Investigator feels investigations were necessary.

- Glycemic parameters- HbA1c, FBS, PPBS at screening/ baseline, Day 49 & at day 90.
- Renal parameters- RFT, UACR, e GFR, Serum Creatinine, BUN, at screening/ baseline & at Day 90.

Any abnormal clinical laboratory test results (hematology, blood chemistry or urinalysis) or other safety assessments (eg, ECGs, vital signs measurements) that worsen from baseline and considered to be clinically significant in the medical and scientific judgment of the Investigator were recorded as AEs or SAEs.
If a potential AE requires results of specific laboratory parameters faster than those analyzed in the Central Laboratory, some parameters may have to be determined in a local laboratory in advance or in parallel. Those analyses may serve as guidance for the Investigator in determining particular requirements for care for a research participant. They do not constitute study data in the sense of this protocol. The only study data in the sense of this protocol are the centrally obtained laboratory results. However, should a given subject suffer from a serious adverse event (SAE), laboratory results from locally analyzed samples were become part of the relevant safety information during SAE reporting.

9.6.6. Investigator Responsibilities

The investigators were responsible for all data entered in the CRFs and documentation of their review and approval of the data, verifying the validity and completeness of the data. The investigators continue to be responsible for the appropriate retention of essential study documents.

9.6.7. Clinical Data Management

Case report forms data was entered in database by a CRO team. Data is validated through edit checks; quality control of the data is performed and Queries were sent to the investigational site and the clarified correct data were entered into the database.

DocUUID : b897a93b-83ba-4de2-a309-31160c3ed1c4

9.6.8. Clinical Quality Assurance Audits

Not applicable.

9.7. Statistical Methods Planned in the Protocol and Determination of Sample Size

9.7.1. Statistical and Analytical Plans

The statistical analysis was coordinated by the responsible biostatistician. The Statistical Analysis Plan (SAP) was written to provide details of the analysis, along with specifications for tables, listings, and figures to be produced. The SAP was finalized before the database lock at the latest. If there are differences, the information in the SAP supersedes the information in the protocol. Any changes from the analyses planned in the SAP justified in the CSR. All analyses performed by responsible biostatistician, was documented. In general, all data will be summarized with descriptive statistics (number of subjects, mean, and standard deviation [SD], minimum, median, and maximum) for continuous variables and frequency and percentage for categorical variables.

9.7.2. Analysis Population

Safety Analysis set (SAS):

The Safety Set (SAF) includes all subjects who received at least one dose of the study drug treatment. The safety set was used for all safety analyses.

Full Analysis Set (FAS):

The Full Analysis Set (FAS) includes all enrolled subjects who have received at least one dose of study drug and have at least one post-baseline effectiveness assessment.

Per-Protocol set (PPS):

Per-Protocol set (PPS) includes all patients in the FAS who did not have any major protocol deviations.

9.7.3. Interim and Protocol-specified Analyses

Not applicable.

9.7.4. Primary Endpoint

The primary endpoint for the study was:

Mean change from baseline in each of parameters of glycemic variability at Immediate post-randomization and at visit 6 (day 49).

9.7.5. Secondary Endpoint

The Secondary endpoint for the study was to assess:

- Mean change from baseline in HbA1c, FPG and PPG, at visit 6 (day 49) and visit 7 (day 90)
- Mean change from baseline in UACR, eGFR, serum creatinine, BUN at visit 7 (day 90).
- Incidence of any treatment emergent adverse events in terms of abnormal signs, or laboratory reports.

9.7.6. Safety Analyses

Safety analysis was Safety data during the study period evaluated and summarized descriptively. Adverse events were summarized by system organ class and preferred term. Subjects were counted only once for each preferred term, system organ class, and by the highest severity of an event.

DocUUID : b897a93b-83ba-4de2-a309-31160c3ed1c4

Laboratory evaluations were summarized using descriptive statistics at each visit, and change from baseline summarized for each post-baseline visit. Vital signs (Pulse, RR, BP and Body temperature) summarized descriptively.

9.7.7. Determination of Sample Size

As this was proof of concept study, no formal sample size has been estimated. A sample size of 75 completed patients, ensuring 25 complete patients in each Arm was considered. Considering drop-out rate of 20%, total of 90 patients (30 in each group) were enrolled in the study.

9.8. Changes in the Conduct of the Study or Planned Analysis

9.8.1. Changes in the conduct of the study

No changes were made in conduct of the study and study was performed as per approved protocol.

9.8.2. Changes in the Planned Analyses

No changes were made in planned analysis and the analysis was performed as per approved protocol and SAP.

DocUUID : b897a93b-83ba-4de2-a309-31160c3ed1c4

10. STUDY SUBJECTS

90 adult Indians (Age ≥18 years) of either gender, diagnosed with Type 2 DM patients with HbA1c between ≥ 7.5 % to ≤ 10%

10.1. Subject Disposition

Among the 120 subjects screened, 30 subjects were screen failures and 90 subjects were enrolled into the study. 87 subjects completed the study as per the protocol.

Figure-10 1:Study Flow chart

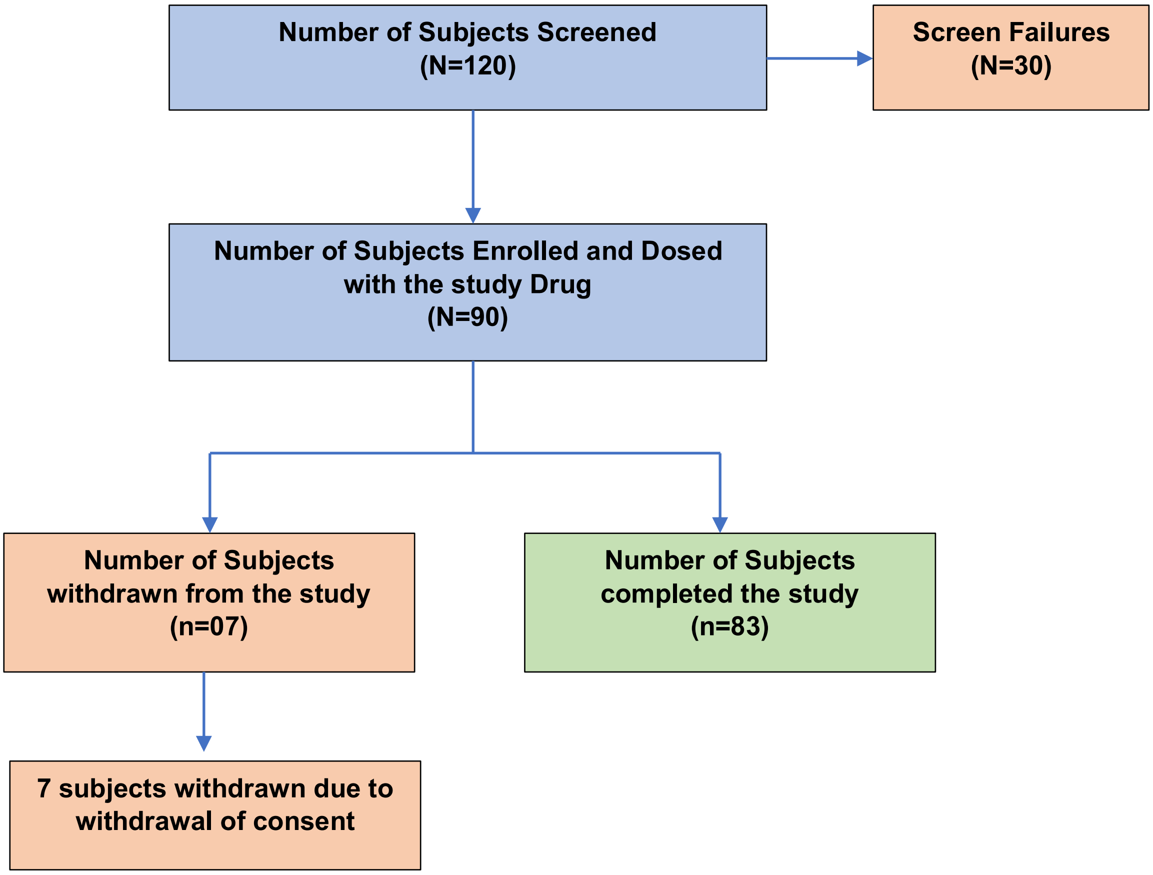

10.1.1. Handling of Dropouts or Missing Data

As part of the responsibilities assumed by participating in the study, the Investigators agreed to maintain adequate and accurate case histories for the subjects treated under this protocol. Case histories include CRFs and supporting data including, but not limited to, signed and dated informed consent forms, progress notes, hospital charts, nurse's notes, diary cards, laboratory reports, ECG strips, etc were maintained.

Subject demographics were collected, as available, for all subjects who provide written informed consent. For subjects who provided informed consent and were not assigned to treatment into the study, the reason the subject was not assigned to treatment, ie, did not meet one or more inclusion criteria, met one or more exclusion criteria, or other (eg, lost to follow-up, consent withdrawn) were also collected.

However, 90 subjects enrolled into the study and 7 subjects withdrawn from the study.

DocUUID : b897a93b-83ba-4de2-a309-31160c3ed1c4

10.2. Protocol Deviations

In 51 subjects, a total of 77 protocol deviations were reported during the study. Among the 77 protocol deviations,55 deviations were due to Visit conducted out of the window period, 21 deviations were due to subjects Missing blood sample for the Fasting Blood Sugar (FBS) Glucose Test at visit-6 and 1 deviation was due to Visit-7 conducted virtually.

Table-10 1: Summary of Protocol Deviations Details

Protocol Deviation	Arm-A (Teneligliptin 20 mg + Dapagliflozin 10 mg)	Arm-B (Sitagliptin 100 mg + Dapagliflozin 10 mg)	Arm-C (Empagliflozin 25 mg + Linagliptin 5 mg)
	n (%)	n (%)	n (%)
**Missing blood sample for the Fasting Blood Sugar (FBS) Glucose Test at visit-6**	8 (26.67%)	6 (20.00%)	7 (23.33%)
**Visit-7 conducted virtually**	-	-	1 (3.33%)
**Visit conducted out of the window period**	12 (40.00%)	8 (26.67%)	10 (33.33%)
**Grand Total**	**20 (66.67%)**	**14 (46.67%)**	**17 (56.67%)**

10.3. Populations Analysed

83 subjects completed the study as per the protocol. 7 subjects withdrawn from the study due to withdrawn of consent. 90 subjects comprised the safety Analysis set, full analysis set, PP Analysis set and the safety analysis set.

10.4. Demographic and Other Baseline Characteristics

A summary of key baseline demographic characteristics for the study subjects as presented in Table 10.2 and 10-3 below. Baseline characteristics summarize the age, gender, height, weight and BMI of the subjects.
A total of 90 subjects were enrolled in the study. Of the 90 subjects, 30 subjects were included in each group. In Arm-A (Teneligliptin 20 mg + Dapagliflozin 10 mg) 7 (23.33%) subjects were female, and 23 (76.67%) subjects were male and the mean age of the subjects was 54.07 years. In Arm-B (Sitagliptin 100 mg + Dapagliflozin 10 mg) 12 (40.00%) subjects were female, and 18 (60.00%) subjects were male and the mean age of the subjects was 54.07 years. Similarly, in Arm-C (Empagliflozin 25 mg + Linagliptin 5 mg) 11 (36.67%) subjects were female, and 19 (63.33%) subjects were male and the mean age of the subjects was 49.83 years.
The mean weight of the subjects in Arm A, Arm B and Arm C were 71.15 kgs, 66.25 kgs and 72.47 kgs respectively, Height of the subjects was 165.08 cms, 159.78 cms and 164.40 cms respectively and the mean BMI of the subjects was 25.98 kg/m^2, 25.15 kg/m^2 and 26.78 kg/m^2.

DocUUID : b897a93b-83ba-4de2-a309-31160c3ed1c4

Table-10 2: Key Baseline Demographic Characteristics

Parameters	Arm-A (Teneligliptin 20 mg + Dapagliflozin 10 mg)		Arm-B (Sitagliptin 100 mg + Dapagliflozin 10 mg)		Arm-C (Empagliflozin 25 mg + Linagliptin 5 mg)	
	n	Mean ± SD	n	Mean ± SD	n	Mean ± SD
**Age (years)**	30	54.07 ± 10.10	30	54.07 ± 10.60	30	49.83 ± 11.23
**Gender**						
Female	7	23.33%	12	40.00%	11	36.67%
Male	23	76.67%	18	60.00%	19	63.33%
**Weight (kg)**	30	71.15 ± 11.14	30	66.25 ± 10.64	30	72.47 ± 12.23
**Height (cm)**	30	165.08 ± 7.80	30	159.78 ± 13.53	30	164.40 ± 9.38
**BMI (kg/m^2)**	30	25.98 ± 2.70	30	25.15 ± 3.75	30	26.78 ± 4.19

Note: *n=Total number of subjects; SD=Standard Deviation.*

All baseline, there was no significance difference between the 3 groups in terms of demographic characters.

Table-10 3: Summary of baseline characteristics

Parameter	Visits	Arm-A (Teneligliptin 20 mg + Dapagliflozin 10 mg)		Arm-B (Sitagliptin 100 mg + Dapagliflozin 10 mg)		Arm-C (Empagliflozin 25 mg + Linagliptin 5 mg)	
		n	Mean ± SD	n	Mean ± SD	n	Mean ± SD
**Vitals**							
**Body Temperature (°F)**	Visit 1 (Day -3 to Day 0)	30	98.08 ± 0.51	30	98.13 ± 0.50	30	98.05 ± 0.62
**Systolic Blood Pressure (mmHg)**	Visit 1 (Day -3 to Day 0)	30	128.47 ± 6.35	30	127.10 ± 8.21	30	128.90 ± 12.27
**Diastolic Blood Pressure (mmHg)**	Visit 1 (Day -3 to Day 0)	30	83.10 ± 5.90	30	79.30 ± 8.24	30	82.70 ± 8.42
**Pulse Rate (beats/min)**	Visit 1 (Day -3 to Day 0)	30	80.53 ± 5.69	30	81.03 ± 8.32	30	80.43 ± 10.80
**Respiratory Rate (breaths/ min)**	Visit 1 (Day -3 to Day 0)	30	18.13 ± 2.85	30	18.40 ± 3.19	30	18.03 ± 2.95
**Glycemic Parameters**							
**HbA1c**	Visit 1 (Day -3 to Day 0)	30	8.47 ± 0.72	30	8.44 ± 0.79	30	8.81 ± 0.78
**FPG**	Visit 1 (Day -3 to Day 0)	30	144.00 ± 30.57	30	147.28 ± 45.68	30	147.29 ± 38.65
**PPG**	Visit 1 (Day -3 to Day 0)	30	194.02 ± 47.17	30	212.14 ± 77.62	30	195.80 ± 44.66
**Renal Parameters**							
**Urine Albumin Creatinine Ratio (UACR)**	Visit 1 (Day -3 to Day 0)	30	20.13 ± 37.46	30	23.18 ± 23.60	30	18.12 ± 13.30
**Estimated Glomerular Filtration Rate (eGFR)**	Visit 1 (Day -3 to Day 0)	30	94.22 ± 16.54	30	98.55 ± 28.18	30	101.05 ± 23.28
**Serum Creatinine**	Visit 1 (Day -3 to Day 0)	30	0.90 ± 0.19	30	0.86 ± 0.22	30	0.84 ± 0.25
**Blood urea nitrogen (BUN)**	Visit 1 (Day -3 to Day 0)	30	13.68 ± 7.06	30	11.45 ± 2.77	30	14.69 ± 9.26

DocUUID : b897a93b-83ba-4de2-a309-31160c3ed1c4

Table-10 4: Summary of Vital signs in individual groups visit wise

Parameters	Visits	Arm-A (Teneligliptin 20 mg + Dapagliflozin 10 mg)		Arm-B (Sitagliptin 100 mg + Dapagliflozin 10 mg)		Arm-C (Empagliflozin 25 mg + Linagliptin 5 mg)	
		n	Mean ± SD	n	Mean ± SD	n	Mean ± SD
Body Temperature (°F)	Visit 1 (Day -3 to Day 0)	30	98.08 ± 0.51	30	98.13 ± 0.50	30	98.05 ± 0.62
	Visit 2 (Day 1)	30	97.95 ± 0.51	28	98.09 ± 0.55	29	98.14 ± 0.62
	Visit 3 (Day 5 ± 1 Day)	30	97.36 ± 3.87	28	98.12 ± 0.48	29	98.00 ± 0.63
	Visit 4: (Day 14)	30	98.00 ± 0.51	28	98.03 ± 0.53	29	98.03 ± 0.55
	Visit 5: (Day 35)	29	97.95 ± 0.50	28	97.94 ± 0.41	26	97.87 ± 0.52
	Visit 6: (Day 49)	29	97.89 ± 0.61	28	98.05 ± 0.47	26	97.99 ± 0.52
	Visit 7: (Day 90)	29	97.92 ± 0.59	28	97.89 ± 0.55	25	98.02 ± 0.51
Systolic Blood Pressure (mmHg)	Visit 1 (Day -3 to Day 0)	30	128.47 ± 6.35	30	127.10 ± 8.21	30	128.90 ± 12.27
	Visit 2 (Day 1)	30	127.60 ± 9.84	28	126.43 ± 6.89	29	128.48 ± 10.57
	Visit 3 (Day 5 ± 1 Day)	30	128.93 ± 7.30	28	128.21 ± 8.99	29	127.52 ± 8.99
	Visit 4: (Day 14)	30	125.53 ± 5.88	28	127.96 ± 7.64	29	125.45 ± 9.13
	Visit 5: (Day 35)	29	125.41 ± 8.86	28	127.18 ± 6.35	26	123.81 ± 8.94
	Visit 6: (Day 49)	29	129.24 ± 11.25	28	124.21 ± 9.06	26	124.73 ± 7.11
	Visit 7: (Day 90)	29	129.28 ± 9.31	28	125.89 ± 9.76	25	123.84 ± 7.37
Diastolic Blood Pressure (mmHg)	Visit 1 (Day -3 to Day 0)	30	83.10 ± 5.90	30	79.30 ± 8.24	30	82.70 ± 8.42
	Visit 2 (Day 1)	30	81.67 ± 5.57	28	79.00 ± 6.85	29	81.83 ± 6.21
	Visit 3 (Day 5 ± 1D)	30	81.27 ± 5.63	28	78.96 ± 5.89	29	81.59 ± 5.78
	Visit 4: (Day 14)	30	79.50 ± 5.39	28	78.86 ± 6.26	29	80.07 ± 6.89
	Visit 5: (Day 35)	29	80.38 ± 5.94	28	79.21 ± 3.94	26	79.00 ± 6.84
	Visit 6: (Day 49)	29	82.03 ± 10.10	28	78.64 ± 7.98	26	79.81 ± 6.67
	Visit 7: (Day 90)	29	80.55 ± 6.14	28	77.18 ± 6.85	25	80.12 ± 6.09
Pulse rate (beats/min)	Visit 1 (Day -3 to Day 0)	30	80.53 ± 5.69	30	81.03 ± 8.32	30	80.43 ± 10.80
	Visit 2 (Day 1)	30	80.13 ± 6.24	28	79.18 ± 6.76	29	83.10 ± 13.35
	Visit 3 (Day 5 ± 1 Day)	30	80.00 ± 7.63	28	80.61 ± 10.07	29	81.86 ± 9.82
	Visit 4: (Day 14)	30	78.47 ± 4.45	28	79.64 ± 9.57	29	82.55 ± 10.39
	Visit 5: (Day 35)	29	80.45 ± 7.64	28	80.25 ± 9.58	26	80.69 ± 10.71
	Visit 6: (Day 49)	29	80.79 ± 7.69	28	78.07 ± 6.86	26	81.65 ± 11.07
	Visit 7: (Day 90)	29	81.07 ± 8.69	28	77.89 ± 8.87	25	81.52 ± 10.84
Respiratory rate (breaths/ min)	Visit 1 (Day -3 to Day 0)	30	18.13 ± 2.85	30	18.40 ± 3.19	30	18.03 ± 2.95
	Visit 2 (Day 1)	30	17.90 ± 2.83	28	17.64 ± 3.09	29	18.24 ± 3.03
	Visit 3 (Day 5 ± 1 Day)	30	17.77 ± 2.28	28	18.68 ± 2.98	29	17.93 ± 3.05
	Visit 4: (Day 14)	30	17.90 ± 2.94	28	18.50 ± 3.04	29	18.24 ± 2.60
	Visit 5: (Day 35)	29	17.93 ± 2.52	28	18.18 ± 3.20	26	18.54 ± 3.50
	Visit 6: (Day 49)	29	18.83 ± 3.90	28	18.43 ± 3.17	26	17.85 ± 2.38
	Visit 7: (Day 90)	29	18.00 ± 2.49	28	17.89 ± 2.51	25	18.12 ± 3.00

All baseline, there was no significance difference between the 3 groups in terms of Vital signs.

DocUUID : b897a93b-83ba-4de2-a309-31160c3ed1c4

10.5. Prior and Concomitant Medications

A total of 90 subjects (100.00%) have taken concomitant medications during the study period. Among the 90 subjects, 2 subjects (6.67%) in Arm-B, 3 subjects (10.00%) in Arm-C have taken Analgesics and Anti Pyretic, 1 subject (3.33%) in Arm-B have taken Angiotensin Receptor Blockers, 1 subject (3.33%) in Arm-B have taken Antacids, 1 subject (3.33%) in Arm-B have taken Antibiotics, 1 subject (3.33%) in Arm-C have taken Anticonvulsants + Tricyclic Antidepressants + Multivitamin, 1 subject (3.33%) in Arm-B, 1 subject (3.33%) in Arm-C has taken Antihistamines, 1 subject (3.33%) in Arm-C have taken BCS Class II, 2 subjects (6.67%) in Arm-A, 3 subjects (10.00%) in Arm-B, 5 subjects (16.67%) in Arm-C has taken Benzimidazoles, 1 subject (3.33%) in Arm-B, 1 subject (3.33%) in Arm-C has taken Benzimidazoles + Calcium Channel Blockers, 2 subjects (6.67%) in Arm-A, 3 subjects (10.00%) in Arm-B has taken Beta Blockers, 27 subjects (97.00%) in Arm-A, 26 subjects (86.67%) in Arm-B, 26 subjects (86.67%) in Arm-C has taken Biguanides, 1 subject (3.33%) in Arm-C have taken Biguanides + DPP-4 Inhibitors, 3 subjects (10.00%) in Arm-A, 6 subjects (20.00%) in Arm-B, 4 subjects (13.33%) in Arm-C has taken Biguanides + Sulfonylurea, 1 subject (3.33%) in Arm-A have taken Biguanides + Thiazolidinedione, 1 subject (3.33%) in Arm-A have taken Bronchodilators + Leukotriene Receptor Antagonists (LTRAS), 1 subject (3.33%) in Arm-A have taken Calcium Carbonates, 3 subjects (10.00%) in Arm-A, 3 subjects (10.00%) in Arm-B, 5 subjects (16.67%) in Arm-C has taken Calcium Channel Blockers, 1 subject (3.33%) in Arm-A have taken Calcium Channel Blockers + Angiotensin Receptor Blocking (ARB), 1 subject (3.33%) in Arm-B have taken Corticosteroids, 1 subject (3.33%) in Arm-C have taken COX-2 Inhibitors + Other Centrally-Acting Muscle Relaxants, 1 subject (3.33%) in Arm-A have taken D-Alanine Aminotransferase, 1 subject (3.33%) in Arm-C have taken Dihydropyridine Calcium Channel Blocker + Angiotensin II Receptor Antagonists, 1 subject (3.33%) in Arm-A have taken Dipeptidyl Peptidase-4 (DPP-4) Inhibitors, 1 subject (3.33%) in Arm-A have taken Diuretics, 2 subjects (6.67%) in Arm-C have taken Dopamine Antagonists, 1 subject (3.33%) in Arm-A have taken Expectorant, 1 subject (3.33%) in Arm-B have taken Gastrointestinal Agents, 1 subject (3.33%) in Arm-A have taken Calcium Carbonates, 6 subjects (20.00%) in Arm-A, 3 subjects (10.00%) in Arm-B, 9 subjects (30.00%) in Arm-C has taken HMG-CoA Reductase Inhibitors (Statins), 1 subject (3.33%) in Arm-A, 1 subject (3.33%) in Arm-B has taken Imidazole, 1 subject (3.33%) in Arm-B have taken Nasal Decongestants, 1 subject (3.33%) in Arm-C have taken NSAIDS + Analgesic and Antipyretic, 1 subject (3.33%) in Arm-B have taken Opiate Analgesics, 1 subject (3.33%) in Arm-B have taken Probiotics, 1 subject (3.33%) in Arm-B, 1 subject (3.33%) in Arm-C has taken Proton-Pump Inhibitors, 1 subject (3.33%) in Arm-B have taken Proton-Pump Inhibitors + Dopamine Antagonists, 1 subject (3.33%) in Arm-C have taken Selective Serotonin Reuptake Inhibitors + Benzodiazepines, 1 subject (3.33%) in Arm-A have taken Substituted Benzimidazole Proton-Pump Inhibitors, 1 subject (3.33%) in Arm-B have taken Substituted Benzimidazole Proton-Pump Inhibitors + Dopamine Antagonists, 6 subjects (20.00%) in Arm-A, 3 subjects (10.00%) in Arm-B, 7 subjects (23.33%) in Arm-C has taken Sulfonylurea, 1 subject (3.33%) in Arm-B have taken Thiazide Diuretics, 1 subject (3.33%) in Arm-B have taken Thiazolidinedione 1 subject (3.33%) in Arm-B have taken Thyroid Agents, 1 subject (3.33%) in Arm-B have taken Triazoles, 1 subject (3.33%) in Arm-C have taken Tricyclic Antidepressant (TCA) + Cyanocobalamin and Analogues, 1 subject (3.33%) in Arm-B have taken Urinary Alkalizer, 1 subject (3.33%) in Arm-B have taken Xanthine Oxidase Inhibitors and 7 subjects (23.33%) in Arm-A, 8 subjects (26.67%) in Arm-B, 6 subjects (20.00%) in Arm-C has taken Others.

DocUUID : b897a93b-83ba-4de2-a309-31160c3ed1c4

Table-10 5: Summary of Concomitant medications

Drug Classification	Arm-A (Teneligliptin 20 mg + Dapagliflozin 10 mg)	Arm-B (Sitagliptin 100 mg + Dapagliflozin 10 mg)	Arm-C (Empagliflozin 25 mg + Linagliptin 5 mg)
	n (%)	n (%)	n (%)
Analgesics And Anti Pyrectics	-	2 (6.67%)	3 (10.00%)
Angiotensin Receptor Blockers	-	1 (3.33%)	-
Antacids	-	1 (3.33%)	-
Antibiotic	-	1 (3.33%)	-
Anticonvulsants + Tricyclic Antidepressants + Multivitamin	-	-	1 (3.33%)
Antihistamines	-	1 (3.33%)	1 (3.33%)
BCS Class II	-	-	1 (3.33%)
Benzimidazoles	2 (6.67%)	3 (10.00%)	5 (16.67%)
Benzimidazoles + Calcium Channel Blockers	-	1 (3.33%)	1 (3.33%)
Beta Blockers	2 (6.67%)	3 (10.00%)	-
Biguanides	27 (90.00%)	26 (86.67%)	26 (86.67%)
Biguanides + DPP-4 Inhibitors	-	-	1 (3.33%)
Biguanides + Sulfonylurea	3 (10.00%)	6 (20.00%)	4 (13.33%)
Biguanides + Thiazolidinedione	1 (3.33%)	-	-
Bronchodilators + Leukotriene Receptor Antagonists (LTRAS)	1 (3.33%)	-	-
Calcium Carbonates	1 (3.33%)	-	-
Calcium Channel Blockers	3 (10.00%)	3 (10.00%)	5 (16.67%)
Calcium Channel Blockers + Angiotensin Receptor Blocking (ARB)	1 (3.33%)	-	-
Corticosteroids	-	1 (3.33%)	-
COX-2 Inhibitors + Other Centrally-Acting Muscle Relaxants	-	-	1 (3.33%)
D-Alanine Aminotransferase	1 (3.33%)	-	-
Dihydropyridine Calcium Channel Blocker + Angiotensin II Receptor Antagonists	-	-	1 (3.33%)
Dipeptidyl Peptidase-4 (DPP-4) Inhibitors	1 (3.33%)	-	-
Diuretic	1 (3.33%)	-	-
Dopamine Antagonists	-	-	2 (6.67%)
Expectorant	1 (3.33%)	-	-
Gastrointestinal Agents	-	1 (3.33%)	-
HMG-CoA Reductase Inhibitors (Statins)	6 (20.00%)	3 (10.00%)	9 (30.00%)
Imidazole	1 (3.33%)	1 (3.33%)	-
Nasal Decongestants	-	1 (3.33%)	-
NSAIDS + Analgesic and Antipyretic	-	-	1 (3.33%)
Opiate Analgesics	-	1 (3.33%)	-
Probiotic	-	1 (3.33%)	-
Proton-Pump Inhibitors	-	1 (3.33%)	1 (3.33%)
Proton-Pump Inhibitors + Dopamine Antagonists	-	1 (3.33%)	-
Selective Serotonin Reuptake Inhibitors + Benzodiazepines	-	-	1 (3.33%)

DocUUID : b897a93b-83ba-4de2-a309-31160c3ed1c4

Drug Classification	Arm-A (Teneligliptin 20 mg + Dapagliflozin 10 mg)	Arm-B (Sitagliptin 100 mg + Dapagliflozin 10 mg)	Arm-C (Empagliflozin 25 mg + Linagliptin 5 mg)
	n (%)	n (%)	n (%)
Substituted Benzimidazole Proton-Pump Inhibitors	1 (3.33%)	-	-
Substituted Benzimidazole Proton-Pump Inhibitors + Dopamine Antagonists	-	1 (3.33%)	-
Sulfonylurea	6 (20.00%)	3 (10.00%)	7 (23.33%)
Thiazide Diuretics	-	1 (3.33%)	2 (6.67%)
Thiazolidinedione	-	1 (3.33%)	-
Thyroid Agents	-	1 (3.33%)	-
Triazoles	-	1 (3.33%)	-
Tricyclic Antidepressant (TCA) + Cyanocobalamin and Analogues	-	-	1 (3.33%)
Urinary Alkalizer	-	1 (3.33%)	-
Xanthine Oxidase Inhibitors	-	1 (3.33%)	-
Others	7 (23.33%)	8 (26.67%)	6 (20.00%)

Note: *n-Number of Subjects, percentage is calculated over total study population of the group.*

10.6. Study Intervention Exposure and Compliance

A total of 90 adult Indian (Age ≥18 years) of either gender, diagnosed with Type 2 DM patients with HbA1c between ≥ 7.5 % to ≤ 10%, were enrolled into the study and 87 subjects completed the study.

11. EFFICACY ASSESSMENT

Table-11 1: Summary of Glycemic parameters (Average Glucose, TIR, TBR, TAR) during different phases of the study with baseline comparison

Parameters	Visits	Arm-A (Teneligliptin 20 mg + Dapagliflozin 10 mg)			Arm-B (Sitagliptin 100 mg + Dapagliflozin 10 mg)			Arm-C (Empagliflozin 25 mg + Linagliptin 5 mg)		
		n	Mean ± SD	p-value	N	Mean ± SD	p-value	n	Mean ± SD	p-value
**Average Glucose (mg/dL)**	Pre-treatment	30	161.4 ± 57.8	-	28	176.4 ± 53.0	-	29	181.6 ± 53.5	-
	End of Phase I	29	122.4 ± 45.4	0.001	28	152.4 ± 50.4	0.029	27	140.0 ± 31.7	<0.001
	End of Phase II	26	126.2 ± 33.3	<0.001	26	142.2 ± 41.8	0.002	25	127.4 ± 27.3	<0.001
**TIR (%)**	Pre-treatment	30	54.5 ± 24.9	-	28	42.7 ± 26.8	-	29	42.3 ± 28.2	-
	End of Phase I	29	68.8 ± 25.6	0.023	28	53.6 ± 34.1	0.063	27	68.2 ± 28.1	<0.001
	End of Phase II	26	78.1 ± 21.5	<0.001	26	65.9 ± 28.7	0.001	25	72.1 ± 24.1	<0.001
**TBR (%)**	Pre-treatment	30	5.5 ± 8.1	-	28	2.7 ± 4.0	-	29	2.6 ± 5.3	-
	End of Phase I	29	14.6 ± 23.7	0.061	28	5.1 ± 12.5	0.256	27	5.7 ± 15.8	0.244

DocUUID : b897a93b-83ba-4de2-a309-31160c3ed1c4

Parameters	Visits	Arm-A (Teneligliptin 20 mg + Dapagliflozin 10 mg)			Arm-B (Sitagliptin 100 mg + Dapagliflozin 10 mg)			Arm-C (Empagliflozin 25 mg + Linagliptin 5 mg)		
		n	Mean ± SD	p-value	N	Mean ± SD	p-value	n	Mean ± SD	p-value
	End of Phase II	26	6.3 ± 9.4	0.427	26	2.1 ± 5.1	0.645	25	6.6 ± 17.6	0.292
TAR (%)	Pre-treatment	30	40.1 ± 28.1	-	28	54.6 ± 28.9	-	29	55.0 ± 29.7	-
	End of Phase I	29	16.6 ± 22.9	<0.001	28	41.3 ± 36.8	0.029	27	25.6 ± 27.6	<0.001
	End of Phase II	26	15.6 ± 21.7	<0.001	26	32.0 ± 30.0	0.001	25	21.3 ± 22.1	<0.001

Note: p-value is calculated using paired t-test.

Summary:

Glycaemic parameters baseline comparison, Mean average glucose levels of subjects at pre-treatment was 161.4 ± 57.8, 176.4 ± 53.0 and 181.6 ± 53.5 respectively in Arm-A, Arm-B and Arm-C. At End of Phase I Average Glucose levels were 122.4 ± 45.4, 152.4 ± 50.4 and 140.0 ± 31.7 in Arm-A, Arm-B and Arm-C. At End of Phase II Average Glucose levels were 126.2 ± 33.3, 142.2 ± 41.8 and 127.4 ± 27.3 in Arm-A, Arm-B and Arm-C. Mean TIR (%) during Pre-treatment 54.5 ± 24.9, 42.7 ± 26.8 and 42.3 ± 28.2 in Arm-A, Arm-B and Arm-C. At End phase I Mean TIR (%) 68.8 ± 25.6, 53.6 ± 34.1 and 68.2 ± 28.1 in Arm-A, Arm-B and Arm-C. At End phase II Mean TIR (%) 78.1 ± 21.5, 65.9 ± 28.7 and 72.1 ± 24.1 in Arm-A, Arm-B and Arm-C. Mean TBR (%) during Pre-treatment 5.5 ± 8.1, 2.7 ± 4.0 and 2.6 ± 5.3 in Arm-A, Arm-B and Arm-C. At End phase I Mean TBR (%) 14.6 ± 23.7, 5.1 ± 12.5 and 5.7 ± 15.8in Arm-A, Arm-B and Arm-C. At End phase II Mean TBR (%) 6.3 ± 9.4, 2.1 ± 5.1 and 6.6 ± 17.6 in Arm-A, Arm-B and Arm-C. Mean TAR (%) during Pre-treatment 40.1 ± 28.1, 54.6 ± 28.9 and 55.0 ± 29.7 in Arm-A, Arm-B and Arm-C. At End phase I Mean TAR (%) 16.6 ± 22.9, 41.3 ± 36.8 and 25.6 ± 27.6 in Arm-A, Arm-B and Arm-C. At End phase II Mean TAR (%) 15.6 ± 21.7, 32.0 ± 30.0 and 21.3 ± 22.1 in Arm-A, Arm-B and Arm-C.

Table-11 2: Summary of Glycemic parameters (Average Glucose, TIR, TBR, TAR) during different phases of the study comparing between the group

Parameters	Visits	Arm-A (Teneligliptin 20 mg + Dapagliflozin 10 mg)		Arm-B (Sitagliptin 100 mg + Dapagliflozin 10 mg)		Arm-C (Empagliflozin 25 mg + Linagliptin 5 mg)		p-value
		n	Mean ± SD	n	Mean ± SD	n	Mean ± SD	
Average Glucose (mg/dL)	Pre-treatment	30	161.4 ± 57.8	28	176.4 ± 53.0	29	181.6 ± 53.5	[a] 0.305 [b] 0.169 [c] 0.718
	End of Phase I	29	122.4 ± 45.4	28	152.4 ± 50.4	27	140.0 ± 31.7	[a] 0.022 [b] 0.098 [c] 0.276
	End of Phase II	26	126.2 ± 33.3	26	142.2 ± 41.8	25	127.4 ± 27.3	[a] 0.134 [b] 0.888 [c] 0.141
TIR (%)	Pre-treatment	30	54.5 ± 24.9	28	42.7 ± 26.8	29	42.3 ± 28.2	[a] 0.090 [b] 0.086 [c] 0.959

DocUUID : b897a93b-83ba-4de2-a309-31160c3ed1c4

Parameters	Visits	Arm-A (Teneligliptin 20 mg + Dapagliflozin 10 mg)		Arm-B (Sitagliptin 100 mg + Dapagliflozin 10 mg)		Arm-C (Empagliflozin 25 mg + Linagliptin 5 mg)		p-value
		n	Mean ± SD	n	Mean ± SD	n	Mean ± SD	
	End of Phase I	29	68.8 ± 25.6	28	53.6 ± 34.1	27	68.2 ± 28.1	[a] 0.063 [b] 0.933 [c] 0.089
	End of Phase II	26	78.1 ± 21.5	26	65.9 ± 28.7	25	72.1 ± 24.1	[a] 0.091 [b] 0.354 [c] 0.410
**TBR (%)**	Pre-treatment	30	5.5 ± 8.1	28	2.7 ± 4.0	29	2.6 ± 5.3	[a] 0.103 [b] 0.114 [c] 0.939
	End of Phase I	29	14.6 ± 23.7	28	5.1 ± 12.5	27	5.7 ± 15.8	[a] 0.063 [b] 0.103 [c] 0.870
	End of Phase II	26	6.3 ± 9.4	26	2.1 ± 5.1	25	6.6 ± 17.6	[a] 0.053 [b] 0.942 [c] 0.229
**TAR (%)**	Pre-treatment	30	40.1 ± 28.1	28	54.6 ± 28.9	29	55.0 ± 29.7	[a] 0.058 [b] 0.052 [c] 0.958
	End of Phase I	29	16.6 ± 22.9	28	41.3 ± 36.8	27	25.6 ± 27.6	[a] 0.004 [b] 0.191 [c] 0.078
	End of Phase II	26	15.6 ± 21.7	26	32.0 ± 30.0	25	21.3 ± 22.1	[a] 0.029 [b] 0.357 [c] 0.155

Note: *p-value is calculated using unpaired t-test. ([a]- Arm-A vs Arm-B, [b]- Arm-A vs Arm-C, [c]- Arm-B vs Arm-C).*

Summary:

Glycaemic parameters comparing between the group, Mean average glucose levels of subjects at pre-treatment was 161.4 ± 57.8, 176.4 ± 53.0 and 181.6 ± 53.5 respectively in Arm-A, Arm-B and Arm-C. At End of Phase I Average Glucose levels were 122.4 ± 45.4, 152.4 ± 50.4 and 140.0 ± 31.7 in Arm-A, Arm-B and Arm-C. At End of Phase II Average Glucose levels were 126.2 ± 33.3, 142.2 ± 41.8 and 127.4 ± 27.3 in Arm-A, Arm-B and Arm-C. Mean TIR (%) during Pre-treatment 54.5 ± 24.9, 42.7 ± 26.8 and 42.3 ± 28.2 in Arm-A, Arm-B and Arm-C. At End phase I Mean TIR (%) 68.8 ± 25.6, 53.6 ± 34.1 and 68.2 ± 28.1 in Arm-A, Arm-B and Arm-C. At End phase II Mean TIR (%) 78.1 ± 21.5, 65.9 ± 28.7 and 72.1 ± 24.1 in Arm-A, Arm-B and Arm-C. Mean TBR (%) during Pre-treatment 5.5 ± 8.1, 2.7 ± 4.0 and 2.6 ± 5.3 in Arm-A, Arm-B and Arm-C. At End phase I Mean TBR (%) 14.6 ± 23.7, 5.1 ± 12.5 and 5.7 ± 15.8in Arm-A, Arm-B and Arm-C. At End phase II Mean TBR (%) 6.3 ± 9.4, 2.1 ± 5.1 and 6.6 ± 17.6 in Arm-A, Arm-B and Arm-C. Mean TAR (%) during Pre-treatment 40.1 ± 28.1, 54.6 ± 28.9 and 55.0 ± 29.7 in Arm-A, Arm-B and Arm-C. At End phase I Mean TAR (%) 16.6 ± 22.9, 41.3 ± 36.8 and 25.6 ± 27.6 in Arm-A, Arm-B and Arm-C. At End phase II Mean TAR (%) 15.6 ± 21.7, 32.0 ± 30.0 and 21.3 ± 22.1 in Arm-A, Arm-B and Arm-C.

DocUUID : b897a93b-83ba-4de2-a309-31160c3ed1c4

Table-11 3: Summary of Glycemic parameters (MAGE, LAGE, SD, CV) during different phases of the study with baseline comparison

Parameters	Visits	Arm-A (Teneligliptin 20 mg + Dapagliflozin 10 mg)			Arm-B (Sitagliptin 100 mg + Dapagliflozin 10 mg)			Arm-C (Empagliflozin 25 mg + Linagliptin 5 mg)		
		n	Mean ± SD	p-value	N	Mean ± SD	p-value	n	Mean ± SD	p-value
MAGE (mg/dL)	Pre-treatment	30	80.6 ± 19.2	-	28	86.1 ± 21.3	-	29	87.4 ± 19.0	-
	End of Phase I	29	61.2 ± 21.3	<0.001	28	66.7 ± 20.9	0.001	27	57.4 ± 21.0	<0.001
	End of Phase II	26	58.0 ± 13.8	<0.001	26	65.5 ± 26.2	<0.001	25	59.9 ± 17.3	<0.001
LAGE (mg/dL)	Pre-treatment	30	122.8 ± 31.9	-	28	132.4 ± 31.7	-	29	136.3 ± 28.3	-
	End of Phase I	29	96.8 ± 30.7	0.003	28	101.2 ± 36.7	<0.001	27	84.9 ± 28.1	<0.001
	End of Phase II	26	89.5 ± 21.5	<0.001	26	103.3 ± 46.6	<0.001	25	89.6 ± 33.4	<0.001
SD	Pre-treatment	30	54.0 ± 12.8	-	28	57.3 ± 14.2	-	29	57.7 ± 11.0	-
	End of Phase I	29	44.5 ± 14.8	0.018	28	44.5 ± 14.9	<0.001	27	40.2 ± 13.2	<0.001
	End of Phase II	26	40.8 ± 11.2	<0.001	26	46.1 ± 18.9	<0.001	25	41.1 ± 12.9	<0.001
CV (%)	Pre-treatment	30	35.7 ± 10.4	-	28	33.6 ± 8.4	-	29	33.1 ± 6.4	-
	End of Phase I	29	36.0 ± 10.6	0.989	28	30.0 ± 11.1	0.095	27	28.6 ± 9.3	0.028
	End of Phase II	26	33.6 ± 8.0	0.44	26	32.1 ± 9.6	0.375	25	31.5 ± 7.0	0.219

Note: p-value is calculated using paired t-test.

Summary:

Glycaemic parameters baseline comparison, Mean MAGE (mg/dL) of subjects at pre-treatment was 80.6 ± 19.2, 86.1 ± 21.3 and 86.1 ± 21.3 respectively in Arm-A, Arm-B and Arm-C. At End of Phase I MAGE (mg/dl) were 61.2 ± 21.3, 66.7 ± 20.9 and 57.4 ± 21.0in Arm-A, Arm-B and Arm-C. At End of Phase II MAGE (mg/dL) were 58.0 ± 13.8, 65.5 ± 26.2 and 59.9 ± 17.3 in Arm-A, Arm-B and Arm-C. Mean LAGE (mg/dL) during Pre-treatment 122.8 ± 31.9, 132.4 ± 31.7 and 136.3 ± 28.3 in Arm-A, Arm-B and Arm-C. At End phase I Mean LAGE (mg/dL) 96.8 ± 30.7, 101.2 ± 36.7 and 84.9 ± 28.1 in Arm-A, Arm-B and Arm-C. At End phase II Mean LAGE (mg/dL) 89.5 ± 21.5, 103.3 ± 46.6and 89.6 ± 33.4 in Arm-A, Arm-B and Arm-C. Mean SD during Pre-treatment 54.0 ± 12.8, 57.3 ± 14.2 and 57.7 ± 11.0 in Arm-A, Arm-B and Arm-C. At End phase I Mean SD 44.5 ± 14.8, 44.5 ± 14.9 and 40.2 ± 13.2 in Arm-A, Arm-B and Arm-C. At End phase II Mean SD 40.8 ± 11.2, 46.1 ± 18.9 and 41.1 ± 12.9 in Arm-A, Arm-B and Arm-C. Mean CV (%) during Pre-treatment 35.7 ± 10.4, 33.6 ± 8.4 and 33.1 ± 6.4 in Arm-A, Arm-B and Arm-C. At End phase I Mean CV (%) 36.0 ± 10.6, 30.0 ± 11.1 and 28.6 ± 9.3 in Arm-A, Arm-B and Arm-C. At End phase II Mean CV (%) 33.6 ± 8.0, 32.1 ± 9.6 and 31.5 ± 7.0 in Arm-A, Arm-B and Arm-C.

DocUUID : b897a93b-83ba-4de2-a309-31160c3ed1c4

Table-11 4: Summary of Glycemic parameters (MAGE, LAGE, SD, CV) during different phases of the study comparing between the group

Parameters	Visits	Arm-A (Teneligliptin 20 mg + Dapagliflozin 10 mg)		Arm-B (Sitagliptin 100 mg + Dapagliflozin 10 mg)		Arm-C (Empagliflozin 25 mg + Linagliptin 5 mg)		p-value
		n	Mean ± SD	n	Mean ± SD	n	Mean ± SD	
MAGE (mg/dL)	Pre-treatment	30	80.6 ± 19.2	28	86.1 ± 21.3	29	87.4 ± 19.0	[a] 0.386 [b] 0.179 [c] 0.672
	End of Phase I	29	61.2 ± 21.3	28	66.7 ± 20.9	27	57.4 ± 21.0	[a] 0.329 [b] 0.497 [c] 0.103
	End of Phase II	26	58.0 ± 13.8	26	65.5 ± 26.2	25	59.9 ± 17.3	[a] 0.207 [b] 0.674 [c] 0.372
LAGE (mg/dL)	Pre-treatment	30	122.8 ± 31.9	28	132.4 ± 31.7	29	136.3 ± 28.3	[a] 0.313 [b] 0.091 [c] 0.510
	End of Phase I	29	96.8 ± 30.7	28	101.2 ± 36.7	27	84.9 ± 28.1	[a] 0.625 [b] 0.137 [c] 0.070
	End of Phase II	26	89.5 ± 21.5	26	103.3 ± 46.6	25	89.6 ± 33.4	[a] 0.179 [b] 0.990 [c] 0.232
SD	Pre-treatment	30	54.0 ± 12.8	28	57.3 ± 14.2	29	57.7 ± 11.0	[a] 0.436 [b] 0.232 [c] 0.763
	End of Phase I	29	44.5 ± 14.8	28	44.5 ± 14.9	27	40.2 ± 13.2	[a] 0.997 [b] 0.251 [c] 0.256
	End of Phase II	26	40.8 ± 11.2	26	46.1 ± 18.9	25	41.1 ± 12.9	[a] 0.231 [b] 0.932 [c] 0.279
CV (%)	Pre-treatment	30	35.7 ± 10.4	28	33.6 ± 8.4	29	33.1 ± 6.4	[a] 0.336 [b] 0.242 [c] 0.905
	End of Phase I	29	36.0 ± 10.6	28	30.0 ± 11.1	27	28.6 ± 9.3	[a] 0.042 [b] 0.008 [c] 0.626
	End of Phase II	26	33.6 ± 8.0	26	32.1 ± 9.6	25	31.5 ± 7.0	[a] 0.536 [b] 0.333 [c] 0.826

Note: *p-value is calculated using unpaired t-test. ([a]- Arm-A vs Arm-B, [b]- Arm-A vs Arm-C, [c]- Arm-B vs Arm-C).*

Summary:

Glycaemic parameters Comparing between groups, Mean MAGE (mg/dL) of subjects at pre-treatment was 80.6 ± 19.2, 86.1 ± 21.3 and 87.4 ± 19.0 respectively in Arm-A, Arm-B and Arm-C. At End of Phase I MAGE (mg/dl) were 61.2 ± 21.3, 66.7 ± 20.9 and 57.4 ± 21.0in Arm-A, Arm-B and Arm-C. At End of Phase II MAGE (mg/dL) were 58.0 ± 13.8, 65.5 ± 26.2 and 59.9 ± 17.3 in Arm-A, Arm-B and Arm-C. Mean LAGE (mg/dL) during Pre-treatment 122.8 ± 31.9, 132.4 ± 31.7 and 136.3 ± 28.3 in Arm-A, Arm-B and Arm-C. At End phase I Mean LAGE (mg/dL) 96.8 ± 30.7, 101.2 ± 36.7 and 84.9 ± 28.1 in Arm-A, Arm-B and Arm-C. At End phase II Mean LAGE (mg/dL)

DocUUID : b897a93b-83ba-4de2-a309-31160c3ed1c4

89.5 ± 21.5, 103.3 ± 46.6and 89.6 ± 33.4 in Arm-A, Arm-B and Arm-C. Mean SD during Pre-treatment 54.0 ± 12.8, 57.3 ± 14.2 and 57.7 ± 11.0 in Arm-A, Arm-B and Arm-C. At End phase I Mean SD 44.5 ± 14.8, 44.5 ± 14.9 and 40.2 ± 13.2 in Arm-A, Arm-B and Arm-C. At End phase II Mean SD 40.8 ± 11.2, 46.1 ± 18.9 and 41.1 ± 12.9 in Arm-A, Arm-B and Arm-C. Mean CV (%) during Pre-treatment 35.7 ± 10.4, 33.6 ± 8.4 and 33.1 ± 6.4 in Arm-A, Arm-B and Arm-C. At End phase I Mean CV (%) 36.0 ± 10.6, 30.0 ± 11.1 and 28.6 ± 9.3 in Arm-A, Arm-B and Arm-C. At End phase II Mean CV (%) 33.6 ± 8.0, 32.1 ± 9.6 and 31.5 ± 7.0 in Arm-A, Arm-B and Arm-C.

Table-11 5: Summary of Post-Prandial Excursions (MPPGE, PP-1hr, PP-2hr) during different phases of the study with baseline comparison

Parameters	Visits	Arm-A (Teneligliptin 20 mg + Dapagliflozin 10 mg)			Arm-B (Sitagliptin 100 mg + Dapagliflozin 10 mg)			Arm-C (Empagliflozin 25 mg + Linagliptin 5 mg)		
		n	Mean ± SD	p-value	N	Mean ± SD	p-value	n	Mean ± SD	p-value
MPPGE	Pre-treatment	30	159.6 ± 49.6	-	28	172.3 ± 46.5	-	29	180.3 ± 47.6	-
	End of Phase I	29	135.8 ± 51.2	0.035	28	158.0 ± 53.6	0.182	27	146.2 ± 35.4	0.001
	End of Phase II	25	125.0 ± 33.5	<0.001	26	149.0 ± 46.8	0.01	25	135.7 ± 33.2	<0.001
PP-1hr	Pre-treatment	30	158.0 ± 51.0	-	28	172.1 ± 44.6	-	29	176.0 ± 47.7	-
	End of Phase I	29	130.8 ± 53.0	0.016	28	152.4 ± 55.2	0.088	25	142.6 ± 37.0	<0.001
	End of Phase II	25	132.5 ± 40.2	0.021	24	138.8 ± 41.4	0.001	25	136.7 ± 38.7	0.002
PP-2hr	Pre-treatment	30	160.4 ± 51.7	-	28	169.9 ± 47.0	-	29	176.2 ± 49.6	-
	End of Phase I	27	130.3 ± 41.6	0.008	27	161.4 ± 57.2	0.591	23	150.8 ± 44.5	0.036
	End of Phase II	23	133.3 ± 43.6	0.013	22	155.5 ± 65.3	0.135	24	128.8 ± 40.4	0.001

Post-Prandial Excursions baseline comparison, Mean MPPGE of subjects at pre-treatment was 159.6 ± 49.6, 172.3 ± 46.5 and 180.3 ± 47.6 respectively in Arm-A, Arm-B and Arm-C. At End of Phase I MPPGE were 135.8 ± 51.2, 158.0 ± 53.6 and 146.2 ± 35.4 in Arm-A, Arm-B and Arm-C. At End of Phase II MPPGE were 125.0 ± 33.5, 149.0 ± 46.8 and 135.7 ± 33.2 in Arm-A, Arm-B and Arm-C. Mean PP 1 hr during Pre-treatment 158.0 ± 51.0, 172.1 ± 44.6 and 176.0 ± 47.7 in Arm-A, Arm-B and Arm-C. At End phase I Mean pp 1 hr 130.8 ± 53.0, 152.4 ± 55.2 and 142.6 ± 37.0 in Arm-A, Arm-B and Arm-C. At End phase II Mean PP 1 hr 132.5 ± 40.2, 138.8 ± 41.4 and 136.7 ± 38.7 in Arm-A, Arm-B and Arm-C. Mean PP 2 hr during Pre-treatment 160.4 ± 51.7, 169.9 ± 47.0 and 176.2 ± 49.6 in Arm-A, Arm-B and Arm-C. At End phase I Mean pp 2 hr 130.3 ± 41.6, 161.4 ± 57.2 and 150.8 ± 44.5 in Arm-A, Arm-B and Arm-C. At End phase II Mean PP 2 hr 133.3 ± 43.6, 155.5 ± 65.3 and 128.8 ± 40.4 in Arm-A, Arm-B and Arm-C.

DocUUID : b897a93b-83ba-4de2-a309-31160c3ed1c4

Table-11 6: Summary of Post-Prandial Excursions (MPPGE, PP-1hr, PP-2hr) during different phases of the study comparing between the group

Parameters	Visits	Arm-A (Teneligliptin 20 mg + Dapagliflozin 10 mg)		Arm-B (Sitagliptin 100 mg + Dapagliflozin 10 mg)		Arm-C (Empagliflozin 25 mg + Linagliptin 5 mg)		p-value
		n	Mean ± SD	n	Mean ± SD	n	Mean ± SD	
MPPGE	Pre-treatment	30	159.6 ± 49.6	28	172.3 ± 46.5	29	180.3 ± 47.6	[a] 0.316 [b] 0.106 [c] 0.523
	End of Phase I	29	135.8 ± 51.2	28	158.0 ± 53.6	27	146.2 ± 35.4	[a] 0.116 [b] 0.382 [c] 0.336
	End of Phase II	25	125.0 ± 33.5	26	149.0 ± 46.8	25	135.7 ± 33.2	[a] 0.040 [b] 0.261 [c] 0.245
PP-1hr	Pre-treatment	30	158.0 ± 51.0	28	172.1 ± 44.6	29	176.0 ± 47.7	[a] 0.267 [b] 0.166 [c] 0.747
	End of Phase I	29	130.8 ± 53.0	28	152.4 ± 55.2	25	142.6 ± 37.0	[a] 0.138 [b] 0.342 [c] 0.447
	End of Phase II	25	132.5 ± 40.2	24	138.8 ± 41.4	25	136.7 ± 38.7	[a] 0.588 [b] 0.704 [c] 0.856
PP-2hr	Pre-treatment	30	160.4 ± 51.7	28	169.9 ± 47.0	29	176.2 ± 49.6	[a] 0.468 [b] 0.236 [c] 0.623
	End of Phase I	27	130.3 ± 41.6	27	161.4 ± 57.2	23	150.8 ± 44.5	[a] 0.027 [b] 0.102 [c] 0.464
	End of Phase II	23	133.3 ± 43.6	22	155.5 ± 65.3	24	128.8 ± 40.4	[a] 0.189 [b] 0.716 [c] 0.108

Note: *p-value is calculated using unpaired t-test. ([a]- Arm-A vs Arm-B, [b]- Arm-A vs Arm-C, [c]- Arm-B vs Arm-C).*

Summary:

Post-Prandial Excursions between group comparison, Mean MPPGE of subjects at pre-treatment was 159.6 ± 49.6, 172.3 ± 46.5 and 180.3 ± 47.6 respectively in Arm-A, Arm-B and Arm-C. At End of Phase I MPPGE were 135.8 ± 51.2, 158.0 ± 53.6 and 146.2 ± 35.4 in Arm-A, Arm-B and Arm-C. At End of Phase II MPPGE were 125.0 ± 33.5, 149.0 ± 46.8 and 135.7 ± 33.2 in Arm-A, Arm-B and Arm-C. Mean PP 1 hr during Pre-treatment 158.0 ± 51.0, 172.1 ± 44.6 and 176.0 ± 47.7 in Arm-A, Arm-B and Arm-C. At End phase I Mean pp 1 hr 130.8 ± 53.0, 152.4 ± 55.2 and 142.6 ± 37.0 in Arm-A, Arm-B and Arm-C. At End phase II Mean PP 1 hr 132.5 ± 40.2, 138.8 ± 41.4 and 136.7 ± 38.7 in Arm-A, Arm-B and Arm-C. Mean PP 2 hr during Pre-treatment 160.4 ± 51.7, 169.9 ± 47.0 and 176.2 ± 49.6 in Arm-A, Arm-B and Arm-C. At End phase I Mean pp 2 hr 130.3 ± 41.6, 161.4 ± 57.2 and 150.8 ± 44.5 in Arm-A, Arm-B and Arm-C. At End phase II Mean PP 2 hr 133.3 ± 43.6, 155.5 ± 65.3 and 128.8 ± 40.4 in Arm-A, Arm-B and Arm-C.

DocUUID : b897a93b-83ba-4de2-a309-31160c3ed1c4

Table-11 7: Summary of Post-Prandial Excursions (MPPGE, PP-1hr, PP-2hr) during different phases of the study with baseline comparison at Breakfast

Parameters	Visits	Arm-A (Teneligliptin 20 mg + Dapagliflozin 10 mg)			Arm-B (Sitagliptin 100 mg + Dapagliflozin 10 mg)			Arm-C (Empagliflozin 25 mg + Linagliptin 5 mg)		
		n	Mean ± SD	p-value	n	Mean ± SD	p-value	n	Mean ± SD	p-value
MPPGE	Pre-treatment	30	157.9 ± 54.9	-	28	171.2 ± 50.4	-	29	178.4 ± 53.4	-
	End of Phase I	29	130.6 ± 57.0	0.011	28	157.9 ± 47.8	0.181	27	145.9 ± 35.4	0.001
	End of Phase II	25	125.3 ± 37.9	0.001	26	145.0 ± 45.6	0.006	25	129.0 ± 36.1	<0.001
PP-1hr	Pre-treatment	30	152.9 ± 57.9	-	28	167.1 ± 50.9	-	29	168.9 ± 53.3	-
	End of Phase I	29	129.4 ± 63.1	0.039	28	150.8 ± 52.1	0.147	25	143.1 ± 43.1	0.002
	End of Phase II	25	130.8 ± 45.2	0.097	24	139.5 ± 40.6	0.009	25	131.2 ± 36.3	0.001
PP-2hr	Pre-treatment	30	170.7 ± 60.1	-	28	180.2 ± 54.0	-	29	178.3 ± 59.3	-
	End of Phase I	27	134.3 ± 57.5	0.002	27	164.7 ± 58.6	0.265	23	149.4 ± 44.7	0.017
	End of Phase II	23	129.1 ± 48.0	0.001	22	159.7 ± 66.3	0.034	24	122.2 ± 41.7	<0.001

Note: *p-value is calculated using paired t-test.*

Post-Prandial Excursions with baseline comparison at breakfast, Mean MPPGE of subjects at pre-treatment was 157.9 ± 54.9, 171.2 ± 50.4 and 178.4 ± 53.4 respectively in Arm-A, Arm-B and Arm-C. At End of Phase I MPPGE were 130.6 ± 57.0, 157.9 ± 47.8 and 145.9 ± 35.4 in Arm-A, Arm-B and Arm-C. At End of Phase II MPPGE were 125.3 ± 37.9, 145.0 ± 45.6 and 129.0 ± 36.1in Arm-A, Arm- B and Arm-C. Mean PP 1 hr during Pre-treatment 152.9 ± 57.9, 167.1 ± 50.9 and 168.9 ± 53.3 in Arm-A, Arm-B and Arm-C. At End phase I Mean pp 1 hr 129.4 ± 63.1, 150.8 ± 52.1 and 143.1 ± 43.1 in Arm-A, Arm-B and Arm-C. At End phase II Mean PP 1 hr 130.8 ± 45.2, 139.5 ± 40.6 and 131.2 ± 36.3 in Arm-A, Arm-B and Arm-C. Mean PP 2 hr during Pre-treatment 170.7 ± 60.1, 180.2 ± 54.0 and 178.3 ± 59.3 in Arm-A, Arm-B and Arm-C. At End phase I Mean pp 2 hr 134.3 ± 57.5, 164.7 ± 58.6 and 149.4 ± 44.7 in Arm-A, Arm-B and Arm-C. At End phase II Mean PP 2 hr 129.1 ± 48.0, 159.7 ± 66.3 and 122.2 ± 41.7 in Arm-A, Arm-B and Arm-C.

Table-11 8: Summary of Post-Prandial Excursions (MPPGE, PP-1hr, PP-2hr) during different phases of the study comparing between the group at Breakfast

Parameters	Visits	Arm-A (Teneligliptin 20 mg + Dapagliflozin 10 mg)		Arm-B (Sitagliptin 100 mg + Dapagliflozin 10 mg)		Arm-C (Empagliflozin 25 mg + Linagliptin 5 mg)		p-value
		n	Mean ± SD	n	Mean ± SD	n	Mean ± SD	
MPPGE	Pre-treatment	30	157.9 ± 54.9	28	171.2 ± 50.4	29	178.4 ± 53.4	[a] 0.342 [b] 0.152 [c] 0.604
	End of Phase I	29	130.6 ± 57.0	28	157.9 ± 47.8	27	145.9 ± 35.4	[a] 0.055 [b] 0.232 [c] 0.293

DocUUID : b897a93b-83ba-4de2-a309-31160c3ed1c4

Parameters	Visits	Arm-A (Teneligliptin 20 mg + Dapagliflozin 10 mg)		Arm-B (Sitagliptin 100 mg + Dapagliflozin 10 mg)		Arm-C (Empagliflozin 25 mg + Linagliptin 5 mg)		p-value
		n	Mean ± SD	n	Mean ± SD	n	Mean ± SD	
	End of Phase II	25	125.3 ± 37.9	26	145.0 ± 45.6	25	129.0 ± 36.1	[a] 0.098 [b] 0.725 [c] 0.169
PP-1hr	Pre-treatment	30	152.9 ± 57.9	28	167.1 ± 50.9	29	168.9 ± 53.3	[a] 0.323 [b] 0.271 [c] 0.894
	End of Phase I	29	129.4 ± 63.1	28	150.8 ± 52.1	25	143.1 ± 43.1	[a] 0.169 [b] 0.351 [c] 0.562
	End of Phase II	25	130.8 ± 45.2	24	139.5 ± 40.6	25	131.2 ± 36.3	[a] 0.479 [b] 0.973 [c] 0.453
PP-2hr	Pre-treatment	30	170.7 ± 60.1	28	180.2 ± 54.0	29	178.3 ± 59.3	[a] 0.530 [b] 0.627 [c] 0.902
	End of Phase I	27	134.3 ± 57.5	27	164.7 ± 58.6	23	149.4 ± 44.7	[a] 0.060 [b] 0.302 [c] 0.303
	End of Phase II	23	129.1 ± 48.0	22	159.7 ± 66.3	24	122.2 ± 41.7	[a] 0.085 [b] 0.601 [c] 0.029

Note: *p-value is calculated using unpaired t-test. ([a]- Arm-A vs Arm-B, [b]- Arm-A vs Arm-C, [c]- Arm-B vs Arm-C).*

Summary:

Post-Prandial Excursions with between group comparison at breakfast, Mean MPPGE of subjects at pre-treatment was 157.9 ± 54.9, 171.2 ± 50.4 and 178.4 ± 53.4 respectively in Arm-A, Arm-B and Arm-C. At End of Phase I MPPGE were 130.6 ± 57.0, 157.9 ± 47.8 and 145.9 ± 35.4 in Arm-A, Arm-B and Arm-C. At End of Phase II MPPGE were 125.3 ± 37.9, 145.0 ± 45.6 and 129.0 ± 36.1in Arm-A, Arm- B and Arm-C. Mean PP 1 hr during Pre-treatment 152.9 ± 57.9, 167.1 ± 50.9 and 168.9 ± 53.3 in Arm-A, Arm-B and Arm-C. At End phase I Mean pp 1 hr 129.4 ± 63.1, 150.8 ± 52.1 and 143.1 ± 43.1 in Arm-A, Arm-B and Arm-C. At End phase II Mean PP 1 hr 130.8 ± 45.2, 139.5 ± 40.6 and 131.2 ± 36.3 in Arm-A, Arm-B and Arm-C. Mean PP 2 hr during Pre-treatment 170.7 ± 60.1, 180.2 ± 54.0 and 178.3 ± 59.3 in Arm-A, Arm-B and Arm-C. At End phase I Mean pp 2 hr 134.3 ± 57.5, 164.7 ± 58.6 and 149.4 ± 44.7 in Arm-A, Arm-B and Arm-C. At End phase II Mean PP 2 hr 129.1 ± 48.0, 159.7 ± 66.3 and 122.2 ± 41.7 in Arm-A, Arm-B and Arm-C.

DocUUID : b897a93b-83ba-4de2-a309-31160c3ed1c4

Table-11 9: Summary of Post-Prandial Excursions (MPPGE, PP-1hr, PP-2hr) during different phases of the study with baseline comparison at Lunch

Parameters	Visits	Arm-A (Teneligliptin 20 mg + Dapagliflozin 10 mg)			Arm-B (Sitagliptin 100 mg + Dapagliflozin 10 mg)			Arm-C (Empagliflozin 25 mg + Linagliptin 5 mg)		
		n	Mean ± SD	p-value	N	Mean ± SD	p-value	n	Mean ± SD	p-value
MPPGE	Pre-treatment	30	161.1 ± 50.1	-	28	177.0 ± 57.1	-	29	186.5 ± 47.9	-
	End of Phase I *	17	137.9 ± 47.0	0.286	13	154.6 ± 76.8	0.832	12	162.5 ± 43.6	0.115
	End of Phase II #	15	126.1 ± 37.1	0.007	10	157.5 ± 46.8	0.265	15	159.6 ± 35.3	0.068
PP-1hr	Pre-treatment	30	160.6 ± 52.8	-	28	182.6 ± 66.6	-	29	181.4 ± 54.8	-
	End of Phase I *	14	138.6 ± 60.3	0.269	10	142.8 ± 83.2	0.586	9	156.4 ± 44.3	0.419
	End of Phase II #	12	133.7 ± 42.4	0.044	6	136.5 ± 60.8	0.562	11	169.8 ± 47.3	0.958
PP-2hr	Pre-treatment	30	157.2 ± 58.9	-	28	170.0 ± 66.2	-	29	181.6 ± 48.5	-
	End of Phase I *	12	119.0 ± 18.7	0.127	7	116.6 ± 43.7	0.080	7	145.6 ± 44.2	0.179
	End of Phase II #	11	136.6 ± 47.0	0.176	6	127.0 ± 36.3	0.214	10	163.5 ± 48.4	0.503

Note: *p-value is calculated using paired t-test.*

**At End of Phase I*

- *MPPGE was obtained for 17,13 and 12 subjects in Arm-A, Arm-B and Arm-C respectively as the CGM device was removed for remaining subjects before Meal 2.*
- *PP-1hr was obtained for 14,10 and 9 subjects in Arm-A, Arm-B and Arm-C respectively as the CGM device was removed for remaining subjects before or within 1 hour of Meal 2.*
- *PP-2hr was obtained for 12,7 and 7 subjects in Arm-A, Arm-B and Arm-C respectively as the CGM device was removed for remaining subjects before or within 2 hour of Meal 2.*

#At End of Phase II

- *MPPGE was obtained for 15,10 and 15 subjects in Arm-A, Arm-B and Arm-C respectively as the CGM device was removed for remaining subjects before Meal 2.*
- *PP-1hr was obtained for 12, 6 and 11 subjects in Arm-A, Arm-B and Arm-C respectively as the CGM device was removed for remaining subjects before or within 1 hour of Meal 2.*
- *PP-2hr was obtained for 11,6 and 10 subjects in Arm-A, Arm-B and Arm-C respectively as the CGM device was removed for remaining subjects before or within 2 hour of Meal 2.*

Summary:

Post-Prandial Excursions with baseline comparison at Lunch, Mean MPPGE of subjects at pre-treatment was 161.1 ± 50.1, 177.0 ± 57.1 and 186.5 ± 47.9 respectively in Arm-A, Arm-B and Arm-C. At End of Phase I MPPGE were 137.9 ± 47.0, 154.6 ± 76.8 and 162.5 ± 43.6 in Arm-A, Arm-B and Arm-C. At End of Phase II MPPGE were 126.1 ± 37.1, 157.5 ± 46.8 and 159.6 ± 35.3 in Arm-A, Arm- B and Arm-C. Mean PP 1 hr during Pre-treatment 160.6 ± 52.8, 182.6 ± 66.6 and 181.4 ± 54.8 in Arm-A, Arm-B and Arm-C. At End phase I Mean pp 1 hr 138.6 ± 60.3, 142.8 ± 83.2 and 156.4 ± 44.3 in Arm-A, Arm-B and Arm-C. At End phase II Mean PP 1 hr 133.7 ± 42.4, 136.5 ± 60.8 and 169.8 ± 47.3 in Arm-A, Arm-B and Arm-C. Mean PP 2 hr during Pre-treatment 157.2 ± 58.9, 170.0 ± 66.2 and 181.6 ± 48.5 in Arm-A, Arm-B and Arm-C. At End phase I Mean pp 2 hr 119.0 ± 18.7, 116.6 ± 43.7 and 145.6 ± 44.2 in Arm-A, Arm-B and Arm-C. At End phase II Mean PP 2 hr 136.6 ± 47.0, 127.0 ± 36.3 and 163.5 ± 48.4 in Arm-A, Arm-B and Arm-C.

DocUUID : b897a93b-83ba-4de2-a309-31160c3ed1c4

Table-11 10: Summary of Post-Prandial Excursions (MPPGE, PP-1hr, PP-2hr) during different phases of the study comparing between the group at Lunch

Parameters	Visits	Arm-A (Teneligliptin 20 mg + Dapagliflozin 10 mg)		Arm-B (Sitagliptin 100 mg + Dapagliflozin 10 mg)		Arm-C (Empagliflozin 25 mg + Linagliptin 5 mg)		p-value
		n	Mean ± SD	n	Mean ± SD	n	Mean ± SD	
MPPGE	Pre-treatment	30	161.1 ± 50.1	28	177.0 ± 57.1	29	186.5 ± 47.9	[a] 0.265 [b] 0.051 [c] 0.503
	End of Phase I *	17	137.9 ± 47.0	13	154.6 ± 76.8	12	162.5 ± 43.6	[a] 0.499 [b] 0.161 [c] 0.753
	End of Phase II #	15	126.1 ± 37.1	10	157.5 ± 46.8	15	159.6 ± 35.3	[a] 0.093 [b] 0.017 [c] 0.908
PP-1hr	Pre-treatment	30	160.6 ± 52.8	28	182.6 ± 66.6	29	181.4 ± 54.8	[a] 0.171 [b] 0.144 [c] 0.940
	End of Phase I *	14	138.6 ± 60.3	10	142.8 ± 83.2	9	156.4 ± 44.3	[a] 0.893 [b] 0.423 [c] 0.658
	End of Phase II #	12	133.7 ± 42.4	6	136.5 ± 60.8	11	169.8 ± 47.3	[a] 0.921 [b] 0.069 [c] 0.276
PP-2hr	Pre-treatment	30	157.2 ± 58.9	28	170.0 ± 66.2	29	181.6 ± 48.5	[a] 0.438 [b] 0.087 [c] 0.456
	End of Phase I *	12	119.0 ± 18.7	7	116.6 ± 43.7	7	145.6 ± 44.2	[a] 0.893 [b] 0.172 [c] 0.240
	End of Phase II #	11	136.6 ± 47.0	6	127.0 ± 36.3	10	163.5 ± 48.4	[a] 0.646 [b] 0.213 [c] 0.110

Note: *p-value is calculated using unpaired t-test. ([a]- Arm-A vs Arm-B, [b]- Arm-A vs Arm-C, [c]- Arm-B vs Arm-C).*

**At End of Phase I*
- *MPPGE was obtained for 17,13 and 12 subjects in Arm-A, Arm-B and Arm-C respectively as the CGM device was removed for remaining subjects before Meal 2.*
- *PP-1hr was obtained for 14,10 and 9 subjects in Arm-A, Arm-B and Arm-C respectively as the CGM device was removed for remaining subjects before or within 1 hour of Meal 2.*
- *PP-2hr was obtained for 12,7 and 7 subjects in Arm-A, Arm-B and Arm-C respectively as the CGM device was removed for remaining subjects before or within 2 hour of Meal 2.*

#At End of Phase II
- *MPPGE was obtained for 15,10 and 15 subjects in Arm-A, Arm-B and Arm-C respectively as the CGM device was removed for remaining subjects before Meal 2.*
- *PP-1hr was obtained for 12, 6 and 11 subjects in Arm-A, Arm-B and Arm-C respectively as the CGM device was removed for remaining subjects before or within 1 hour of Meal 2.*
- *PP-2hr was obtained for 11,6 and 10 subjects in Arm-A, Arm-B and Arm-C respectively as the CGM device was removed for remaining subjects before or within 2 hour of Meal 2.*

Summary:

Post-Prandial Excursions with between group comparison at Lunch, Mean MPPGE of subjects at pre-treatment was 161.1 ± 50.1, 177.0 ± 57.1 and 186.5 ± 47.9 respectively in Arm-A, Arm-B and Arm-C. At End of Phase I MPPGE were 137.9 ± 47.0, 154.6 ± 76.8 and 162.5 ± 43.6 in Arm-A,

DocUUID : b897a93b-83ba-4de2-a309-31160c3ed1c4

Arm-B and Arm-C. At End of Phase II MPPGE were 126.1 ± 37.1, 157.5 ± 46.8 and 159.6 ± 35.3 in Arm-A, Arm- B and Arm-C. Mean PP 1 hr during Pre-treatment 160.6 ± 52.8, 182.6 ± 66.6 and 181.4 ± 54.8 in Arm-A, Arm-B and Arm-C. At End phase I Mean pp 1 hr 138.6 ± 60.3, 142.8 ± 83.2 and 156.4 ± 44.3 in Arm-A, Arm-B and Arm-C. At End phase II Mean PP 1 hr 133.7 ± 42.4, 136.5 ± 60.8 and 169.8 ± 47.3 in Arm-A, Arm-B and Arm-C. Mean PP 2 hr during Pre-treatment 157.2 ± 58.9, 170.0 ± 66.2 and 181.6 ± 48.5 in Arm-A, Arm-B and Arm-C. At End phase I Mean pp 2 hr 119.0 ± 18.7, 116.6 ± 43.7 and 145.6 ± 44.2 in Arm-A, Arm-B and Arm-C. At End phase II Mean PP 2 hr 136.6 ± 47.0, 127.0 ± 36.3 and 163.5 ± 48.4 in Arm-A, Arm-B and Arm-C.

Table-11 11: Summary of Post-Prandial Excursions (MPPGE, PP-1hr, PP-2hr) during different phases of the study with baseline comparison at Dinner

Parameters	Visits	Arm-A (Teneligliptin 20 mg + Dapagliflozin 10 mg)			Arm-B (Sitagliptin 100 mg + Dapagliflozin 10 mg)			Arm-C (Empagliflozin 25 mg + Linagliptin 5 mg)		
		n	Mean ± SD	p-value	N	Mean ± SD	p-value	n	Mean ± SD	p-value
MPPGE	Pre-treatment	30	161.5 ± 53.9	-	28	170.1 ± 46.2	-	29	176.8 ± 54.8	-
	End of Phase I *	7	109.7 ± 40.0	0.131	4	143.2 ± 88.3	0.772	3	151.6 ± 33.9	0.746
	End of Phase II #	6	113.1 ± 40.2	0.056	3	145.8 ± 67.0	0.866	1	135.0 ± 135.0	-
PP-1hr	Pre-treatment	30	162.5 ± 56.8	-	28	169.1 ± 45.3	-	29	176.4 ± 53.2	-
	End of Phase I *	6	101.8 ± 32.1	0.01	3	155.0 ± 107.6	0.834	3	128.3 ± 22.2	0.092
	End of Phase II #	4	98.2 ± 38.5	0.025	1	195.0 ± 195.0	-	0	-	-
PP-2hr	Pre-treatment	30	157.8 ± 55.4	-	28	164.4 ± 49.0	-	29	170.5 ± 59.8	-
	End of Phase I *	3	94.7 ± 50.2	0.123	2	95.0 ± 49.5	0.597	2	202.5 ± 53.0	0.506
	End of Phase II #	1	72.0 ± 72.0	-	1	211.0 ± 211.0	-	0	-	-

Note: *p-value is calculated using paired t-test.*

*At End of Phase I

- *MPPGE was obtained for 7, 4 and 3 subjects in Arm-A, Arm-B and Arm-C respectively as the CGM device was removed for remaining subjects before Meal 3.*
- *PP-1hr was obtained for 6, 3 and 3 subjects in Arm-A, Arm-B and Arm-C respectively as the CGM device was removed for remaining subjects before or within 1 hour of Meal 3.*
- *PP-2hr was obtained for 3,2 and 2 subjects in Arm-A, Arm-B and Arm-C respectively as the CGM device was removed for remaining subjects before or within 2 hour of Meal 3.*

#At End of Phase II

- *MPPGE was obtained for 6, 3 and 1 subjects in Arm-A, Arm-B and Arm-C respectively as the CGM device was removed for remaining subjects before Meal 3.*
- *PP-1hr was obtained for 4, 1 and 0 subjects in Arm-A, Arm-B and Arm-C respectively as the CGM device was removed for remaining subjects before or within 1 hour of Meal 3.*
- *PP-2hr was obtained for 1, 1 and 0 subjects in Arm-A, Arm-B and Arm-C respectively as the CGM device was removed for remaining subjects before or within 2 hour of Meal 3*

Summary:

Post-Prandial Excursions with baseline comparison at dinner, Mean MPPGE of subjects at pre-treatment was 161.5 ± 53.9, 170.1 ± 46.2 and 176.8 ± 54.8 respectively in Arm-A, Arm-B and Arm-

DocUUID : b897a93b-83ba-4de2-a309-31160c3ed1c4

C. At End of Phase I MPPGE were 109.7 ± 40.0, 143.2 ± 88.3 and 151.6 ± 33.9 in Arm-A, Arm-B and Arm-C. At End of Phase II MPPGE were 113.1 ± 40.2, 145.8 ± 67.0 and 135.0 ± 135.0 in Arm-A, Arm- B and Arm-C. Mean PP 1 hr during Pre-treatment 162.5 ± 56.8, 169.1 ± 45.3 and 176.4 ± 53.2 in Arm-A, Arm-B and Arm-C. At End phase I Mean pp 1 hr 101.8 ± 32.1, 155.0 ± 107.6 and 128.3 ± 22.2 in Arm-A, Arm-B and Arm-C. At End phase II Mean PP 1 hr 98.2 ± 38.5 and 195.0 ± 195.0 in Arm-A and Arm-B. Mean PP 2 hr during Pre-treatment 157.8 ± 55.4, 164.4 ± 49.0 and 170.5 ± 59.8 in Arm-A, Arm-B and Arm-C. At End phase I Mean pp 2 hr 94.7 ± 50.2, 95.0 ± 49.5 and 202.5 ± 53.0 in Arm-A, Arm-B and Arm-C. At End phase II Mean PP 2 hr 72.0 ± 72.0 and 211.0 ± 211.0 in Arm-A and Arm-B.

Table-11 12: Summary of Post-Prandial Excursions (MPPGE, PP-1hr, PP-2hr) during different phases of the study comparing between the group at Dinner

Parameters	Visits	Arm-A (Teneligliptin 20 mg + Dapagliflozin 10 mg)		Arm-B (Sitagliptin 100 mg + Dapagliflozin 10 mg)		Arm-C (Empagliflozin 25 mg + Linagliptin 5 mg)		p-value
		n	Mean ± SD	n	Mean ± SD	n	Mean ± SD	
MPPGE	Pre-treatment	30	161.5 ± 53.9	28	170.1 ± 46.2	29	176.8 ± 54.8	[a] 0.52 [b] 0.284 [c] 0.616
	End of Phase I *	7	109.7 ± 40.0	4	143.2 ± 88.3	3	151.6 ± 33.9	[a] 0.516 [b] 0.157 [c]] 0.87
	End of Phase II #	6	113.1 ± 40.2	3	145.8 ± 67.0	1	135.0 ± 135.0	[a] 0.498 [b] - [c] -
PP-1hr	Pre-treatment	30	162.5 ± 56.8	28	169.1 ± 45.3	29	176.4 ± 53.2	[a] 0.626 [b] 0.336 [c] 0.579
	End of Phase I *	6	101.8 ± 32.1	3	155.0 ± 107.6	3	128.3 ± 22.2	[a] 0.484 [b] 0.200 [c] 0.712
	End of Phase II #	4	98.2 ± 38.5	1	195.0 ± 195.0	0	-	[a] - [b] - [c] -
PP-2hr	Pre-treatment	30	157.8 ± 55.4	28	164.4 ± 49.0	29	170.5 ± 59.8	[a] 0.631 [b] 0.402 [c] 0.676
	End of Phase I *	3	94.7 ± 50.2	2	95.0 ± 49.5	2	202.5 ± 53.0	[a] 0.995 [b] 0.141 [c] 0.172
	End of Phase II #	1	72.0 ± 72.0	1	211.0 ± 211.0	0	-	[a] - [b] - [c] -

Note: *p-value is calculated using unpaired t-test. ([a]- Arm-A vs Arm-B, [b]- Arm-A vs Arm-C, [c]- Arm-B vs Arm-C).*

**At End of Phase I*

- *MPPGE was obtained for 7, 4 and 3 subjects in Arm-A, Arm-B and Arm-C respectively as the CGM device was removed for remaining subjects before Meal 3.*
- *PP-1hr was obtained for 6, 3 and 3 subjects in Arm-A, Arm-B and Arm-C respectively as the CGM device was removed for remaining subjects before or within 1 hour of Meal 3.*
- *PP-2hr was obtained for 3,2 and 2 subjects in Arm-A, Arm-B and Arm-C respectively as the CGM device was removed for remaining subjects before or within 2 hour of Meal 3.*

#At End of Phase II

DocUUID : b897a93b-83ba-4de2-a309-31160c3ed1c4

- *MPPGE was obtained for 6, 3 and 1 subjects in Arm-A, Arm-B and Arm-C respectively as the CGM device was removed for remaining subjects before Meal 3.*
- *PP-1hr was obtained for 4, 1 and 0 subjects in Arm-A, Arm-B and Arm-C respectively as the CGM device was removed for remaining subjects before or within 1 hour of Meal 3.*
- *PP-2hr was obtained for 1, 1 and 0 subjects in Arm-A, Arm-B and Arm-C respectively as the CGM device was removed for remaining subjects before or within 2 hour of Meal 3*

Summary:

Post-Prandial Excursions with between group comparison at dinner, Mean MPPGE of subjects at pre-treatment was 161.5 ± 53.9, 170.1 ± 46.2 and 176.8 ± 54.8 respectively in Arm-A, Arm-B and Arm-C. At End of Phase I MPPGE were 109.7 ± 40.0, 143.2 ± 88.3 and 151.6 ± 33.9 in Arm-A, Arm-B and Arm-C. At End of Phase II MPPGE were 113.1 ± 40.2, 145.8 ± 67.0 and 135.0 ± 135.0 in Arm-A, Arm- B and Arm-C. Mean PP 1 hr during Pre-treatment 162.5 ± 56.8, 169.1 ± 45.3 and 176.4 ± 53.2 in Arm-A, Arm-B and Arm-C. At End phase I Mean pp 1 hr 101.8 ± 32.1, 155.0 ± 107.6 and 128.3 ± 22.2 in Arm-A, Arm-B and Arm-C. At End phase II Mean PP 1 hr 98.2 ± 38.5 and 195.0 ± 195.0 in Arm-A and Arm-B. Mean PP 2 hr during Pre-treatment 157.8 ± 55.4, 164.4 ± 49.0 and 170.5 ± 59.8 in Arm-A, Arm-B and Arm-C. At End phase I Mean pp 2 hr 94.7 ± 50.2, 95.0 ± 49.5 and 202.5 ± 53.0 in Arm-A, Arm-B and Arm-C. At End phase II Mean PP 2 hr 72.0 ± 72.0 and 211.0 ± 211.0 in Arm-A and Arm-B.

Table-11 13: Summary Glycemic results (FPG, PPG, HBA1c)

Parameter	Visits	Arm-A (Teneligliptin 20 mg + Dapagliflozin 10 mg)			Arm-B (Sitagliptin 100 mg + Dapagliflozin 10 mg)			Arm-C (Empagliflozin 25 mg + Linagliptin 5 mg)		
		n	Mean ± SD	p-value	n	Mean ± SD	p-value	n	Mean ± SD	p-value
**HbA1c**	Visit 1 (Day -3 to Day 0)	30	8.47 ± 0.72	-	30	8.44 ± 0.79	-	30	8.81 ± 0.78	-
	Visit 6: (Day 49)	29	7.14 ± 1.09	<0.001	28	7.85 ± 1.13	0.011	26	7.57 ± 1.21	<0.001
	Visit 7: (Day 90)	29	6.85 ± 0.98	<0.001	28	7.12 ± 0.90	<0.001	25	7.04 ± 0.92	<0.001
**FPG**	Visit 1 (Day -3 to Day 0)	30	144.00 ± 30.57	-	30	147.28 ± 45.68	-	30	147.29 ± 38.65	-
	Visit 6: (Day 49)	25	118.22 ± 19.46	<0.001	22	123.00 ± 23.96	0.064	19	115.20 ± 14.06	0.004
	Visit 7: (Day 90)	29	104.18 ± 13.89	<0.001	28	113.82 ± 20.25	0.001	25	111.71 ± 18.54	<0.001
**PPG**	Visit 1 (Day -3 to Day 0)	30	194.02 ± 47.17	-	30	212.14 ± 77.62	-	30	195.80 ± 44.66	-
	Visit 6: (Day 49)	25	158.12 ± 31.68	0.001	28	165.19 ± 34.68	0.007	26	160.02 ± 40.88	0.005
	Visit 7: (Day 90)	29	155.19 ± 40.76	0.003	28	145.06 ± 29.97	<0.001	25	160.30 ± 43.93	0.012

Note: *N-Number of Subjects in Group; n-Number of samples in Group*; *p-value will be calculated using paired t-test.*

Summary:

The mean HbA1c (%) of the subjects was 8.47 ± 0.72 at baseline which improved to 7.14 ± 1.09 at Day 49 and 6.85 ± 0.98 at Day 90 respectively in Arm-A (Teneligliptin 20 mg + Dapagliflozin 10 mg) subjects, The mean HbA1c (%) of the subjects was 8.44 ± 0.79 at baseline which improved to 7.85 ± 1.13 at Day 49 and 7.12 ± 0.90 at Day 90 respectively in Arm-B (Sitagliptin 100 mg + Dapagliflozin

DocUUID : b897a93b-83ba-4de2-a309-31160c3ed1c4

10 mg) subjects and The mean HbA1c (%) of the subjects was 8.81 ± 0.78 at baseline which improved to 7.57 ± 1.21 at Day 49 and 7.04 ± 0.92 at Day 90 respectively in Arm-C (Empagliflozin 25 mg + Linagliptin 5 mg) subjects. The mean FPG (mg/dl) was 144.00 ± 30.57 at baseline and improved to 118.22 ± 19.46 at Day 49 and 104.18 ± 13.89 at Day 90 in Arm-A (Teneligliptin 20 mg + Dapagliflozin 10 mg), mean FPG was 147.28 ± 45.68 at baseline which improved to 123.00 ± 23.96 at Day 49 and 113.82 ± 20.25 at Day 90 respectively in Arm-B (Sitagliptin 100 mg + Dapagliflozin 10 mg) subjects and the mean FPG (mg/dl) of the subjects was 147.29 ± 38.65 at baseline which improved to 115.20 ± 14.06 at Day 49 and 111.71 ± 18.54 at Day 90 respectively in Arm-C (Empagliflozin 25 mg + Linagliptin 5 mg) subjects The mean PPG (mg/dl) was 194.02 ± 47.17 at baseline and improved to 158.12 ± 31.68 at Day 49 and 155.19 ± 40.76 at day 90 in Arm-A (Teneligliptin 20 mg + Dapagliflozin 10 mg), mean PPG was 212.14 ± 77.62 at baseline which improved to 165.19 ± 34.68 at Day 49 and 145.06 ± 29.97 at Day 90 respectively in Arm-B (Sitagliptin 100 mg + Dapagliflozin 10 mg) subjects and The mean PPG (mg/dl) of the subjects was 195.80 ± 44.66 at baseline which improved to 160.02 ± 40.88 at Day 49 and 160.30 ± 43.93 at Day 90 respectively in Arm-C (Empagliflozin 25 mg + Linagliptin 5 mg) subjects.

Table-11 14: Summary Glycaemic results (FPG, PPG, HBA1c) with comparison between the group

Parameter	Visits	Arm-A (Teneligliptin 20 mg + Dapagliflozin 10 mg)		Arm-B (Sitagliptin 100 mg + Dapagliflozin 10 mg)		Arm-C (Empagliflozin 25 mg + Linagliptin 5 mg)		p-value
		n	Mean ± SD	n	Mean ± SD	n	Mean ± SD	
HbA1c	Visit 1 (Day -3 to Day 0)	30	8.47 ± 0.72	30	8.44 ± 0.79	30	8.81 ± 0.78	[a] 0.879 [b] 0.087 [c] 0.075
	Visit 6: (Day 49)	29	7.14 ± 1.09	28	7.85 ± 1.13	26	7.57 ± 1.21	[a] 0.019 [b] 0.175 [c] 0.384
	Visit 7: (Day 90)	29	6.85 ± 0.98	28	7.12 ± 0.90	25	7.04 ± 0.92	[a] 0.274 [b] 0.461 [c] 0.742
FPG	Visit 1 (Day -3 to Day 0)	30	144.00 ± 30.57	30	147.28 ± 45.68	30	147.29 ± 38.65	[a] 0.745 [b] 0.716 [c] 0.999
	Visit 6: (Day 49)	25	118.22 ± 19.46	22	123.00 ± 23.96	19	115.20 ± 14.06	[a] 0.461 [b] 0.553 [c] 0.205
	Visit 7: (Day 90)	29	104.18 ± 13.89	28	113.82 ± 20.25	25	111.71 ± 18.54	[a] 0.042 [b] 0.103 [c] 0.693
PPG	Visit 1 (Day -3 to Day 0)	30	194.02 ± 47.17	30	212.14 ± 77.62	30	195.80 ± 44.66	[a] 0.280 [b] 0.881 [c] 0.323
	Visit 6: (Day 49)	25	158.12 ± 31.68	28	165.19 ± 34.68	26	160.02 ± 40.88	[a] 0.441 [b] 0.854 [c] 0.619
	Visit 7: (Day 90)	29	155.19 ± 40.76	28	145.06 ± 29.97	25	160.30 ± 43.93	[a] 0.289 [b] 0.661 [c] 0.152

Note: *p-value is calculated using unpaired t-test. ([a]- Arm-A vs Arm-B, [b]- Arm-A vs Arm-C, [c]- Arm-B vs Arm-C*

Summary:

The mean HbA1c (%) of the subjects was 8.47 ± 0.72 between the groups at baseline which improved to 7.14 ± 1.09 at Day 49 and 6.85 ± 0.98 at Day 90 respectively in Arm-A (Teneligliptin 20 mg + Dapagliflozin 10 mg) subjects, The mean HbA1c (%) of the subjects was 8.44 ± 0.79 between the groups which improved to 7.85 ± 1.13 at Day 49 and 7.12 ± 0.90 at Day 90 respectively in Arm-B (Sitagliptin 100 mg + Dapagliflozin 10 mg) subjects and The mean HbA1c (%) of the subjects was

DocUUID : b897a93b-83ba-4de2-a309-31160c3ed1c4

8.81 ± 0.78 between the groups at baseline which improved to 7.57 ± 1.21 at Day 49 and 7.04 ± 0.92 at Day 90 respectively in Arm-C (Empagliflozin 25 mg + Linagliptin 5 mg) subjects. The mean FPG (mg/dl) was 144.00 ± 30.57 between the groups and improved to 118.22 ± 19.46 at Day 49 and 104.18 ± 13.89 at Day 90 in Arm-A (Teneligliptin 20 mg + Dapagliflozin 10 mg), 147.28 ± 45.68 between the groups at baseline which improved to 123.00 ± 23.96 at Day 49 and 113.82 ± 20.25 at Day 90 respectively in Arm-B (Sitagliptin 100 mg + Dapagliflozin 10 mg) subjects and The mean FPG (mg/dl) of the subjects was 147.29 ± 38.65 between the groups at baseline which improved to 115.20 ± 14.06 at Day 49 and 111.71 ± 18.54 at Day 90 respectively in Arm-C (Empagliflozin 25 mg + Linagliptin 5 mg) subjects The mean PPG (mg/dl) was 194.02 ± 47.17 between the groups at baseline and improved to 158.12 ± 31.68 at Day 49 and 155.19 ± 40.76 at day 90 in Arm-A (Teneligliptin 20 mg + Dapagliflozin 10 mg), 212.14 ± 77.62 between the groups at baseline which improved to 165.19 ± 34.68 at Day 49 and 145.06 ± 29.97 at Day 90 respectively in Arm-B (Sitagliptin 100 mg + Dapagliflozin 10 mg) subjects and The mean PPG (mg/dl) of the subjects was 195.80 ± 44.66 between the groups at baseline which improved to 160.02 ± 40.88 at Day 49 and 160.30 ± 43.93 at Day 90 respectively in Arm-C (Empagliflozin 25 mg + Linagliptin 5 mg) subjects.

Table-11 15: Summary Renal Parameters (UACR, eGFR, Serum Creatinine, BUN)

Parameter	Visits	Arm-A (Teneligliptin 20 mg + Dapagliflozin 10 mg)			Arm-B (Sitagliptin 100 mg + Dapagliflozin 10 mg)			Arm-C (Empagliflozin 25 mg + Linagliptin 5 mg)		
		n	Mean ± SD	p-value	n	Mean ± SD	p-value	n	Mean ± SD	p-value
**Urine Albumin Creatinine Ratio (UACR)**	Visit 1 (Day -3 to Day 0)	30	20.13 ± 37.46	-	30	23.18 ± 23.60	-	30	18.12 ± 13.30	-
	Visit 7: (Day 90)	29	41.94 ± 113.06	0.69	28	33.00 ± 66.87	0.952	25	56.53 ± 106.77	0.474
**Estimated Glomerular Filtration Rate (eGFR)**	Visit 1 (Day -3 to Day 0)	30	94.22 ± 16.54	-	30	98.55 ± 28.18	-	30	101.05 ± 23.28	-
	Visit 7: (Day 90)	29	101.24 ± 20.93	0.072	28	101.37 ± 21.21	0.241	25	100.98 ± 23.70	0.943
**Serum Creatinine**	Visit 1 (Day -3 to Day 0)	30	0.90 ± 0.19	-	30	0.86 ± 0.22	-	30	0.84 ± 0.25	-
	Visit 7: (Day 90)	29	0.83 ± 0.17	0.024	28	0.85 ± 0.16	0.788	25	0.85 ± 0.22	0.819
**Blood urea nitrogen (BUN)**	Visit 1 (Day -3 to Day 0)	30	13.68 ± 7.06	-	30	11.45 ± 2.77	-	30	14.69 ± 9.26	-
	Visit 7: (Day 90)	29	12.83 ± 2.70	0.681	28	13.78 ± 3.05	0.004	25	13.43 ± 3.20	0.916

Note: *N-Number of Subjects in Group; n-Number of samples in Group*; *p-value will be calculated using paired t-test.*

Summary:

Renal parameters of the subject were well maintained throughout the study duration. The Urine Albumin Creatinine Ratio (UACR) of the subjects was 20.13 ± 37.46 at baseline and 41.94 ± 113.06 at Day 90 in Arm-A (Teneligliptin 20 mg + Dapagliflozin 10 mg), 23.18 ± 23.60 at baseline which changed to 41.94 ± 113.06 at Day 90 in Arm-B (Sitagliptin 100 mg + Dapagliflozin 10 mg) subjects and 18.12 ± 13.30 at baseline which changed to 56.53 ± 106.77 at Day 90 in Arm-C (Empagliflozin 25 mg + Linagliptin 5 mg) subjects. The eGFR of the subjects was 94.22 ± 16.54 at baseline and 101.24 ± 20.93 at day 90 in Arm-A (Teneligliptin 20 mg + Dapagliflozin 10 mg), 98.55 ± 28.18 at baseline which changed to 101.37 ± 21.21 at Day 90 in Arm-B (Sitagliptin 100 mg + Dapagliflozin

DocUUID : b897a93b-83ba-4de2-a309-31160c3ed1c4

10 mg) subjects and 101.05 ± 23.28 at baseline which changed to 100.98 ± 23.70 at Day 90 in Arm-C (Empagliflozin 25 mg + Linagliptin 5 mg) subjects. The serum creatinine of the subjects was 0.90 ± 0.19 at baseline and 0.83 ± 0.17 at day 90 in Arm-A (Teneligliptin 20 mg + Dapagliflozin 10 mg), 0.86 ± 0.22 at baseline which changed to 0.85 ± 0.16 at Day 90 in Arm-B (Sitagliptin 100 mg + Dapagliflozin 10 mg) subjects and the 0.84 ± 0.25 at baseline which changed to 0.85 ± 0.22 at Day 90 in Arm-C (Empagliflozin 25 mg + Linagliptin 5 mg) subjects. The Blood urea nitrogen (BUN) of the subjects was 13.68 ± 7.06 at baseline and 12.83 ± 2.70 at day 90 in Arm-A (Teneligliptin 20 mg + Dapagliflozin 10 mg), 11.45 ± 2.77 at baseline which changed to 13.78 ± 3.05 at Day 90 in Arm-B (Sitagliptin 100 mg + Dapagliflozin 10 mg) subjects and the mean Blood urea nitrogen (BUN) of the subjects was 14.69 ± 9.26 at baseline which changed to 13.43 ± 3.20 at Day 90 in Arm-C (Empagliflozin 25 mg + Linagliptin 5 mg) subjects.

Table-11 16: Summary Renal Parameters (UACR, eGFR, Serum Creatinine, BUN) with comparison between the group

Parameter	Visits	Arm-A (Teneligliptin 20 mg + Dapagliflozin 10 mg)		Arm-B (Sitagliptin 100 mg + Dapagliflozin 10 mg)		Arm-C (Empagliflozin 25 mg + Linagliptin 5 mg)		p-value
		n	Mean ± SD	n	Mean ± SD	n	Mean ± SD	
Urine Albumin Creatinine Ratio (UACR)	Visit 1 (Day -3 to Day 0)	30	20.13 ± 37.46	30	23.18 ± 23.60	30	18.12 ± 13.30	[a] 0.707 [b] 0.784 [c] 0.311
	Visit 7: (Day 90)	29	41.94 ± 113.06	28	33.00 ± 66.87	25	56.53 ± 106.77	[a] 0.717 [b] 0.628 [c] 0.349
Estimated Glomerular Filtration Rate (eGFR)	Visit 1 (Day -3 to Day 0)	30	94.22 ± 16.54	30	98.55 ± 28.18	30	101.05 ± 23.28	[a] 0.471 [b] 0.196 [c] 0.71
	Visit 7: (Day 90)	29	101.24 ± 20.93	28	101.37 ± 21.21	25	100.98 ± 23.70	[a] 0.983 [b] 0.966 [c] 0.951
Serum Creatinine	Visit 1 (Day -3 to Day 0)	30	0.90 ± 0.19	30	0.86 ± 0.22	30	0.84 ± 0.25	[a] 0.419 [b] 0.259 [c] 0.702
	Visit 7: (Day 90)	29	0.83 ± 0.17	28	0.85 ± 0.16	25	0.85 ± 0.22	[a] 0.644 [b] 0.701 [c] 0.992
Blood urea nitrogen (BUN)	Visit 1 (Day -3 to Day 0)	30	13.68 ± 7.06	30	11.45 ± 2.77	30	14.69 ± 9.26	[a] 0.116 [b] 0.637 [c] 0.075
	Visit 7: (Day 90)	29	12.83 ± 2.70	28	13.78 ± 3.05	25	13.43 ± 3.20	[a] 0.222 [b] 0.466 [c] 0.688

Note: *p-value is calculated using unpaired t-test. ([a]- Arm-A vs Arm-B, [b]- Arm-A vs Arm-C, [c]- Arm-B vs Arm-C).*

Summary:

Renal parameters of the subject were well maintained throughout the study duration. The Urine Albumin Creatinine Ratio (UACR) of the subjects was 20.13 ± 37.46 between the groups at baseline and 41.94 ± 113.06 at Day 90 in Arm-A (Teneligliptin 20 mg + Dapagliflozin 10 mg), 23.18 ± 23.60 at baseline which improved to 41.94 ± 113.06 at Day 90 in Arm-B (Sitagliptin 100 mg + Dapagliflozin 10 mg) subjects and 18.12 ± 13.30 between the groups at baseline which improved to 56.53 ± 106.77 at Day 90 in Arm-C (Empagliflozin 25 mg + Linagliptin 5 mg) subjects. The eGFR of the subjects was 94.22 ± 16.54 between the groups at baseline and 101.24 ± 20.93 at day 90 in Arm-A (Teneligliptin 20 mg + Dapagliflozin 10 mg), 98.55 ± 28.18 at baseline which improved to 101.37 ± 21.21 at Day 90 in Arm-B (Sitagliptin 100 mg + Dapagliflozin 10 mg) subjects and 101.05 ± 23.28 between the groups at baseline which improved to 100.98 ± 23.70 at Day 90 in Arm-C (Empagliflozin 25 mg + Linagliptin 5 mg) subjects. The serum creatinine of the subjects was 0.90

DocUUID : b897a93b-83ba-4de2-a309-31160c3ed1c4

± 0.19 between the groups at baseline and 0.83 ± 0.17 at day 90 in Arm-A (Teneligliptin 20 mg + Dapagliflozin 10 mg), 0.86 ± 0.22 at baseline which improved to 0.85 ± 0.16 at Day 90 in Arm-B (Sitagliptin 100 mg + Dapagliflozin 10 mg) subjects and the 0.84 ± 0.25 between the groups at baseline which improved to 0.85 ± 0.22 at Day 90 in Arm-C (Empagliflozin 25 mg + Linagliptin 5 mg) subjects. The Blood urea nitrogen (BUN) of the subjects was 13.68 ± 7.06 between the groups at baseline and 12.83 ± 2.70 at day 90 in Arm-A (Teneligliptin 20 mg + Dapagliflozin 10 mg), 11.45 ± 2.77 at baseline which improved to 13.78 ± 3.05 at Day 90 in Arm-B (Sitagliptin 100 mg + Dapagliflozin 10 mg) subjects and The mean Blood urea nitrogen (BUN) of the subjects was 14.69 ± 9.26 between the groups at baseline which improved to 13.43 ± 3.20 at Day 90 in Arm-C (Empagliflozin 25 mg + Linagliptin 5 mg) subjects.

11.1. Efficacy Results

- The mean values of CGM parameters like Average Glucose, TIR, TBR, TAR, MAGE, LAGE, SD, CV, MPPGE, PP-1hr, PP-2hr were insignificantly different between the three arms at baseline.
- The mean values of CGM parameters were insignificantly different between the three arms in all parameters except Average Glucose, TAR, CV, post-prandial 1 hr glucose between Arm-A; Arm-B and CV between Arm-A; Arm-C at End of Phase I.
- The mean values of CGM parameters were insignificantly different between the three arms in all parameters except TAR, MPPGE between Arm-A; Arm-B at End of Phase II.
- In Arm-A, comparison of CGM parameters between baseline; End of Phase I showed statistically significant change in all parameters except TBR and CV.
- In Arm-B, comparison of CGM parameters between baseline; End of Phase I showed statistically significant change in all parameters except TIR, TBR, CV, MPPGE, post-prandial 1 hr glucose and post-prandial 2 hr glucose
- In Arm-C, comparison of CGM parameters between baseline; End of Phase I showed statistically significant change in all parameters except TBR
- In Arm-A and Arm-C, comparison of CGM parameters between baseline; End of Phase II showed statistically significant change in all parameters except TBR and CV.
- In Arm-B, comparison of CGM parameters between baseline; End of Phase II showed statistically significant change in all parameters except TBR, CV, and post-prandial 2 hr glucose
- The comparison of three CGM parameters MPPGE, post-prandial 1hr and 2hr glucose at baseline was insignificantly different between the three arms at breakfast, lunch and dinner.
- The comparison of three CGM parameters MPPGE, post-prandial 1hr and 2hr glucose at End of Phase I was insignificantly different between the three arms at breakfast, lunch and dinner.
- The comparison of three CGM parameters MPPGE, post-prandial 1hr and 2hr glucose at End of Phase II was insignificantly different between the three arms at dinner. However, MPPGE

DocUUID : b897a93b-83ba-4de2-a309-31160c3ed1c4

showed significant difference at breakfast between Arm-B; Arm-C and at lunch between Arm-A; Arm-C.

- At breakfast, the comparison of these three parameters MPPGE, post-prandial 1hr and 2hr glucose between baseline; End of Phase I showed statistically significant difference in Arm-A and Arm-C respectively, while none of the parameters showed significant difference in Arm-B.

- At lunch, the comparison of these three parameters MPPGE, post-prandial 1hr and 2hr glucose between baseline; End of Phase I showed no statistically significant difference in Arm-A, Arm-B and Arm-C respectively.

- At dinner, the comparison of these three parameters MPPGE, post-prandial 1hr and 2hr glucose between baseline; End of Phase I showed statistically significant difference only with post-prandial 1hr in Arm-A, while none of the parameters showed significant difference in Arm-B and Arm-C respectively.

- At breakfast, the comparison of these three parameters MPPGE, post-prandial 1hr and 2hr glucose between baseline; End of Phase II showed statistically significant difference in Arm-B and Arm-C respectively, while only MPPGE, post-prandial 2hr showed significant difference in Arm-A.

- At lunch, the comparison of these three parameters MPPGE, post-prandial 1hr and 2hr glucose between baseline; End of Phase II showed statistically significant difference with MPPGE, post-prandial 1hr in Arm-A, while none of the parameters showed significant difference in Arm-B and Arm-C respectively.

- At dinner, the comparison of these three parameters MPPGE, post-prandial 1hr and 2hr glucose between baseline; End of Phase II showed statistically significant difference only with post-prandial 1hr in Arm-A, while none of the parameters showed significant difference in Arm-B and Arm-C respectively.

- The mean HbA1c (%) of the subjects was 8.47 ± 0.72 at baseline which improved to 7.14 ± 1.09 at Day 49 and 6.85 ± 0.98 at Day 90 respectively in Arm-A (Teneligliptin 20 mg + Dapagliflozin 10 mg) subjects, The mean HbA1c (%) of the subjects was 8.44 ± 0.79 at baseline which improved to 7.85 ± 1.13 at Day 49 and 7.12 ± 0.90 at Day 90 respectively in Arm-B (Sitagliptin 100 mg + Dapagliflozin 10 mg) subjects and The mean HbA1c (%) of the subjects was 8.81 ± 0.78 at baseline which improved to 7.57 ± 1.21 at Day 49 and 7.04 ± 0.92 at Day 90 respectively in Arm-C (Empagliflozin 25 mg + Linagliptin 5 mg) subjects

- The mean FPG (mg/dl) was 144.00 ± 30.57 at baseline and improved to 118.22 ± 19.46 at Day 49 and 104.18 ± 13.89 at Day 90 in Arm-A (Teneligliptin 20 mg + Dapagliflozin 10 mg), 147.28 ± 45.68 at baseline which improved to 123.00 ± 23.96 at Day 49 and 113.82 ± 20.25 at Day 90 respectively in Arm-B (Sitagliptin 100 mg + Dapagliflozin 10 mg) subjects and The mean FPG (mg/dl) of the subjects was 147.29 ± 38.65 at baseline which improved to 115.20 ± 14.06 at Day 49 and 111.71 ± 18.54 at Day 90 respectively in Arm-C (Empagliflozin 25 mg + Linagliptin 5 mg) subjects

- The mean PPG (mg/dl) was 194.02 ± 47.17 at baseline and improved to 158.12 ± 31.68 at Day 49 and 155.19 ± 40.76 at day 90 in Arm-A (Teneligliptin 20 mg + Dapagliflozin 10 mg), 212.14

DocUUID : b897a93b-83ba-4de2-a309-31160c3ed1c4

± 77.62 at baseline which improved to 165.19 ± 34.68 at Day 49 and 145.06 ± 29.97 at Day 90 respectively in Arm-B (Sitagliptin 100 mg + Dapagliflozin 10 mg) subjects and The mean PPG (mg/dl) of the subjects was 195.80 ± 44.66 at baseline which improved to 160.02 ± 40.88 at Day 49 and 160.30 ± 43.93 at Day 90 respectively in Arm-C (Empagliflozin 25 mg + Linagliptin 5 mg) subjects.

- Renal parameters of the subject were well maintained throughout the study duration. The Urine Albumin Creatinine Ratio (UACR) of the subjects was 20.13 ± 37.46 at baseline and 41.94 ± 113.06 at Day 90 in Arm-A (Teneligliptin 20 mg + Dapagliflozin 10 mg), 23.18 ± 23.60 at baseline which changed to 41.94 ± 113.06 at Day 90 in Arm-B (Sitagliptin 100 mg + Dapagliflozin 10 mg) subjects and The mean Urine Albumin Creatinine Ratio (UACR) of the subjects was 18.12 ± 13.30 at baseline which changed to 56.53 ± 106.77 at Day 90 in Arm-C (Empagliflozin 25 mg + Linagliptin 5 mg) subjects

- The eGFR of the subjects was 94.22 ± 16.54 at baseline and 101.24 ± 20.93 at day 90 in Arm-A (Teneligliptin 20 mg + Dapagliflozin 10 mg), 98.55 ± 28.18 at baseline which improved to 101.37 ± 21.21 at Day 90 in Arm-B (Sitagliptin 100 mg + Dapagliflozin 10 mg) subjects and The mean eGFR of the subjects was 101.05 ± 23.28 at baseline which changed to 100.98 ± 23.70 at Day 90 in Arm-C (Empagliflozin 25 mg + Linagliptin 5 mg) subjects.

- The serum creatinine of the subjects was 0.90 ± 0.19 at baseline and 0.83 ± 0.17 at day 90 in Arm-A (Teneligliptin 20 mg + Dapagliflozin 10 mg), 0.86 ± 0.22 at baseline which improved to 0.85 ± 0.16 at Day 90 in Arm-B (Sitagliptin 100 mg + Dapagliflozin 10 mg) subjects and the mean serum creatinine of the subjects was 0.84 ± 0.25 at baseline which changed to 0.85 ± 0.22 at Day 90 in Arm-C (Empagliflozin 25 mg + Linagliptin 5 mg) subjects.

- The Blood urea nitrogen (BUN) of the subjects was 13.68 ± 7.06 at baseline and 12.83 ± 2.70 at day 90 in Arm-A (Teneligliptin 20 mg + Dapagliflozin 10 mg), 11.45 ± 2.77 at baseline which changed to 13.78 ± 3.05 at Day 90 in Arm-B (Sitagliptin 100 mg + Dapagliflozin 10 mg) subjects and the mean Blood urea nitrogen (BUN) of the subjects was 14.69 ± 9.26 at baseline which improved to 13.43 ± 3.20 at Day 90 in Arm-C (Empagliflozin 25 mg + Linagliptin 5 mg) subjects.

11.2. Efficacy Conclusions

Initiation of fixed dose combination (FDC) of Teneligliptin 20 mg + Dapagliflozin 10 mg, FDC of Sitagliptin 100 mg + Dapagliflozin 10 mg and FDC of Linagliptin 5 mg+ Empagliflozin 25 mg showed significant improvement in HbA1c, FPG and PPG from baseline to Day 49 and Day 90. The mean values of CGM parameters like Average Glucose, TIR, TBR, TAR, MAGE, LAGE, SD, CV, MPPGE, PP-1hr, PP-2hr were insignificantly different between the three arm. The comparison of three CGM parameters MPPGE, post-prandial 1hr and 2hr glucose at baseline was insignificantly different between the three arms at breakfast, lunch and dinner. Renal parameters of the subject were well maintained throughout the study duration.

DocUUID : b897a93b-83ba-4de2-a309-31160c3ed1c4

12. SAFETY ASSESSMENT

Table-12 3: Summary of Overall Adverse Events

Parameters	[AE's] n (%)
**Overall**	**[17] 10 (11.11 %)**
**Severity**	
**Mild**	[15] 9 (10.0 %)
**Moderate**	[2] 2 (2.22 %)
**Serious Adverse Event**	
**Yes**	-
**No**	-
**Relationship with the study Drug**	
Related	[4] 3 (3.33 %)
Not Related	[13] 7 (7.78 %)
**Outcome**	
Resolved	[17] 10 (11.11 %)

Note: *N-Number of Subjects (N=90); n-Number of Subjects with Adverse Events.*

Summary:

A total of 17 adverse events were reported in 10 subjects (11.11%) in the study. Among the 17 adverse events 4 events reported in 3 subjects (10.00%), 6 events reported in 4 subjects (13.33%) and 7 events reported in 3 subjects (3.33%) in Arm-A, Arm-B and Arm-C respectively. 4 AEs were related to the study drug, 7 AEs not related to the study drug. 15 AEs were mild in severity and 2 AEs were moderate in severity. All reported 17 AEs were resolved.

Table-12 2: Details of Overall Adverse Events

Event	Arm-A (Teneligliptin 20 mg + Dapagliflozin 10 mg)	Arm-B (Sitagliptin 100 mg + Dapagliflozin 10 mg)	Arm-C (Empagliflozin 25 mg + Linagliptin 5 mg)
	**[AE's] n (%)**	**[AE's] n (%)**	**[AE's] n (%)**
**Overall**	**[4] 3 (10.00%)**	**[6] 4 (13.33%)**	**[7] 3 (10.00%)**
Acidity Flatulence	-	-	[1] 1 (3.33%)
Common Cold	-	[1] 1 (3.33%)	-
Cough	-	[1] 1 (3.33%)	-
Fever	-	[1] 1 (3.33%)	[2] 2 (6.67%)
Genital Infection	-	[1] 1 (3.33%)	-
Headache	-	-	[1] 1 (3.33%)
Joint Pain	-	-	[1] 1 (3.33%)
Shivering	[1] 1 (3.33%)	-	-
Urinary tract infection	-	[1] 1 (3.33%)	-
Weakness	[3] 3 (10.00%)	[1] 1 (3.33%)	[2] 2 (6.67%)

Note: *(N=30)-Total Number of Subjects in Group; n-Number of Subjects with Adverse Events in Group, AE- Number of Subjects with Adverse Events.*

Summary:

DocUUID : b897a93b-83ba-4de2-a309-31160c3ed1c4

A. total of 17 adverse events were reported in 10 subjects (11.11%) in the study. Among the 17 adverse events, 1 event of acidity flatulence in 1 subject (1.11%), 1 event of common cold in 1 subject (1.11%), 1 event of cough in 1 subject (1.11%), 3 events of fever were reported in 3 subjects (3.33%), 1 event of genital infection was reported in 1 subject (1.11%), 1 event of headache in 1 subject (1.11%), 1 event of joint pain in 1 subject (1.11%), 1 event of shivering in 1 subject (1.11%), 1 event of urinary tract infection in 1 subject (1.11%) and 6 events of weakness were reported in 6 subjects (6.67%).

Table- 12 3: Severity of Overall Adverse Events

Severity	Arm-A (Teneligliptin 20 mg + Dapagliflozin 10 mg)	Arm-B (Sitagliptin 100 mg + Dapagliflozin 10 mg)	Arm-C (Empagliflozin 25 mg + Linagliptin 5 mg)
	**[AE's] n (%)**	**[AE's] n (%)**	**[AE's] n (%)**
Mild	[4] 3 (10.00%)	[5] 4 (13.33%)	[6] 2 (6.67%)
Moderate	-	[1] 1 (3.33%)	[1] 1 (3.33%)

Note: *(N=30)-Total Number of Subjects in Group; n-Number of Subjects with Adverse Events in Group, AE- Number of Subjects with Adverse Events.*

Summary:

Among the 17 adverse events, the severities of 15 events in 9 (10.0%) subjects were reported as mild and 2 events in 2 (2.22%) subjects were reported as moderate.

Table-12 4: Relationship of Overall Adverse Events

Relation	Arm-A (Teneligliptin 20 mg + Dapagliflozin 10 mg)	Arm-B (Sitagliptin 100 mg + Dapagliflozin 10 mg)	Arm-C (Empagliflozin 25 mg + Linagliptin 5 mg)
	**[AE's] n (%)**	**[AE's] n (%)**	**[AE's] n (%)**
Related	[3] 2 (6.67%)	[1] 1 (3.33%)	-
Not related	[1] 1 (3.33%)	[5] 3 (10.00%)	[7] 3 (10.00%)

Note: *(N=30)-Total Number of Subjects in Group; n-Number of Subjects with Adverse Events in Group, AE- Number of Subjects with Adverse Events.*

Summary:

The relationship of 4 events, (Arm A: 2 Weakness and 1 shivering, Arm B: Genital infection) reported in 3 (3.33%) subjects were related to study drug. For remaining 13 events in 7 (7.78%) subjects were unrelated to Study drug.

Table-12 5: Summary of Action Taken for Adverse Events

Action taken	Arm-A (Teneligliptin 20 mg + Dapagliflozin 10 mg)	Arm-B (Sitagliptin 100 mg + Dapagliflozin 10 mg)	Arm-C (Empagliflozin 25 mg + Linagliptin 5 mg)
	**[AE's] n (%)**	**[AE's] n (%)**	**[AE's] n (%)**
IP Temporary interrupted	-	[1] 1 (3.33%) *	-
Medication given	[1] 1 (3.33%)	[5] 3 (10.00%)	[7] 3 (10.00%)
None	[3] 2 (6.67%)	-	-

DocUUID : b897a93b-83ba-4de2-a309-31160c3ed1c4

Note: *(N=30)-Total Number of Subjects in Group; n-Number of Subjects with Adverse Events in Group, AE- Number of Subjects with Adverse Events, *Antifungal medications were given to the subject for the reported AE*

Summary:

A total of 17 adverse events were reported in 10 subjects (11.11%) in the study. Among the 17 events 1 AE of 1 subject (3.33%) action taken was IP temporary interrupted. Medication was given for 1 AE of 1 subject (3.33%) in Arm-A, 5 AEs of 3 subjects (10.00%) in Arm-B and 7 AEs of 3 subjects (10.00%) in Arm-C. 3 AEs of 2 subjects (6.67%) in Arm-A Action taken was none.

Table-12 6: Outcome of Overall Adverse Events

Outcome	Arm-A (Teneligliptin 20 mg + Dapagliflozin 10 mg)	Arm-B (Sitagliptin 100 mg + Dapagliflozin 10 mg)	Arm-C (Empagliflozin 25 mg + Linagliptin 5 mg)
	**[AE's] n (%)**	**[AE's] n (%)**	**[AE's] n (%)**
Completely Resolved	[4] 3 (10.00%)	[6] 4 (13.33%)	[7] 3 (10.00%)

Note: *(N=30)-Total Number of Subjects in Group; n-Number of Subjects with Adverse Events in Group, AE- Number of Subjects with Adverse Events.*

Summary:

The outcome of all 17 adverse events reported in 10 subjects (11.11%) were resolved.

12.1. Extent of Exposure

A total of 90 subjects were enrolled into the study and 87 subjects were exposed to the study drug.

12.2. Adverse events

12.2.1. Brief Summary of Adverse Events

A total of 17 adverse events were reported in 10 subjects (11.11%) in the study. Among the 17 adverse events 4 events reported in 3 subjects (10.00%), 6 events reported in 4 subjects (13.33%) and 7 events reported in 3 subjects (3.33%) in Arm-A, Arm-B and Arm-C respectively.

Among the 17 adverse events, 1 event of acidity flatulence in 1 subject (1.11%), 1 event of common cold in 1 subject (1.11%), 1 event of cough in 1 subject (1.11%), 3 events of fever were reported in 3 subjects (3.33%), 1 event of genital infection was reported in 1 subject (1.11%), 1 event of headache in 1 subject (1.11%), 1 event of joint pain in 1 subject (1.11%), 1 event of shivering in 1 subject (1.11%), 1 event of urinary tract infection in 1 subject (1.11%) and 6 events of weakness were reported in 6 subjects (6.67%).

No SAEs and deaths were reported in the study.

12.2.2. Display of Adverse Events

A total of 17 adverse events were reported in 10 subjects (11.11%) in the study. Among the 17 adverse events 4 events reported in 3 subjects (10.00%), 6 events reported in 4 subjects (13.33%) and 7 events reported in 3 subjects (3.33%) in Arm-A, Arm-B and Arm-C respectively.

DocUUID : b897a93b-83ba-4de2-a309-31160c3ed1c4

Among the 17 adverse events, 1 event of acidity flatulence in 1 subject (1.11%), 1 event of common cold in 1 subject (1.11%), 1 event of cough in 1 subject (1.11%), 3 events of fever were reported in 3 subjects (3.33%), 1 event of genital infection was reported in 1 subject (1.11%), 1 event of headache in 1 subject (1.11%), 1 event of joint pain in 1 subject (1.11%), 1 event of shivering in 1 subject (1.11%), 1 event of urinary tract infection in 1 subject (1.11%) and 6 events of weakness were reported in 6 subjects (6.67%).
Among the 17 adverse events, the severities of 15 events in 9 (10.0%) subjects were reported as mild and 2 events in 2 (2.22%) subjects were reported as moderate. The relationship of 4 events reported in 3 (3.33%) subjects were related to study drug while for 13 events in 7 (7.78%) subjects were unrelated to Study drug. The outcome of all 17 adverse events reported in 10 subjects (18.89%) were resolved.
No SAEs and deaths were reported in the study.

12.2.3. Analysis of Adverse Events

A total of 17 events were reported in the study.
Among the 17 adverse events, 1 event of acidity flatulence in 1 subject (1.11%), 1 event of common cold in 1 subject (1.11%), 1 event of cough in 1 subject (1.11%), 3 events of fever were reported in 3 subjects (3.33%), 1 event of genital infection was reported in 1 subject (1.11%), 1 event of headache in 1 subject (1.11%), 1 event of joint pain in 1 subject (1.11%), 1 event of shivering in 1 subject (1.11%), 1 event of urinary tract infection in 1 subject (1.11%) and 6 events of weakness were reported in 6 subjects (6.67%).

Among the 17 adverse events, the severities of 15 events in 9 (10.0%) subjects were reported as mild and 2 events in 2 (2.22%) subjects were reported as moderate. The relationship of 4 events reported in 3 (3.33%) subjects were related to study drug while for 13 events in 7 (7.78%) subjects were unrelated to Study drug. The outcome of all 17 adverse events reported in 10 subjects (11.11%) were resolved.

No SAEs and deaths were reported in the study.

12.2.4. Listing of Adverse Events by Subjects

Subject-wise listing of pre-treatment adverse events is included in Appendix in Section 16.2

12.3. Deaths, Other Serious and Other Significant Adverse Events

No Deaths, Other Serious and Other Significant Adverse Events were reported during the study.

12.3.1. Listing of Deaths, Other Serious and Other Significant Adverse Events

No Deaths, Other Serious and Other Significant Adverse Events were reported during the study.

12.3.1.1. Deaths

No Deaths were reported during the study.

12.3.1.2. Other Serious Adverse Events

No other Serious Adverse Events were reported during the study

DocUUID : b897a93b-83ba-4de2-a309-31160c3ed1c4

12.3.1.3. Other Significant Adverse Events

No other significant adverse events were reported in the study

12.3.2. Narratives of Deaths, Serious Other Significant Adverse Events

No Deaths, Other Serious and Other Significant Adverse Events were reported during the study.

12.3.3. Analysis and Discussion of Deaths, Other Serious and other Significant Adverse Events

Not applicable

12.4. Clinical Laboratory Evaluation

Not applicable

12.5. Safety Conclusions

A total of 17 adverse events were reported in 10 subjects (11.11%) in the study. Among the 17 adverse events 4 events reported in 3 subjects (10.00%), 6 events reported in 4 subjects (13.33%) and 7 events reported in 3 subjects (3.33%) in Arm-A, Arm-B and Arm-C respectively.

Among the 17 adverse events, 1 event of acidity flatulence in 1 subject (1.11%), 1 event of common cold in 1 subject (1.11%), 1 event of cough in 1 subject (1.11%), 3 events of fever were reported in 3 subjects (3.33%), 1 event of genital infection was reported in 1 subject (1.11%), 1 event of headache in 1 subject (1.11%), 1 event of joint pain in 1 subject (1.11%), 1 event of shivering in 1 subject (1.11%), 1 event of urinary tract infection in 1 subject (1.11%) and 6 events of weakness were reported in 6 subjects (6.67%).

Among the 17 adverse events, the severities of 15 events in 9 (10.0%) subjects were reported as mild and 2 events in 2 (2.22%) subjects were reported as moderate. The relationship of 4 events reported in 3 (3.33%) subjects were related to study drug while for 13 events in 7 (7.78%) subjects were unrelated to Study drug. The outcome of all 17 adverse events reported in 10 subjects (11.11%) were resolved.

No SAEs and deaths were reported in the study.

DocUUID : b897a93b-83ba-4de2-a309-31160c3ed1c4

13. Overall Conclusions

SGLT2i such as dapagliflozin, by specifically targeting the kidney, inhibit glucose reabsorption at the proximal tubule and thereby promote glucosuria, an effect independent of insulin. Because of the progressive deterioration of beta-cell function that characterizes T2DM, a pharmacological mechanism of action that is independent of pancreatic beta-cell function makes SGLT2i an appropriate option for patients with advanced T2DM, particularly if their glycaemic control is inadequate with other oral glucose-lowering agents. By promoting glucosuria and reducing hyperglycaemia, SGLT2i dampen glucotoxicity, which indirectly results in an improvement of beta-cell function and peripheral insulin sensitivity. However, treatment with SGLT2i resulted in an increase in plasma glucagon concentrations, which was accompanied by a substantial increase in endogenous (hepatic) glucose production. The latter has been estimated to offset approximately half of the glucose excreted in the urine as a result of SGLT2i.

DPP-4i such as teneligliptin enhance postprandial insulin secretion and suppress glucagon secretion by preventing the degradation of endogenously released incretin hormones [glucagon-like peptide (GLP)-1 and glucose-dependent insulinotropic polypeptide (GIP)], two intestinal peptides whose concentrations physiologically increase after food intake. Of major interest, DPP- 4 inhibitors stimulate insulin secretion and inhibit glucagon secretion in a glucose-dependent manner, thus reducing hyperglycaemia while minimizing hypoglycaemia.

Thus, the addition of a teneligliptin which inhibits glucagon and stimulates insulin secretion may have the potential to block the increase in endogenous glucose production and enhance the glucose-lowering ability of dapagliflozin. Taken together these findings suggest that the combination of teneligliptin with dapagliflozin would potentially provide additional help to individuals with T2DM in reaching their glycaemic goal.

Also, the patients with T2DM require strict control of GV especially post-prandial hyperglycemia, which directly correlate with CV morbidity and mortality associated with diabetes and its progression. Our study demonstrated that fixed dose combination (FDC) of Teneligliptin + Dapagliflozin was found to be non-inferior to to FDC of Sitagliptin + Dapagliflozin and FDC of Linagliptin+ Empagliflozin. All 3 groups showed improvement in CGM parameters, HbA1c, FPG, PPG and maintained the renal parameters.

Thus FDC Teneligliptin + Dapagliflozin offers a promising treatment option to achieve optimal glucose for patients with T2DM who are not controlled by mono or dual therapy.

DocUUID : b897a93b-83ba-4de2-a309-31160c3ed1c4

14. TABLES AND FIGURES REFERRED IN THE STUDY BUT NOT INCLUDED IN THE TEXT

All tables are presented under Section 10 and 11

14.1. Demographic Data

Demographic data is provided in Table 10.

14.2. Efficacy Data

Efficacy data provide in Section 11.2.1

14.3. Safety Data

14.3.1. Displays of Adverse Events

Safety data and adverse events related tables were provided in Section 11.1.

14.3.2. Listings of Deaths, Other Serious and Significant Adverse Events

No Deaths, Other Serious and Significant Adverse Events were reported during the study.

14.3.3. Narratives of Deaths and Other Serious Adverse Events

No Deaths, Other Serious Adverse Events were reported during the study.

DocUUID : b897a93b-83ba-4de2-a309-31160c3ed1c4

15. REFERENCES

1. ICH Harmonies Tripartite Guideline, Guideline for Good Clinical Practice, E6 (R2).

2. Ethical guidelines for biomedical research on human participants, ICMR (Indian Council of Medical Research.

3. MINISTRY OF HEALTH AND FAMILY WELFARE, New Drugs and Clinical Trials Rules, 2019 [Internet], G.S.R.227(E) THE GAZETTE OF INDIA: EXTRAORDINARY; 2019 p. 147–264. Available from:

   https://cdsco.gov.in/opencms/opencms/system/modules/CDSCO.WEB/elements/download_file_division.jsp?num_id=NDI2MQ=.

4. Good clinical practices for Clinical Research in India.

5. International conference on harmonisation of technical requirements for registration of pharmaceuticals for human use ICH harmonised tripartite guideline structure and content of clinical study reports E3

6. International conference on harmonisation of technical requirements for registration of pharmaceuticals for human use ich harmonised tripartite guideline statistical principles for clinical trials E9.

7. V. Mohan, S. Sandeep, R. Deepa et al; Epidemiology of type 2 diabetes: Indian scenario;Indian J Med Res 125, March 2007, pp 217-230

8. Turmner RC et al. Glycemic control with diet, sulfonylurea, metformin, or insulin inpatients with type 2 diabetes mellitus: progressive requirement for multiple therapies (UKPDS 49). UK Prospective Diabetes Study (UKPDS) Group JAMA 1999; 281(21):2005-12.

9. Satya Krishna SV, Kota SK, Modi KD. Glycemic variability: Clinical implications. Indian Journal of Endocrinology and Metabolism. 2013;17(4):611-619.

10. Wilmot EG, Choudhary P, Leelarathna L, et al. Glycaemic variability: The under- recognized therapeutic target in type 1 diabetes care. Diabetes Obes Metab. 2019;21(12):2599-2608.

11. Chadha, M., Das, A.K., Deb, P. et al. Expert Opinion: Optimum Clinical Approach to Combination-Use of SGLT2i + DPP4i in the Indian Diabetes Setting. Diabetes Ther 13, 1097–1114 (2022).

DocUUID : b897a93b-83ba-4de2-a309-31160c3ed1c4

16. APPENDICES

16.1. Study Information

16.1.1. Protocol and Protocol Amendments

16.1.2. Sample Case Report Forms

16.1.3. List of IECs/IRBs, and Representative Sample Informed Consent Forms

16.1.4. Curriculum Vitae (CV) or Equivalent Summaries of Training and Experience Relevant to the Performance of the Clinical Study

16.1.5. Signature Page

16.1.6. Listing of Subjects Receiving Investigational Product(s) from Specific Batches, where More Than One Batch was Used

16.1.7. Randomisation Scheme and Codes (Subject Identification and Intervention Assigned)

16.1.8. Audit Certificates (if available)

16.1.9. Documentation of Statistical Method

16.1.10. Documentation of Inter-laboratory Standardisation Methods and Quality Assurance Procedures If Used

16.1.11. Publications Based on the Study

16.1.12. Important Publications Referenced in the Report

16.2. Subject Data Listings

DocUUID : b897a93b-83ba-4de2-a309-31160c3ed1c4
